# Efficient wettability-controlled electroreduction of CO_2_ to CO at Au/C interfaces

**DOI:** 10.1038/s41467-020-16847-9

**Published:** 2020-06-15

**Authors:** Run Shi, Jiahao Guo, Xuerui Zhang, Geoffrey I. N. Waterhouse, Zhaojun Han, Yunxuan Zhao, Lu Shang, Chao Zhou, Lei Jiang, Tierui Zhang

**Affiliations:** 1 Key Laboratory of Photochemical Conversion and Optoelectronic Materials, Technical Institute of Physics and Chemistry, Chinese Academy of Sciences, Beijing, 100190 China; 2 Center of Materials Science and Optoelectronics Engineering, University of Chinese Academy of Sciences, Beijing, 100049 China; 3 School of Chemical Sciences, The University of Auckland, Auckland, 1142 New Zealand; 4 CSIRO Manufacturing, New South Wales, 2070 Australia; 5 Key Laboratory of Bio-inspired Materials and Interfacial Science, Technical Institute of Physics and Chemistry, Chinese Academy of Sciences, Beijing, 100190 China

**Keywords:** Electrocatalysis, Energy, Electrocatalysis, Characterization and analytical techniques

## Abstract

The electrochemical CO_2_ reduction reaction (CO_2_RR) represents a very promising future strategy for synthesizing carbon-containing chemicals in a more sustainable way. In spite of great progress in electrocatalyst design over the last decade, the critical role of wettability-controlled interfacial structures for CO_2_RR remains largely unexplored. Here, we systematically modify the structure of gas-liquid-solid interfaces over a typical Au/C gas diffusion electrode through wettability modification to reveal its contribution to interfacial CO_2_ transportation and electroreduction. Based on confocal laser scanning microscopy measurements, the Cassie-Wenzel coexistence state is demonstrated to be the ideal three phase structure for continuous CO_2_ supply from gas phase to Au active sites at high current densities. The pivotal role of interfacial structure for the stabilization of the interfacial CO_2_ concentration during CO_2_RR is quantitatively analysed through a newly-developed in-situ fluorescence electrochemical spectroscopic method, pinpointing the necessary CO_2_ mass transfer conditions for CO_2_RR operation at high current densities.

## Abstract (web summary)

Great advances have been made in CO_2_ electroreduction, however, the role of wettability-controlled interfacial structures remains poorly understood. Here, the authors apply confocal laser scanning microscopy to gain deeper understanding of these phenomena in gas diffusion electrodes.

## Introduction

Anthropogenic CO_2_ emissions arising from the chemical industry (e.g. cement and ammonia synthesis) and the combustion of fossil fuels for electricity generation and transportation are causative effects of global warming. One of the most promising approaches to reduce CO_2_ emissions is to capture CO_2_ and transform it into valuable commodity chemicals and hydrocarbon fuels^1,2^. A wide variety of technologies are currently being explored for achieving this, amongst which the electrochemical CO_2_ reduction reaction (CO_2_RR) is arguably the most promising, especially when the electricity used to drive the reduction is generated sustainably (i.e. from photovoltaics, hydro or wind turbines, geothermal power stations, etc.)^3–5^.

Over the past decade, enormous effort has been directed towards the design of electrocatalysts with improved CO_2_RR efficiencies^6–9^. Most studies have used an electrochemical cell based on a liquid–solid double phase contact (DPC) system, utilizing CO_2_ dissolved in the electrolyte. However, hydrogen evolution as competition is dominant for most DPC systems at high current densities as a result of fast water reduction kinetics and CO_2_ mass-transfer limitations (poor CO_2_ solubility and a low CO_2_ diffusion coefficient in liquid electrolytes)^10–15^. It is well known that the diffusion coefficient of CO_2_ in the gas phase (~0.1 cm^2^ s^−1^) is approximately four orders of magnitude higher than that of CO_2_ in the liquid phase^16,17^, thereby making CO_2_ in gas phase a more promising source for electrochemical CO_2_RR. Since gas-phase CO_2_ itself cannot partake in the electrochemical reaction in the absence of aqueous media, researchers have focussed attention on the development of three-phase contact (TPC) systems for CO_2_RR, wherein gaseous CO_2_, the electrolyte and the electrocatalyst are all in intimate contact^18–22^. TPC systems offer many advantages for electrochemical CO_2_RR, for example allowing the use of high pH electrolytes (that cannot readily be applied in DPC systems) to promote CO_2_RR electron transfer kinetics^23–25^. By this approach, efficient CO_2_ electrolysis to valuable products (e.g. CO, formic acid) can be achieved at high current densities, the use of the latter being an essential requirement for potential industrial scale applications^26–28^. However, due to the severe non-Faradaic consumption of CO_2_ in alkali electrolytes, recent efforts have been focused on the structure and micro-environment (i.e. layer thickness, electric field, pressure, etc.) of electrodes to increase the effective CO_2_ concentration at interfaces^8,29,30^. However, studying gas–liquid–solid three-phase interfaces is challenging, with knowledge being extremely limited about interfacial structures and CO_2_ transport behaviour under nonequilibrium conditions (i.e. conditions where the rate of CO_2_ consumption by electrochemical reaction and CO_2_ supply from bulk to the surface of the electrodes should be simultaneously considered).

The wettability of gas–liquid–solid interfaces has been extensively explored in the past few years^31–33^, providing new understanding of the mechanisms of wettability-controlled electrochemical reactions. It has been shown that the catalytic rate of various electrochemical reactions that involve a gas-phase reactant, such as the oxygen reduction and hydrogen evolution reactions, have an intimate relationship to the surface wettability of electrodes^34–37^. Through internal contact angle and microscopic analysis, the wetting property of gas diffusion layers (GDL) have recently been investigated^38–40^. In theory, variations of wettability over three-phase interfaces can dramatically alter the interfacial transportation behaviour of gaseous reactants/products and the contact between catalytic sites and ions in electrolyte, thus influencing gas diffusion and electron transfer processes as the rate determining steps in electrochemical reaction kinetics^41^. Accordingly, through wettability control it should be possible to simplify the complicated variables in TPC systems to independently investigate the relationship between interfacial structures, CO_2_ transportation and CO_2_ electroreduction, obtaining necessary information for the rational design of more efficient CO_2_RR systems.

Here we use a typical Au/C electrode as a model to demonstrate the role of wettability to electrochemical CO_2_RR, with the results expected to be transferrable to other gas diffusion electrode systems. By applying confocal laser scanning microscopy (CLSM) to image three-phase interfaces on Au/C electrode with interfacial wettabilities ranging from superhydrophobic to hydrophilic, we reveal the importance of the Cassie-Wenzel coexistence wetting state for promoting interfacial CO_2_ transportation and maintaining close contact between catalytic active sites and the electrolyte. Time-dependent in situ fluorescence electrochemical spectroscopy (FES) is developed to quantitatively analyse the interfacial CO_2_ transportation process during CO_2_RR. Our findings show that in TPC systems, the electrochemical CO_2_RR efficiency at large current densities is greatly influenced by the CO_2_ concentration at interfaces, which is fundamentally determined by the efficiency of CO_2_ mass transfer over wettability-controlled interface structures.

## Results

### Characterization of the Au/C electrodes

**Fig. 1:**
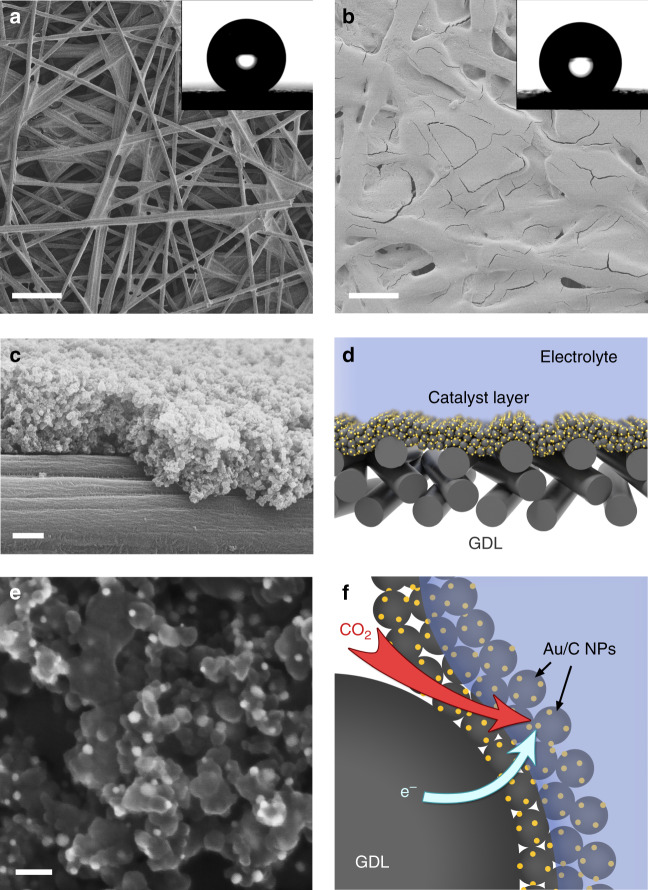
Structural characterization of the Au/C electrode. **a** SEM image of a PTFE-modified carbon fibre GDL, scale bar: 100 μm. **b** SEM image of the electrode after coating with an Au/C NPs film, scale bar: 100 μm. Insets in (**a**–**b**) show photographs of water droplets on each electrode. **c** Cross-sectional SEM image of the Au/C electrode, scale bar: 500 nm. **d** Schematic illustration of the TPC electrochemical cathode. **e** SEM image of the Au/C electrode at high magnification, scale bar: 50 nm. **f** Schematic illustration of the gas–liquid–solid three-phase interfaces of a TPC system for electrochemical CO_2_RR. Source data are provided as a Source data file.

Gold-based nanostructures are reported to offer efficient and stable CO_2_RR activities, along with high selectivity for the production of CO^42–47^. In this work, we prepared monodisperse gold nanoparticles (Au NPs) with diameters of 8.95 ± 0.52 nm, then mixed these with carbon black to produce a cathodic catalyst (Au/C NPs). Detailed characterization data for both the Au NPs and Au/C NPs are provided in Supplementary Fig. 1 (see also the Methods section for the synthesis of these materials). The Au/C electrodes were then prepared by depositing the Au/C NPs as a thin film onto a polytetrafluoroethylene (PTFE)-modified carbon fibre paper. Figure 1a shows a typical scanning electron microscopy (SEM) image of the PTFE-modified carbon fibre paper, which possessed an external water contact angle (CA) of 151 ± 2° and was used here as a superhydrophobic porous GDL. As shown in Fig. 1b, after the deposition of Au/C NPs onto the PTFE-modified GDL, the water CA decreased to 135 ± 3°. The Au/C NPs catalyst layer with an average thickness of 1.2 ± 0.1 μm is supported by the carbon fibres without blocking the internal pores of the GDL (Fig. 1c, d and Supplementary Fig. 2). This architecture is crucial as rapid transport of gaseous CO_2_ from the bulk gas phase to the surface of the Au/C NPs through the porous electrode was required to achieve a stable interfacial CO_2_ concentration when operating at high current densities (Fig. 1e, f).

### Wettability-controlled electrochemical CO_2_RR

**Fig. 2:**
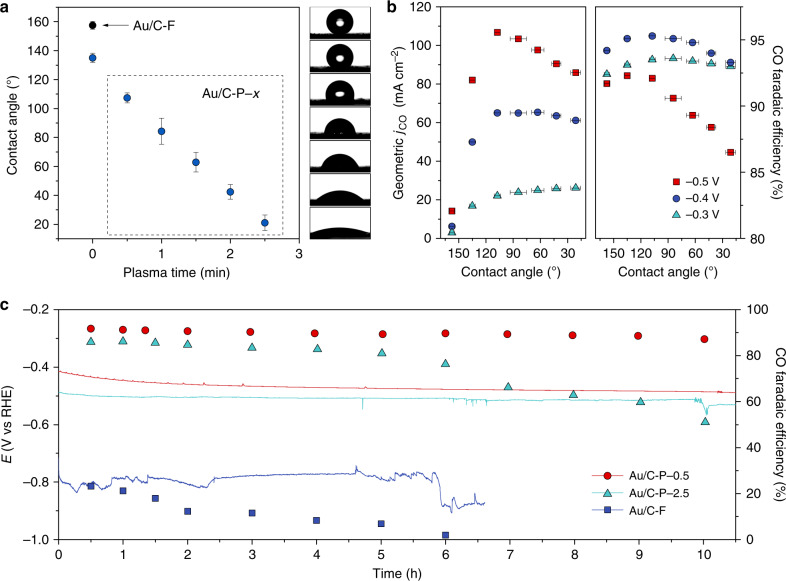
Effect of interfacial wettability on electrochemical CO_2_RR performance. **a** Plot of average water droplet contact angles on different Au/C electrodes and photographs of water droplets on each electrode. **b** Geometric *j*_CO_ and CO Faradaic efficiency of Au/C electrodes with various water CAs at −0.3, −0.4 and −0.5 V vs. RHE. Error bars represent the standard deviation of three independent experiments. **c** Cathode chronopotentiometry tests for Au/C-F, Au/C-P-0.5 and Au/C-P-2.5 in 1 M KOH at a constant current density of 100 mA cm^−2^ (without *i*R correction). Source data are provided as a Source data file.

We then prepared Au/C electrodes with a range of wettabilities through surface modifications of the catalyst layer. As is shown in Fig. 2a, one modification involved coupling a fluorine-terminated silane to the carbon black in Au/C catalyst layer (the resulting electrode is denoted as Au/C-F)^48^, thus producing a superhydrophobic electrode (water CA of 158 ± 3°). Conversely, other electrodes were prepared containing more oxygen groups on the surface of carbon black via air plasma treatments (the resulting electrodes were labelled as Au/C-P-*x*, where *x* represents the plasma treatment time in min)^49^. Fourier-transform infrared spectroscopy (FTIR) revealed that the amount of surface oxygen-containing functional groups on the electrodes increased with plasma treatment time (Supplementary Fig. 3). This gave electrodes that were more hydrophilic, with water CAs decreasing from 107 ± 3° (Au/C-P-0.5) to 21 ± 5° (Au/C-P-2.5). Through X-ray photoelectron spectroscopy (XPS) and Au L_3_-edge X-ray absorption fine structure (XAFS) characterization studies, we demonstrated that the surface modifications had little influence on the chemical composition or structure of the Au nanoparticles (Supplementary Figs. 4 and 5).

The electrochemical CO_2_RR performance of the different Au/C electrodes in TPC systems were investigated using a gas-phase-connected H-type electrochemical cell, using 1 M KOH as electrolyte (see Methods section). The potentials were referenced to the reversible hydrogen electrode (RHE). The linear sweep voltammetry (LSV) curves and chronoamperometry tests for the Au/C electrodes show an intimate relationship with the surface modifications, amongst which Au/C-P-0.5 exhibited the best performance (Supplementary Fig. 6). As seen in Fig. 2b, the wettability of Au/C catalyst layer greatly impacted the CO partial current density (*j*_CO_) and CO Faradaic efficiency, especially under large applied bias potentials. For example, at −0.3 V, *j*_CO_ gradually increased as the catalyst layer becomes more hydrophilic, which can be attributed to the increased electrochemical surface area (ECSA) of the Au/C electrodes (Supplementary Figs. 7–9)^50^. For CO_2_RR at −0.4 V, slightly decreased *j*_CO_ and CO Faradaic efficiencies were observed for electrodes with CA < 60°. At −0.5 V, the *j*_CO_ reached 106.7 mA cm^−2^ for Au/C-P-0.5 with a CA = 107 ± 3°, followed by a sharp reduction to 85.9 mA cm^−2^ for Au/C-P-2.5 with a CA = 21 ± 5°. Ongoing from Au/C-P-0.5 to Au/C-P-2.5, the CO Faradaic efficiency decreased from 92.1 to 86.5%. Based on the small overpotential and high CO Faradaic efficiency, Au/C-P-0.5 afforded excellent cathodic energy efficiency for CO production amongst reported gold-based electrochemical CO_2_RR systems (Supplementary Fig. 10 and Table 1). To probe the long-term stability, the CO_2_RR performance of three typical electrodes (i.e. Au/C-F, Au/C-P-0.5 and Au/C-P-2.5) were tested at a constant current density of 100 mA cm^−2^. As shown in Fig. 2c, the three electrodes with different wettabilities exhibited very different cathodic potentials and CO_2_RR stabilities. For Au/C-F, the superhydrophobic properties of the electrode gave a negative potential with very low CO Faradaic efficiency (decreasing from 23.2 to 1.9% after 6-h operation), implying poor contact between catalyst and electrolyte. The water CAs of both the front side and reverse side of the Au/C-F electrode decreased by more than 20° after the test, indicating the superhydrophobic property was not stable at such negative potentials (Supplementary Fig. 11). On the contrary, the cathodic potential of Au/C-P-0.5 was maintained at −0.47 ± 0.02 V during the stability test. The CO Faradaic efficiency decreased slightly from 91.8 to 87.3% over 10-h operation, which is explained by the increased hydrophilicity of the catalyst layer (the water CA decreased to 74°). It is worth noting that the reverse side of the Au/C-0.5 retained its superhydrophobic property. For Au/C-P-2.5, the water CA of the reverse side decreased slightly to 140° after the stability test. This indicates that the electrolyte penetrated through the hydrophilic catalyst layer and partially blocked the pores of GDL, thus decreasing the CO Faradaic efficiency from 86.0 to 51.0% after 10-h operation. Regulating the initial wettability of the catalyst layer is therefore significant for reducing the CO_2_RR overpotential and improving the CO_2_RR stability when operating at high current densities.

### Wettability-controlled TPC interfaces

**Fig. 3:**
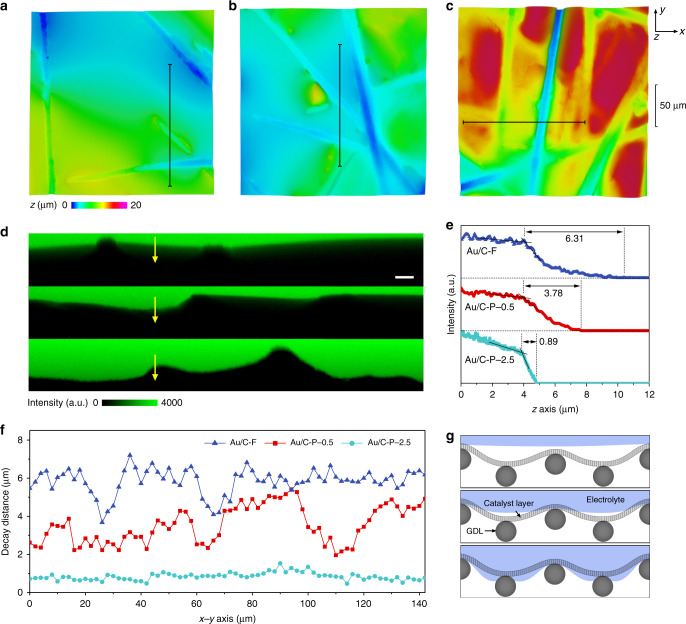
Characterization of gas–liquid–solid interfaces. **a**–**c** Confocal 3D reconstruction images of Au/C-F, Au/C-P-0.5 and Au/C-P-2.5, respectively. **d** Cross-sectional fluorescence images scanned from labelled regions (black lines) in (**a**–**c**), scale bar: 10 μm. From top to bottom are Au/C-F, Au/C-P-0.5 and Au/C-P-2.5, respectively. **e** Corresponding *z *axis fluorescence intensity line scans of labelled regions (yellow arrows) in (**d**). **f** Statistics of fluorescence decay distance from entire area of the cross-sectional fluorescence images in (**d**). **g** Schematic illustration of interfacial structures of Au/C-F (top), Au/C-P-0.5 (middle) and Au/C-P-2.5 (bottom), respectively. Source data are provided as a Source data file.

To understand the fundamental role of interfacial wettability, the relationship between electrochemical performance and the wettability-controlled structure of TPC interfaces was further investigated. We applied confocal laser scanning microscopy (CLSM) for the first time to directly observe the structure of TPC interfaces (see Methods and Supplementary Fig. 12 for experiment details)^51^. The 3D reconstruction images of Au/C-F, Au/C-P-0.5 and Au/C-P-2.5 are shown in Fig. 3a–c. The profile roughness of the images reflects the penetration degree of electrolyte into the catalyst layer along the *z* axis. Intuitively, the results indicate that the electrolyte penetration depth increased with surface hydrophilicity of the electrode. For example, Au/C-F provided an almost flat profile, with only a small amount of electrode textual observed, indicating that the liquid phase was supported by the top of catalyst layer. Conversely, for Au/C-P-2.5, the hydrophilic nature of the catalyst layer resulted in a richly textured profile, indicating that the liquid phase penetrated deep into the electrode. In order to ascertain the actual wetting states of the three electrodes, cross-sectional images along *z* axis were selected for comparison. As shown in Fig. 3d, the cross-sectional images show the interfacial structures of Au/C-F, Au/C-P-0.5 and Au/C-P-2.5 (from top to bottom). The bright green region indicates occupancy by the luminous electrolyte phase, while the dark non-fluorescent regions indicate occupancy by either the gas phase or the solid electrode phase. To distinguish the liquid–gas interface and the liquid–solid interface, which is important to reveal the nature of the three-phase structures, fluorescence decay behavior across the interface was examined. Due to the resolution limitation of CLSM, the fluorescence intensity does not decay to zero at the boundary of the liquid phase. Instead, a trailing effect occurs, where the decay distance is highly dependent on the properties of the second phase. In our experiments, the typical decay distance over the liquid–solid interface (<1 μm) is significantly shorter than that of the liquid–gas interface (>6 μm) due to the strong light absorption and blocking effects of the solid phase (Supplementary Fig. 13). Fluorescence intensity line scans along the *z* axis from the cross-sectional images (yellow arrows) are plotted in Fig. 3e. The decay distances of Au/C-F (6.31 μm) refers to a typical liquid–gas interface. While for Au/C-P-0.5 and Au/C-P-2.5, the decay distance was reduced to 3.78 and 0.89 μm, respectively, indicating that the liquid–solid interface gradually replaced the liquid–gas interface. Figure 3f provides the statistical data of the decay distances over the entire cross-sectional area. Most part of Au/C-F showed the specific decay distance of a liquid–gas interface, demonstrating that the catalyst layer is mostly exposed to the gas phase without contact with the electrolyte. The catalyst layer is therefore a gas-solid interface dominated by the Cassie state^52^. On the contrary, liquid–solid interfaces occupied the whole region for Au/C-P-2.5, revealing a Wenzel state that the catalyst layer is immersed by the electrolyte. Meanwhile, a Cassie-Wenzel coexistence state was observed for Au/C-P-0.5, since the fluorescence decay distance fluctuated between the typical decay distance of the liquid–gas and liquid–solid interfaces. In brief, as schematically depicted in Fig. 3g, the Cassie state, Cassie-Wenzel coexistence state and Wenzel state were the dominant wetting states for Au/C-F, Au/C-P-0.5 and Au/C-P-2.5, respectively.

Beyond the variation of ECSA caused by different contact modes between catalyst and electrolyte, the interfacial structure and wettability was expected to affect the efficiency of CO_2_ transportation, leading to a fundamentally different CO_2_RR performance when operating at high current densities. Quantitative evidence of interfacial CO_2_ transportation under electrochemical nonequilibrium conditions is thus required to provide a firm foundation for rationalizing the influence of interfacial structures of electrochemical CO_2_RR performance, as is explored below.

## Discussion

**Fig. 4:**
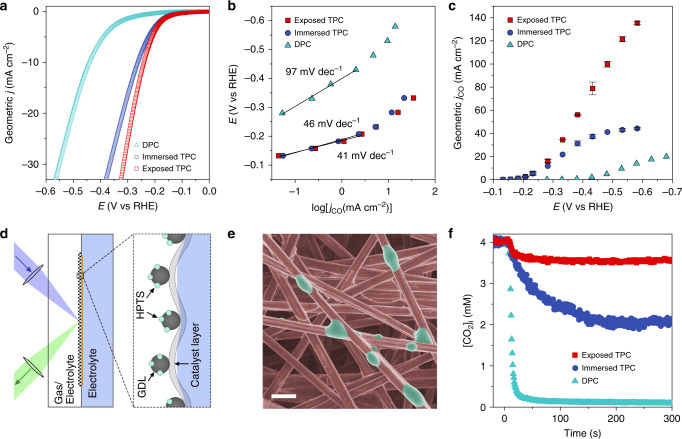
Electrochemical CO_2_RR over different interfacial structures. **a** LSV of Au/C-P-0.5 electrodes in 1 M KOH for the TPC systems and in CO_2_-saturated 1 M KHCO_3_ for the DPC system. **b** CO production Tafel plot of Au/C-P-0.5 electrodes over exposed TPC, immersed TPC and DPC systems. **c** Geometric *j*_CO_ of Au/C-P-0.5 over the three systems versus applied cathodic potentials. Error bars represent the standard deviation of three independent experiments. **d** Schematic illustration of in situ fluorescence electrochemical spectroscopy measurements. **e** Colour-modified SEM image of HPTS-labelled gas diffusion layer. Bright green is the CO_2_-sensitive HPTS gel immobilized on the surface of carbon fibres, scale bar: 20 μm. **f** Time-dependent [CO_2_]_i_ during CO_2_RR at 50 mA cm^−2^. Time = 0 s represents the start of electrolysis. Source data are provided as a Source data file.

To demonstrate the effect of CO_2_ transportation on electrochemical CO_2_RR performance, we developed two typical TPC systems, denoted here as the immersed TPC system and the exposed TPC system (the former is also known as an underwater aerophilic system)^53^, and a traditional DPC system as depicted schematically in Supplementary Fig. 14. From the LSV curves shown in Fig. 4a, the Au/C-P-0.5 electrode in both of the TPC systems exhibited similar performance below 10 mA cm^−2^. The difference in the total current density (*j*) between the two TPC systems became larger as the cathodic potential increased, implying a gradual change in the rate determining step at higher current densities. CO production Tafel plots further confirmed similar CO_2_RR electrochemical kinetics for the two TPC systems at low current densities (Fig. 4b), achieving Tafel slopes of 41 and 46 mV dec^−1^ for exposed and immersed TPC systems, respectively. In contrast, the DPC system showed a −0.2 V shift in the onset potential for CO_2_RR relative to the TPC systems with a Tafel slope of 97 mV dec^−1^, which can be ascribed to the limited electron transfer rate in the neutral-pH carbonate electrolyte^23^.

The electrochemical CO_2_ reduction products were subsequently examined at fixed potentials. As shown in Fig. 4c, the *j*_CO_ of Au/C-P-0.5 in the exposed TPC system increased quickly to 99.9 ± 2.5 mA cm^−2^ at −0.48 V, achieving a steady CO Faradaic efficiency of 94.3 ± 2.5% from −0.2 to −0.5 V (Supplementary Fig. 15). The immersed TPC system showed similar CO_2_RR activity to the exposed TPC system below −0.2 V. But the *j*_CO_ gradually stopped growing at more negative potentials (maximized at ~45 mA cm^−2^), accompanied by a dramatically drop in CO Faradaic efficiency to 81.7% at −0.48 V. For the DPC system, the maximum *j*_CO_ was observed around 23 mA cm^−2^. It is worth noting that the current density of H_2_ evolution is similar between the exposed and immersed TPC systems, implying the different CO_2_RR activity is mainly due to CO_2_ transport effects rather than a surface area effect. We therefore hypothesize that at a critical current density threshold, CO_2_ transportation became the rate determining step in CO_2_RR, with the threshold being highly dependent on the interfacial structure of the electrode used.

Interfacial mass transfer is one of the key factors to consider when evaluating CO_2_RR at large current densities (i.e. conditions relevant for industrial electroreduction of CO_2_)^27^. Although distinct electrochemical performance has been observed, the fundamental CO_2_ transportation behaviour at different interfaces remains largely unexplored. Due to the electrochemical conversion of CO_2_ on the surface of catalysts, the interfacial CO_2_ concentration ([CO_2_]_i_) should be lower than that in the bulk gas phase ([CO_2_]_0_). For TPC systems, the interfacial CO_2_ mass-transfer flux *N* is a function of mass-transfer coefficient *k* and the difference in concentration between [CO_2_]_0_ and [CO_2_]_i_, as described by the following equation^16^:

1 $$N = k\left( {\left[ {{\mathrm{CO}}_2} \right]_0 - \left[ {{\mathrm{CO}}_2} \right]_{\mathrm{i}}} \right) \approx j_{{\mathrm{CO}}}/nF$$

[CO_2_]_0_ is an external variable that can be adjusted though pressure control^54^, whereas *k* is closely linked to the interfacial structure of an electrode, *n* is the electron transfer number of electrode reaction for each CO_2_ molecule (=2 for CO production), and *F* is the Faraday constant. Both the interfacial effective velocity and diffusivity of CO_2_ under electrochemical CO_2_RR are key factors that can influence the value of *k* (see Supplementary Methods). Because the interfacial region is thin in TPC systems, the CO_2_ mass-transfer flux *N* in the gas phase will be equal to that in the liquid^16^. It means that *N* is proportional to the current density of CO_2_RR (i.e., *j*_CO_) when non-Faradaic consumption of CO_2_ is neglected. Thus, [CO_2_]_i_ is the only parameter needed to quantify *k* and examine the interfacial CO_2_ transportation behaviour. However, traditional characterization methods are not capable of tracking the local CO_2_ concentration at the microscale, especially under electrochemical conditions. Fluorescence probes show very high sensitivity to trace amounts of CO_2_, high spatial resolution, fast time responses and specific CO_2_ molecular target recognition in complex environments, though have never previously been used to study electrochemical systems for CO_2_RR^55–58^. To this end, we developed here the technique of in situ fluorescence electrochemical spectroscopy (FES) to monitor the [CO_2_]_i_ during CO_2_RR.

8-hydroxypyrene-1,3,6-trisulfonic acid (HPTS) is a typical fluorescence probe used to detect local CO_2_ concentrations, the basic detection principle of which is described in the Supplementary Methods. A HPTS-based probe was deposited onto the reverse side of the GDL (Fig. 4d, e). We then fixed the HPTS-labelled Au/C working electrode into an electrochemical cell equipped with light pass channels for excitation and emission of light (Supplementary Fig. 16). The detected CO_2_ concentration can be approximated to the [CO_2_]_i_ according to the Fick’s first law (see Methods and Supplementary Methods for details). Considering the detection limit of HPTS and the relatively low CO_2_ solubility in electrolyte due to the salting out effect^59^, the initial [CO_2_]_0_ was fixed at 4.1 mM for all systems using CO_2_/Ar mixtures as the gas source (see Supplementary Methods). As shown in Fig. 4f, under 50 mA cm^−2^ chronopotentiometry the measured [CO_2_]_i_ for all the systems decreased at the beginning of electrolysis, achieving a new steady state after a certain period of electrolysis. The exposed TPC system showed the smallest decrease in [CO_2_]_i_ (going from 4.1 to 3.5 mM), becoming stable within 50 s of electrolysis, while for the immersed TPC system the [CO_2_]_i_ decreased continuously and eventually stabilized at 2.0 mM after 200 s electrolysis. The DPC system showed the largest decrease in [CO_2_]_i_ during electrolysis, with an equilibrium [CO_2_]_i_ of only 0.1 mM. Mass-transfer coefficients *k* were calculated to be 0.27, 0.07 and 0.02 cm s^−1^ for exposed TPC, immersed TPC and DPC systems, respectively. For the DPC system, *k* was very low owing to the poor CO_2_ diffusivity in the liquid phase. For the two TPC systems, which shared the same diffusivity, the difference in *k* mainly originated from the different effective CO_2_ flow velocities over the porous GDL. As such, a comprehensive analysis of the relationship between the cathodic reactions and [CO_2_]_i_ is clearly required to correlate the very fast CO_2_ transportation in the exposed TPC system to its superior CO_2_RR performance.

**Fig. 5:**
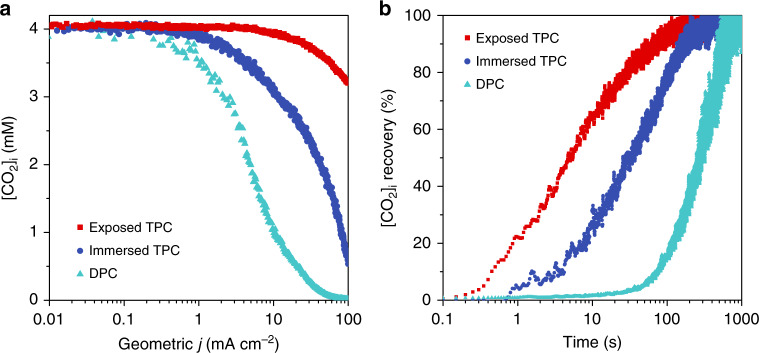
Effect of CO_2_ transportation on electrochemical CO_2_RR. **a** [CO_2_]_i_ versus geometric current density showing the best CO_2_ concentration stability is achieved in the exposed TPC system, especially at high current densities. **b** Time-dependent [CO_2_]_i_ recovery after stopping electrolysis. Source data are provided as a Source data file.

The electrochemical reaction rate can be easily controlled through potential regulation. We therefore performed LSV at slow scan rates (2 mV s^−1^) to monitor the [CO_2_]_i_ variation for all systems (Supplementary Fig. 17). We assumed that a slow scan rate would create a quasi-equilibrium state for small potential windows, allowing the relationship between [CO_2_]_i_, cathodic potential and the corresponding current density to be revealed. As seen in Fig. 5a, all the systems showed negligible change in the [CO_2_]_i_ at low current densities. However, a 50% [CO_2_]_i_ reduction was found for the DPC and immersed TPC systems at 5 and 40 mA cm^−2^, respectively. In contrast, the [CO_2_]_i_ of the exposed TPC system decreased very slowly along with the increased cathodic potential, retaining 80% of its initial concentration even at 100 mA cm^−2^. These trends closely matched those seen in the electrochemical CO_2_RR performance experiments (Fig. 4a–c), confirming that CO_2_ transportation dominates the reaction kinetics at high current densities.

In order to probe the interfacial CO_2_ transportation behaviour independently from Faradaic processes, we further examined the time-dependent [CO_2_]_i_ recovery after stopping electrochemical reaction. After the reaction is halted, the [CO_2_]_i_ will recover to the initial equilibrium state value. As seen in Fig. 5b, the exposed TPC system showed a fast recovery rate, with over 50% of the [CO_2_]_i_ lost during electrolysis being re-supplied from bulk gas phase within 5 s. The same recovery required 32 and 280 s for the immersed TPC and DPC systems, respectively. Thus, we can conclude that effective CO_2_ transportation from the bulk phase to the optimized three-phase interfaces is a key factor for the stabilization of nonequilibrium [CO_2_]_i_, thus achieving non-diffusion controlled CO_2_RR performance at high current densities.

In summary, a typical metal/C electrode comprising an Au/C NPs film immobilized on the gas diffusion layer were successfully prepared and evaluated for CO_2_RR. Confocal laser scanning microscopy provided valuable information for the interface structures of wettability-controlled three-phase interfaces. The quantification of interfacial CO_2_ transportation behaviour though in situ fluorescence electrochemical spectroscopy further revealed the fundamental role of interfacial CO_2_ transportation in determining the stability of CO_2_ equilibrium concentration during electrochemical reaction. The TPC system with Cassie-Wenzel coexistence wetting state was identified as the ideal interface structure for CO_2_RR, evidenced by an interfacial CO_2_ mass-transfer coefficient of 0.27 cm s^−1^ which was sufficient to maintain 80% of the initial CO_2_ concentration at the interface when operating at current densities above 100 mA cm^−2^. Our results thus provide valuable new insights for the rational design of TPC systems for non-diffusion controlled CO_2_RR, as well as TPC systems for other electrochemical processes that use gaseous reactants.

## Methods

### Characterization

Transmission electron microscopy (TEM) images were obtained on a JEOL-2100 microscope or a JEOL2100F microscope, with both instruments operated at an accelerating voltage of 200 kV. Field emission scanning electron microscopy (FESEM) images were obtained on a Hitachi S-4800 instrument. Fourier-transform infrared (FTIR) spectra were collected on Excalibur 3100 (Varian, USA) spectrophotometer using an attenuated total reflection mode over the range 4000–600 cm^−1^ at a resolution of 4 cm^−1^. X-ray photoelectron spectroscopic (XPS) data were obtained on a Quantum 2000 Scanning ESCA Microprobe (Physical Electronics) using monochromatic Al-Kα radiation (*hν* = 1486.6 eV) as the excitation source. X-ray absorption fine structure (XAFS) data were collected at the Beijing Synchrotron Radiation Facility (BSRF), with the raw fluorescence mode data processed via background-subtraction, normalization and Fourier transformations using the standard procedures within the ATHENA program. Fluorescence spectra were recorded on F-4600 (Hitachi, Japan) luminescence spectrometer.

### Au/C electrode preparation

Au NPs with diameters of 8.95 ± 0.52 nm were prepared using a seed growth method^60^. Next, 1 mL of a dispersion of the Au NPs in cyclohexane (8 mg mL^−1^) was added to 40 mL of a dispersion of carbon black (VULCAN XC-72, Cabot, USA) in cyclohexane (1 mg mL^−1^), followed by ultrasonication for 30 min. The resulting dispersion was then heated to dryness under an infrared lamp. The resulting as-prepared Au/C NPs were then calcined at 200 °C for 12 h to remove surface organic ligands on the Au NPs. The Au weight ratio in Au/C NPs product was determined to be 16.1 wt.% by thermogravimetric analysis.

### Working electrode preparation

The preparation and surface modification steps are schematically shown in Supplementary Fig. 18. Commercial carbon fibre papers (TGP-H-060, Toray, Japan) were modified with 1% poly(tetrafluoroethylene) (PTFE) and used as a gas diffusion layer (GDL). A catalyst ink (1 mg mL^−1^) comprising Au/C NPs dispersed in a water-ethylene glycol-n-propyl alcohol mixed solution (volume ratio 1:1:1) was then drop cast onto the GDL to achieve a loading of 120 μg_Au_ cm^−2^. For the surface hydrophobicity modification, the as-prepared Au/C electrode was treated with fluorine-terminated silane coupling agent (1,1,2,2-perfluorodecyltrimethoxysilane). Briefly, the Au/C electrode was placed into a stainless steel vessel containing 10 μL of coupling agent. Then, the vessel was evacuated and maintained at 90 °C for 10 min, producing the sample known as Au/C-F. Surface hydrophilicity modifications involved subjecting Au/C electrodes to air plasma (PDC-002, Harrick Plasma, USA) treatment for different times were then performed (the resulting electrodes are labelled herein as Au/C-P-*x*, where *x* represents the plasma treatment time in min). The chamber is evacuated for 2 min, after which plasma treatment (low level) was applied.

### Electrochemical measurements

The electrochemical CO_2_RR experiments were performed in a gas-phase-connected H-type electrochemical flow cell, with data collected using a CHI660E electrochemical workstation (Shanghai Chenhua, China). The diameter and width of the channels in the flow cell are 20 and 12 mm, respectively. The electrode potentials after *i*R compensation were rescaled to the reversible hydrogen electrode (RHE) using the following equation:

2 $$E_{{\mathrm{RHE}}} = E_{{\mathrm{Ag}}/{\mathrm{AgCl}}} + 0.1976 + 0.0591 \times {\mathrm{pH}}$$

For cathodic CO_2_RR tests, platinum and Ag/AgCl (saturated KCl) were used as counter electrode and reference electrode, respectively. 1 M KOH was were used as electrolytes (pH = 12.42, estimated from surface pH simulation by Dinh^29^) for three-phase electrochemical systems, While, for double phase electrochemical system, CO_2_ saturated 1 M KHCO_3_ was used as electrolyte (pH = 7.96). The gas flow rate was controlled at 20 sccm. The catholyte and anolyte flow rates were controlled at 5 mL min^−1^ via a peristaltic pump. The cathode and anode were separated via a proton exchange membrane (N-117, Dupont, USA). The length of all chronoamperometry experiments were fixed at 30 min before the gas products were collected for analysis. Gas-phase products were quantified using a gas chromatograph (GC-2014C, Shimadzu, Japan) equipped with thermal conductivity detector (Supplementary Fig. 19). The CO geometric partial current density *j*_CO_ was calculated using the following formula:

3 $$j_{{\mathrm{CO}}} = \frac{{nmFV}}{S}$$

In which *n* is the number of electrons transferred, *F* is Faraday’s constant, *m* is the mole fraction of CO quantified by gas chromatography, *V* is the total molar flow rate in the gas phase (mol s^−1^), *S* is the geometric area of the electrode. The Faradaic efficiency, FE, to CO was calculated using the following formula:

4 $${\mathrm{FE}} = \frac{{j_{{\mathrm{CO}}}}}{{j_{{\mathrm{tot}}}}} \times 100{\mathrm{\% }}$$

where *j*_tot_ is the total current density. The cell energy efficiency, EE, for CO_2_RR was calculated using the following formula:

5 $${\mathrm{EE}} = \frac{{1.34 \times {\mathrm{FE}}}}{{{\mathrm{Cell}}\;{\mathrm{voltage}}}}$$

The cathodic energy efficiency was calculated for the cathodic half-cell, where the overpotential of the oxygen evolution reaction is assumed as zero.

### Contact angle measurements

The contact angles were measured using a contact angle system (OCA 20, Dataphysics, Germany) at ambient temperature, with the probe liquid being 2 μL of water. All contact angle images were taken 5 s after the application of the liquid droplet on the surface of samples. The average contact angle was obtained by measuring three different positions of the same sample.

### Fluorescent probe molecule preparation

8-hydroxypyrene-1,3,6-trisulfonic acid (HPTS) shows a characteristic emission peak at 520 nm in the presence of CO_2_, the intensity of which shows a strong CO_2_ concentration dependence^56^. HPTS was used as a pH-sensitive fluorescence dye here for the determination of interfacial CO_2_ concentrations. Briefly, a methanolic solution of tetraoctylammonium hydroxide (TH, 0.5 M) was prepared from a methanolic solution of tetraoctylammonium bromide (0.5 M), with silver oxide being used to facilitate the anion exchange. Next, 3.8 mg of HPTS was added to 1 mL of methanolic 0.5 M TH solution, followed by the addition of a further 2.5 mL of methanol to form the fluorescent probe solution. Then, 30 μL of the probe solution was deposited on the reverse side of gas diffusion layer (i.e. the opposite side to the Au/C NPs film) and dried under an infrared lamp for about 5 min. A hydrophobic silane-based sol–gel was then prepared and dabbed onto the surface of fluorescent probe to prevent leaching of HPTS when in contact with electrolyte^56^.

### Fluorescence electrochemical spectroscopy (FES)

The in situ FES measurements utilized an electrochemical workstation (CHI660E, Shanghai Chenhua, China) integrated with a fluorescence spectrophotometer (F-4600, Hitachi, Japan). A homemade electrochemical cell equipped with light input/output channels and a gas flow system was used. Excitation and emission wavelengths for HPTS were 485 and 520 nm, respectively. For standard curve measurements, a time scan PL intensity curve was collected without electrolysis (Supplementary Fig. 20). During the measurement, the CO_2_ concentration in gas phase was switched from 0 to 10% in 1% increments using Ar as the carrier gas (while maintaining a total gas flow rate of 50 mL min^−1^). A fluorescence intensity versus CO_2_ concentration standard curve was fitted using an exponential decay equation. The standard curve was calibrated against the PL intensity of pure Ar gas before each measurement. For three-phase contact (TPC) systems, 1 M KOH was used as electrolyte and a 10.0% CO_2_ in Ar gas mixture used (equal to 4.1 mM CO_2_ concentration). For the double phase system (DPC system), the electrolyte was 1 M KHCO_3_ and a 17.8% CO_2_/Ar gas mixture used in order to achieve the same initial local CO_2_ concentration as in the TPC system (see Supplementary Methods). In order to minimize the interference of convective bulk flow, thereby allowing the intrinsic Faradaic current-induced CO_2_ transportation at interfaces to be studied, both the gas side and liquid side of the cell were was sealed without external flow before the measurements.

### Confocal laser scanning microscopy (CLSM)

Owing to the high resolution and fast response of CLSM, we can directly image the structures of the gas–liquid–solid three-phase interfaces in the TPC systems through the analysis of a series of CLSM images at different depths within the catalyst layer. All measurements were carried out on an N-C2-SIM (Nikon, Japan) CLSM. In detail, 100 μL of fluorescein-labelled 1 M KOH was deposited into a confocal dish, followed by placing a 8 × 8 mm Au/C electrode (Au/C NPs side down) onto the liquid droplet, as is shown in Supplementary Fig. 12. A 405-nm laser was then used as the excitation source, with the confocal microscope being equipped with a ×60 (water) objective lens (numerical aperture = 1.27, pinhole = 0.5, airy units).

## Supplementary information

Supplementary Information

## Data Availability

The source data underlying Figs. 1a-c, 2a-c, 3a-f, 4a–c,e,f and 5a,b and Supplementary Figs. 1-13, 15-17, 19 and 20 are provided as a Source data file. Source data are provided with this paper.

## Source data

Source Data
