# Supplementary material for: Efficient wettability-controlled electroreduction of CO2 to CO at Au/C interfaces
Source: Nat Commun. 2020 Jun 15;11:3028. doi: 10.1038/s41467-020-16847-9 (PMC7295780; doi:10.1038/s41467-020-16847-9)
Supplement: Supplementary file 1 — Supplementary Information [file 41467_2020_16847_MOESM1_ESM.pdf]

Supplementary Information

Efficient wettability-controlled electroreduction of CO_2 to CO at Au/C interfaces

Shi et al.

Efficient wettability-controlled electroreduction of CO_2 to CO at Au/C interfaces

Run Shi[1], Jiahao Guo[1,2], Xuerui Zhang[1,2], Geoffrey I. N. Waterhouse[4], Zhaojun Han[5], Yunxuan Zhao[1,2], Lu Shang[1], Chao Zhou[1], Lei Jiang[3] and Tierui Zhang[1,2]*

[1]Key Laboratory of Photochemical Conversion and Optoelectronic Materials, Technical Institute of Physics and Chemistry, Chinese Academy of Sciences, Beijing 100190, China.
[2]Center of Materials Science and Optoelectronics Engineering, University of Chinese Academy of Sciences, Beijing 100049, China.
[3]Key Laboratory of Bio-inspired Materials and Interfacial Science, Technical Institute of Physics and Chemistry, Chinese Academy of Sciences, Beijing 100190, China.
[4]School of Chemical Sciences, The University of Auckland, Auckland 1142, New Zealand.
[5]CSIRO Manufacturing, New South Wales 2070, Australia.

*e-mail: tierui@mail.ipc.ac.cn

Supplementary Figures

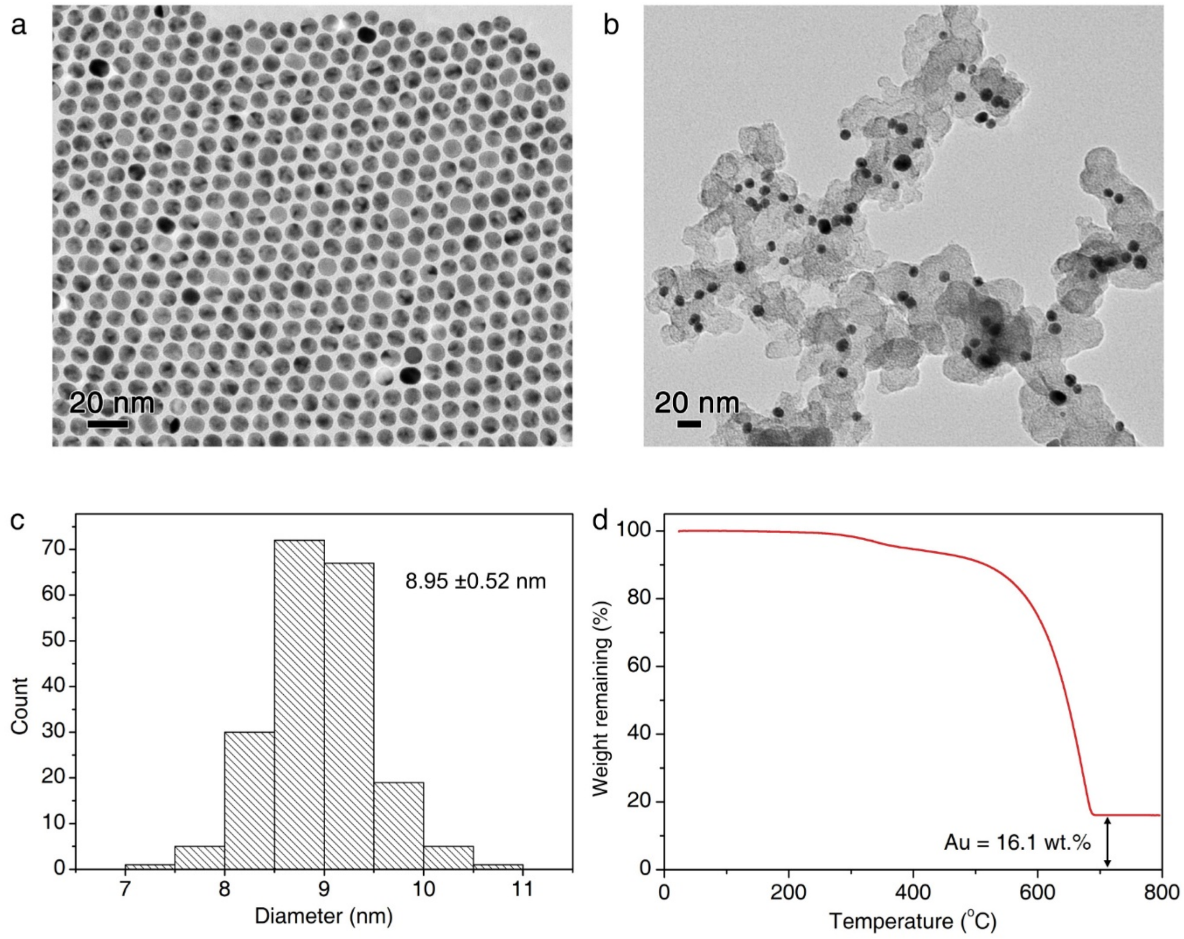

Supplementary Figure 1. Characterization of the Au/C NPs. Transmission electron microscopy (TEM) images of (a) Au NPs and (b) Au/C NPs. (c) Size distribution of the Au NPs. (d) Thermogravimetric analysis for Au/C NPs (heating rate 10 °C min^{-1} in air). Source data are provided as a Source Data file.

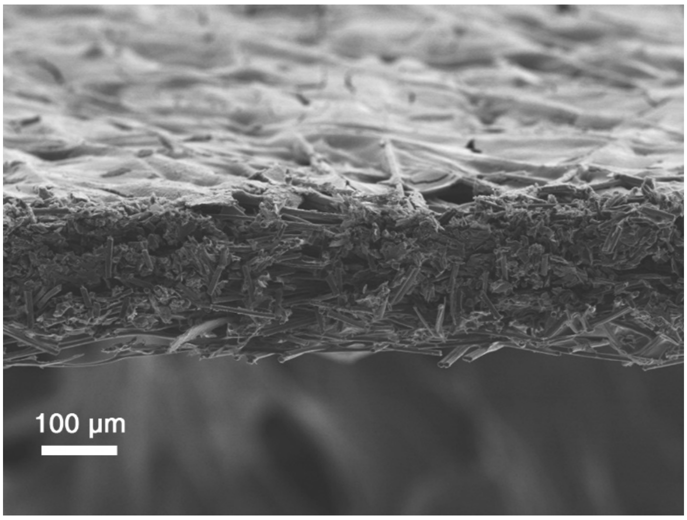

Supplementary Figure 2. Cross section SEM image of a carbon fibre gas diffusion layer loaded with Au/C NPs. Source data are provided as a Source Data file.

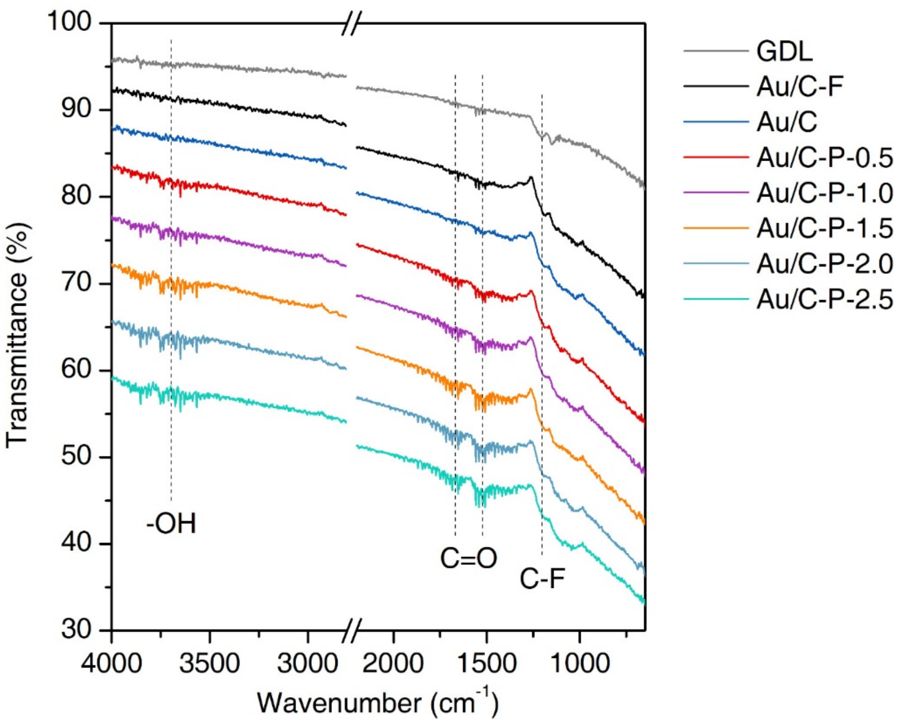

Supplementary Figure 3. FTIR spectra of GDL and the Au/C electrodes after various surface treatments. For PTFE-treated GDL, a strong C-F stretching vibration was observed at 1200 cm^{-1}. This signal dramatically decreased in intensity after the immobilization of catalyst layer. The spectrum of Au/C-F is similar to that of pristine Au/C, with a weak C-F signal being observed. For the plasma treated samples, the vibrational signals of –OH (3500-3700 cm^{-1}) and C=O (1500-1700 cm^{-1}) gradually intensified with the treatment time, indicating the formation of oxygen-containing groups such as –OH, –COOH and –CHO. The increased oxygen-containing functional groups correlate well with the decreased CA of water droplets on the electrodes. Source data are provided as a Source Data file.

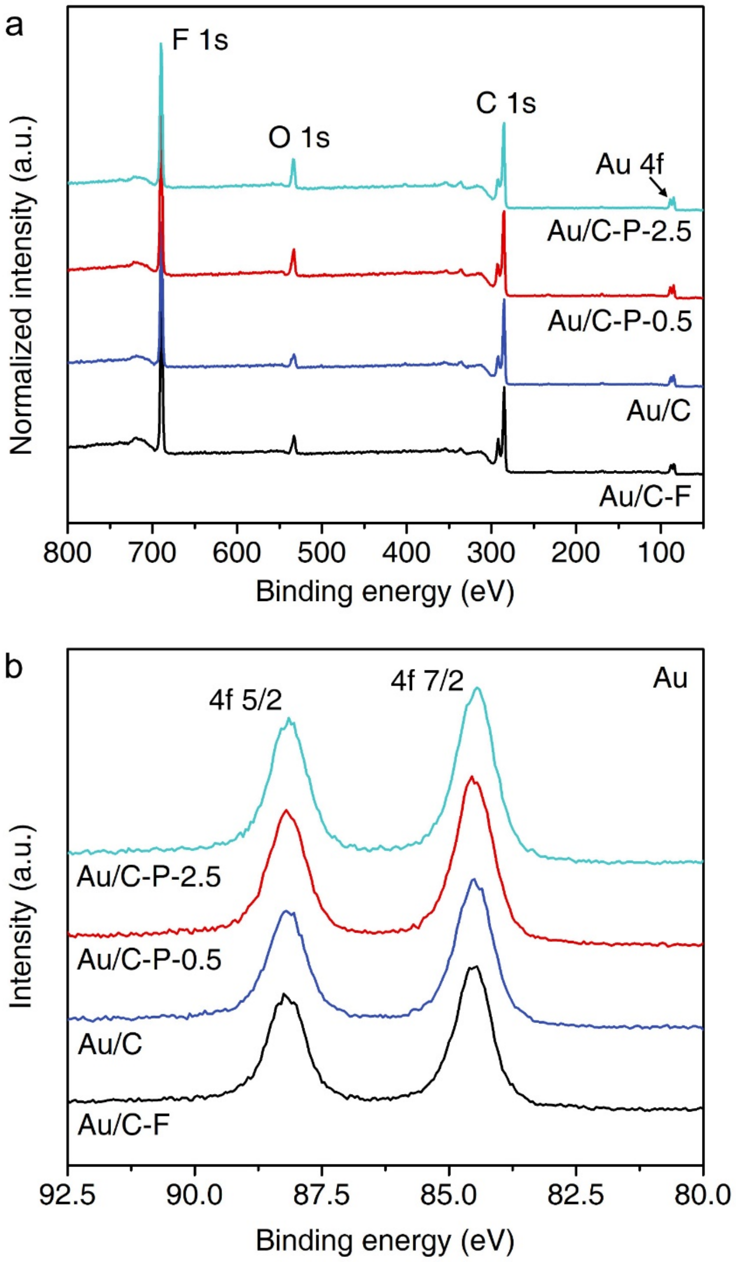

Supplementary Figure 4. XPS analysis of modified Au/C gas diffusion electrodes. (a) XPS survey spectra, and (b) Au 4f XPS spectra for Au/C electrodes with different surface modifications. Source data are provided as a Source Data file.

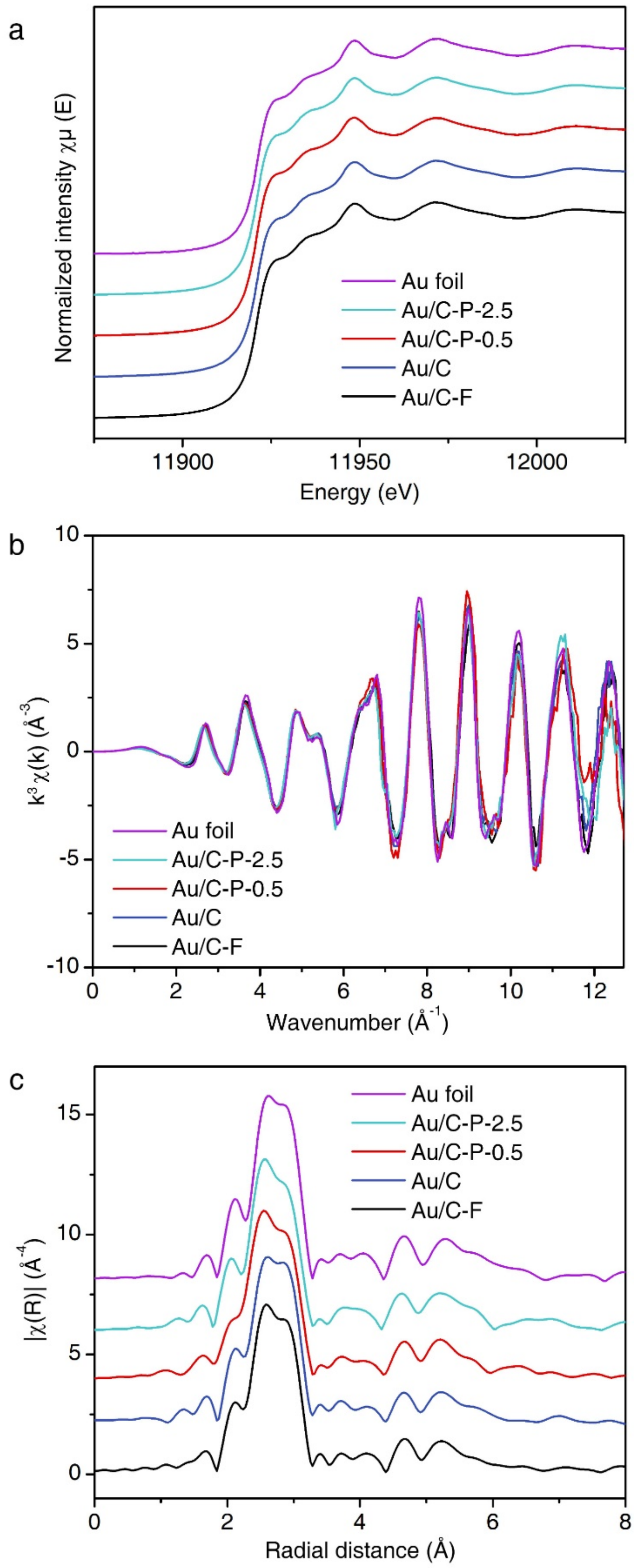

Supplementary Figure 5. XAFS analysis of modified Au/C gas diffusion electrodes. (a) Au L_3-edge XAFS spectra, (b) k^3-weighted k-space spectra, (c) Fourier transformed extended X-ray absorption fine structure spectra (in R space) for Au/C electrodes with different surface modifications. Source data are provided as a Source Data file.

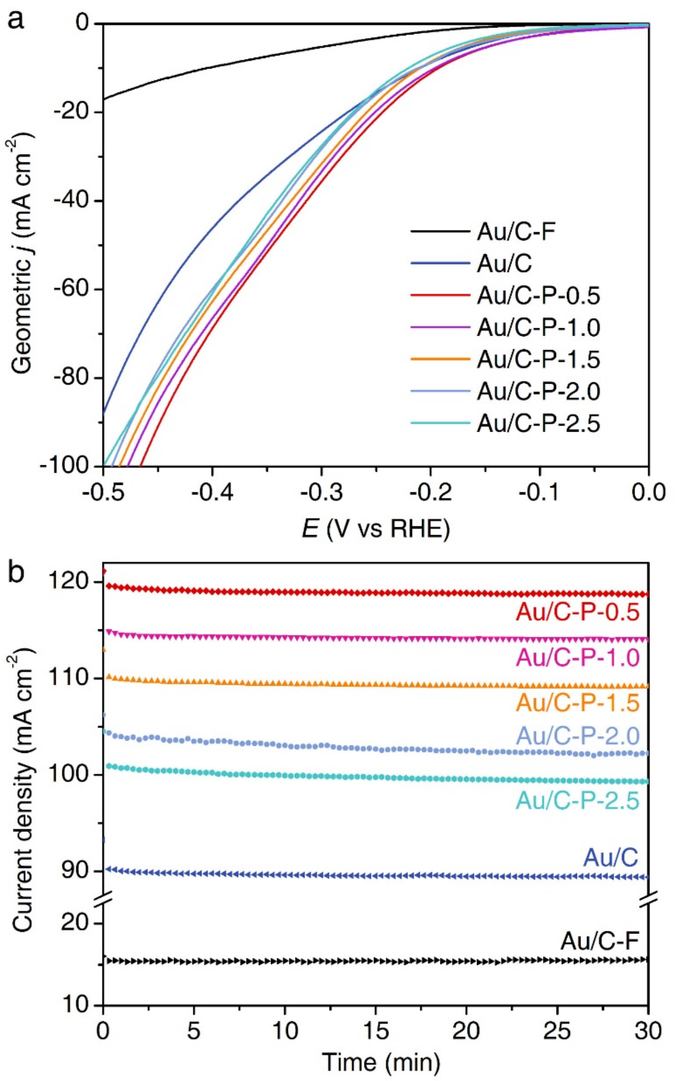

Supplementary Figure 6. Electrochemical CO_2RR tests. a) LSV curves and b) chronoamperometry tests at -0.5 V vs RHE for Au/C electrodes with various surface modifications. Source data are provided as a Source Data file.

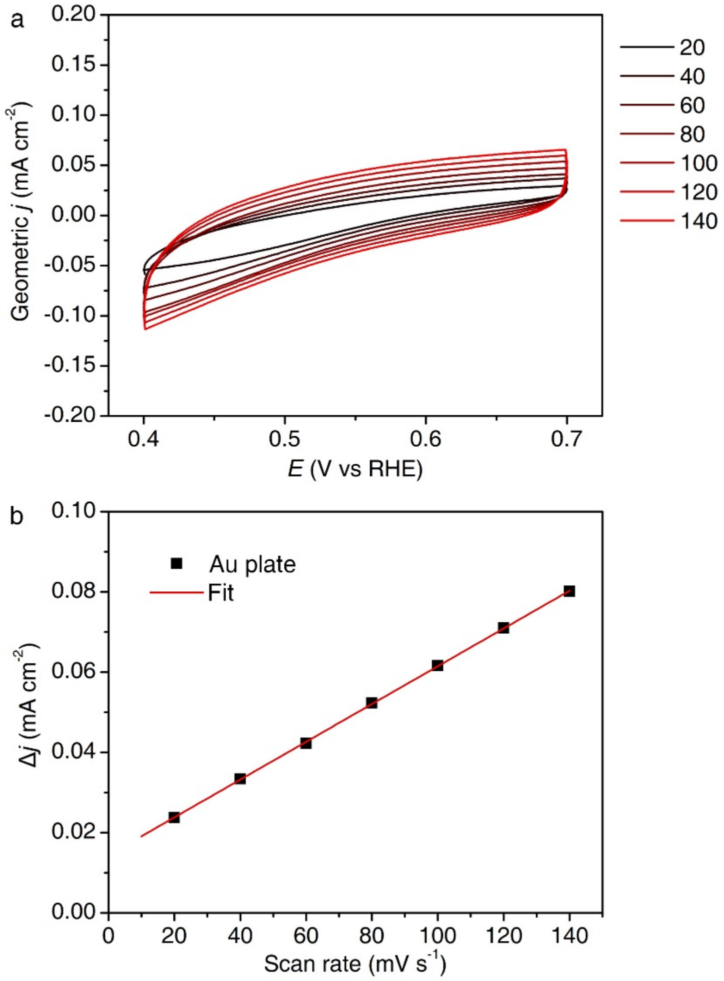

Supplementary Figure 7. ESCA measurements of Au plate reference. (a) Cyclic voltammetry curves for a gold plate electrode collected at different scan rates from 20 mV s^{-1} to 140 mV s^{-1}. (b) Charge current density differences (Δj) at 0.55 V vs RHE for the Au plate electrode plotted against scan rate. The slope of the linear line in (b) is equal to twice the double-layer capacitance C_{dl}, and thus can be used to define the electrochemical surface area (ECSA) of Au/C electrodes. Assuming that the ECSA of the Au plate electrode is equivalent to its geometric area, the ECSA of Au/C electrodes can be quantified by comparing their double-layer capacitances with the Au plate electrode. Source data are provided as a Source Data file.

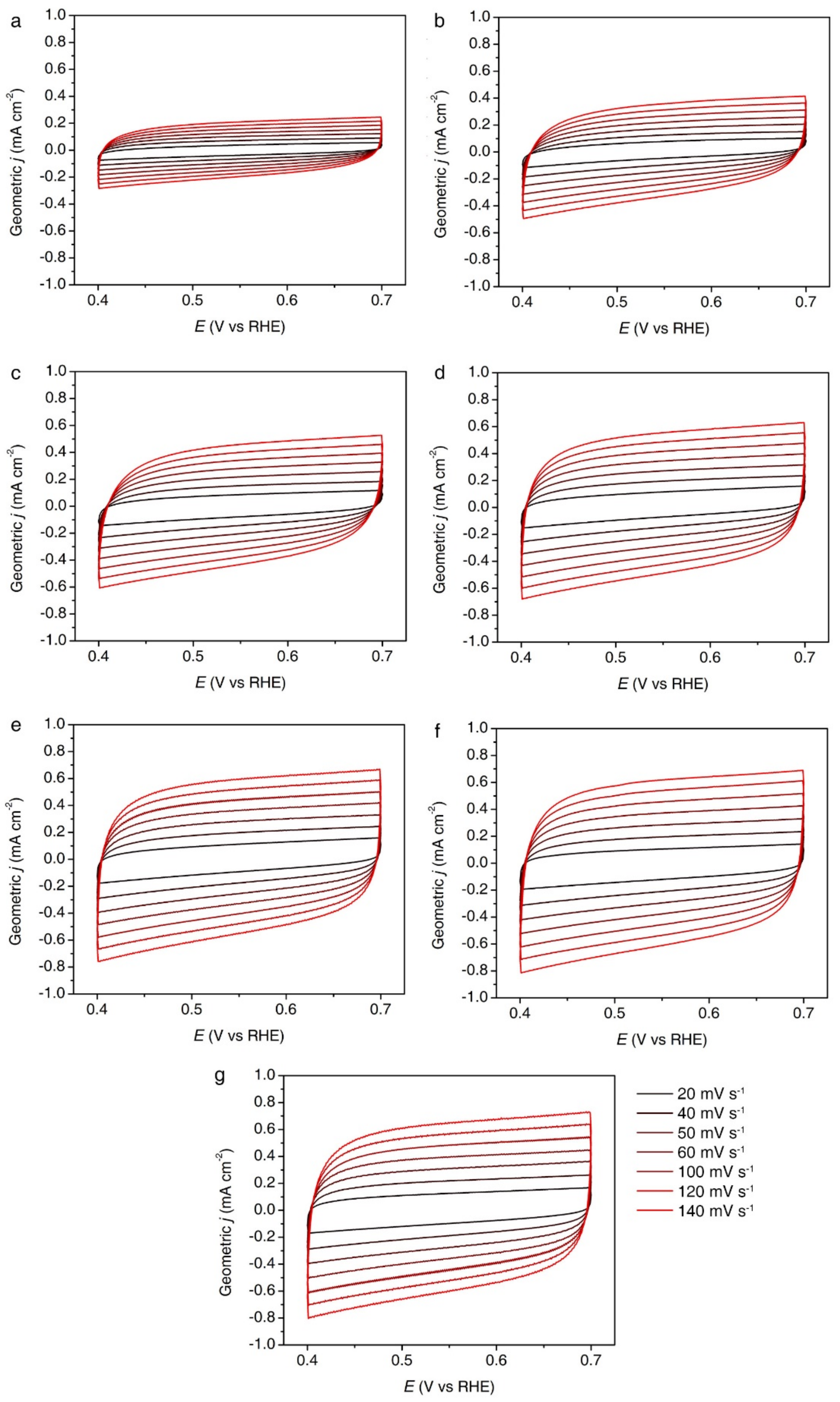

Supplementary Figure 8. ESCA measurements of Au/C electrodes. Cyclic voltammetry curves collected at different scan rates for various Au/C electrodes. (a) Au/C-F, (b) Au/C, (c) Au/C-P-0.5, (d) Au/C-P-1.0, (e) Au/C-P-1.5, (f) Au/C-P-2.0 and (g) Au/C-P-2.5. Source data are provided as a Source Data file.

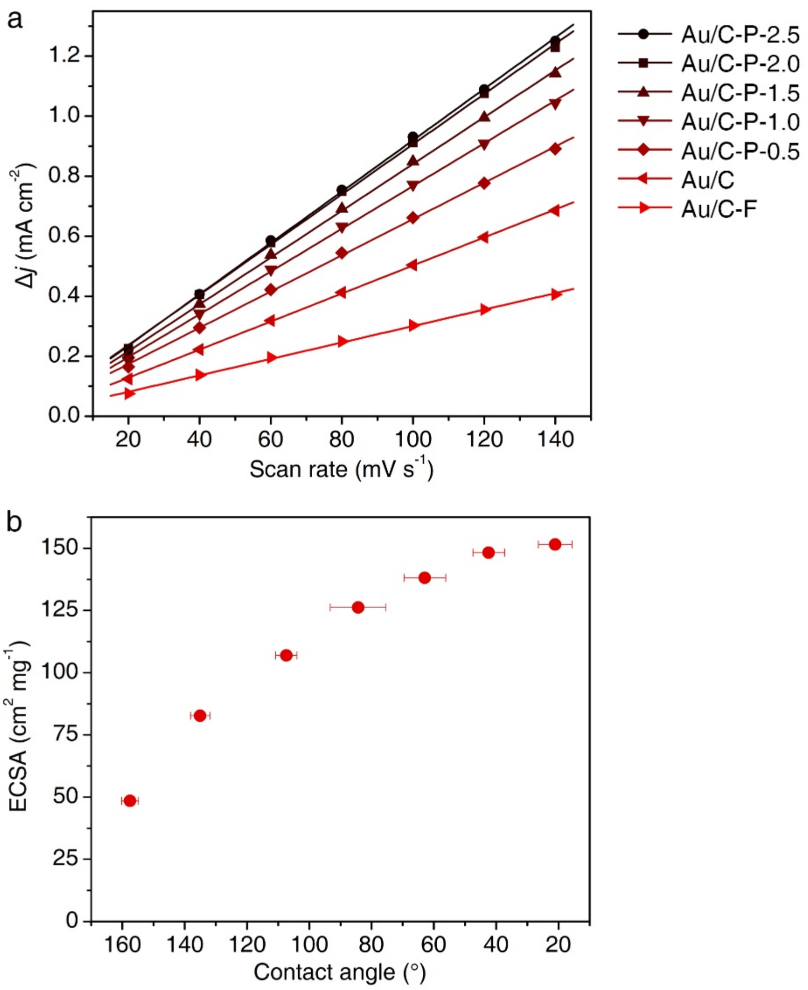

Supplementary Figure 9. ESCA measurements of Au/C electrodes. a) Charge current density differences (Δ*j*) at 0.55 V (vs RHE) for Au/C electrodes with different surface modifications plotted against scan rate. b) ECSA data for the Au/C electrodes with different wettabilities plotted against water contact angle. The numeric values of ECSA in (b) are calculated from the following equation:

$$\mathrm{ECSA} = A\ (\mathrm{cm}^2) \times \frac{C_{\mathrm{dl,t}}}{C_{\mathrm{dl,p}}} \times \frac{1}{m\ (\mathrm{mg})} \quad (1)$$

where *A* is the geometric area of the electrode, $C_{\mathrm{dl,t}}$ and $C_{\mathrm{dl,p}}$ are double-layer capacitances of tested Au/C electrodes and gold plate electrode, respectively, *m* is the loading amount of Au/C catalyst. Source data are provided as a Source Data file.

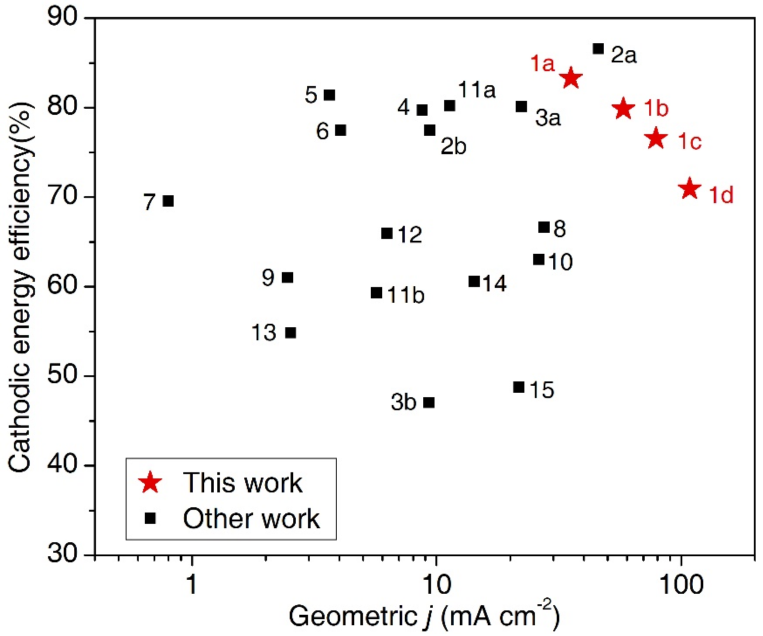

Supplementary Figure 10. Comparison of the CO_2RR cathodic energy efficiency of various Au-based electrochemical systems. Refer to Supplementary Table 1 for sample details. Source data are provided as a Source Data file.

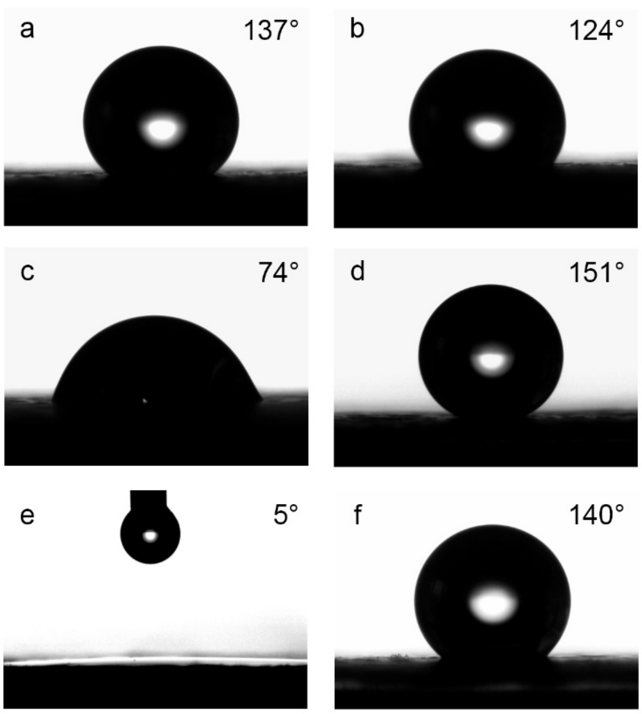

Supplementary Figure 11. Contact angle measurements of Au/C electrodes after stability tests. (a), (c) and (e) are water CAs of the front side of Au/C-F, Au/C-P-0.5 and Au/C-P-2.5 after stability tests, respectively. (b), (d) and (f) are water CAs of the reverse side of Au/C-F, Au/C-P-0.5 and Au/C-P-2.5 after stability tests, respectively. Source data are provided as a Source Data file.

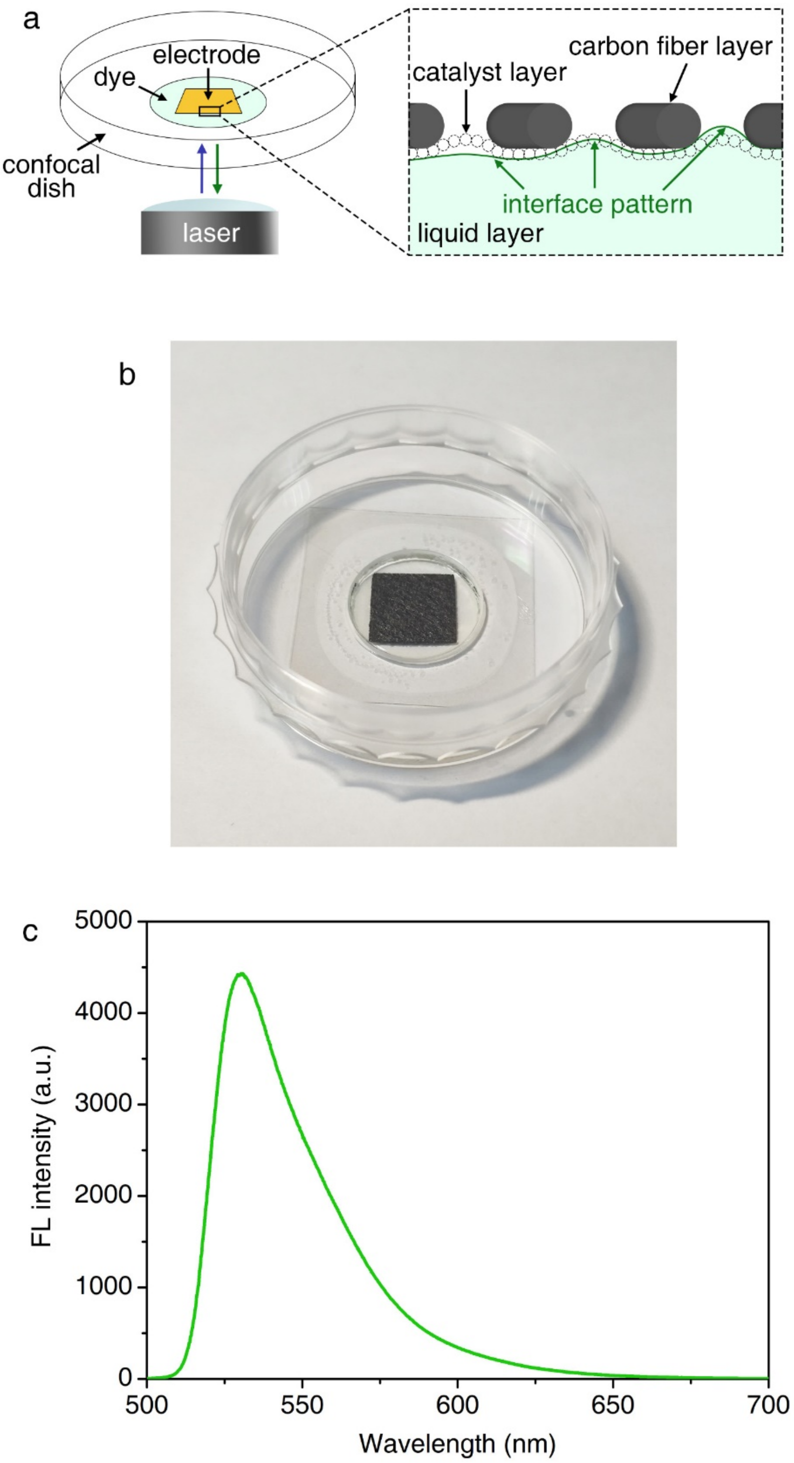

Supplementary Figure 12. CLSM experiments setup. (a) Schematic illustration of the method used to image the three phase interface by confocal laser scanning microscopy. (b) Photograph of electrode supported by the confocal dish. (c) FL spectrum of fluorescein in 1 M KOH aqueous under 405 nm excitation. Source data are provided as a Source Data file.

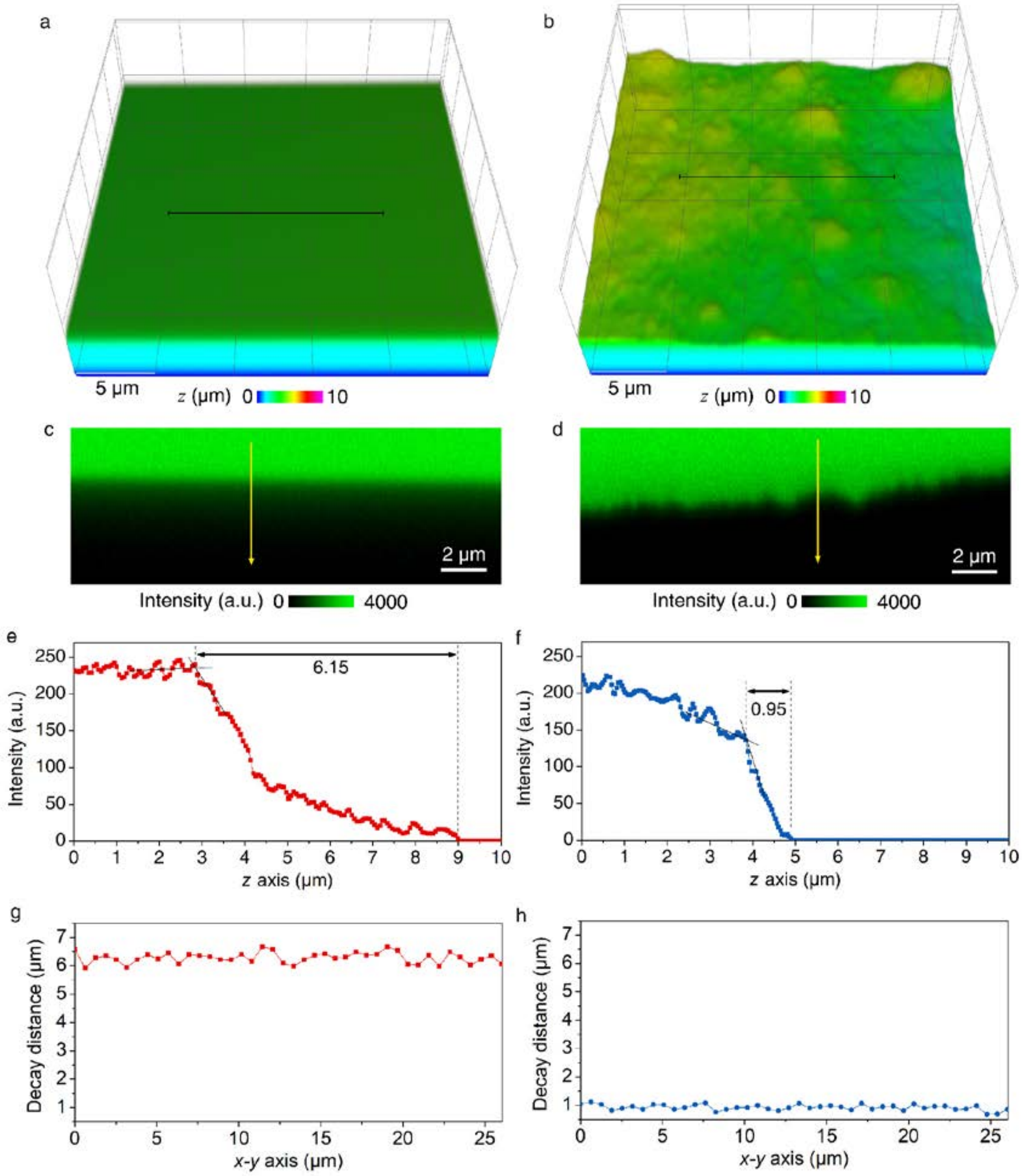

Supplementary Figure 13. CLSM experiments over reference samples. Confocal 3D reconstruction images of (a) the liquid-gas interface (liquid droplet without sample) and (b) the liquid-solid interface (liquid droplet on a hydrophilic carbon black pressed plate). (c) and (d) are cross-sectional fluorescence images from labelled regions (black lines) in (a) and (b), respectively. (e) and (f) are z axis fluorescence intensity line scans from labelled regions (yellow arrows) in (c) and (d), respectively. (g) and (h) are statistics of fluorescence decay distance from entire area of the cross-sectional fluorescence images in (c) and (d), respectively. The starting point of the decay distance is the inflection point of the fluorescence intensity, which is determined by the intersection between the tangents of the stable region and the attenuation region. The ending point is determined by the z axis where fluorescence intensity finally decayed to zero. Source data are provided as a Source Data file.

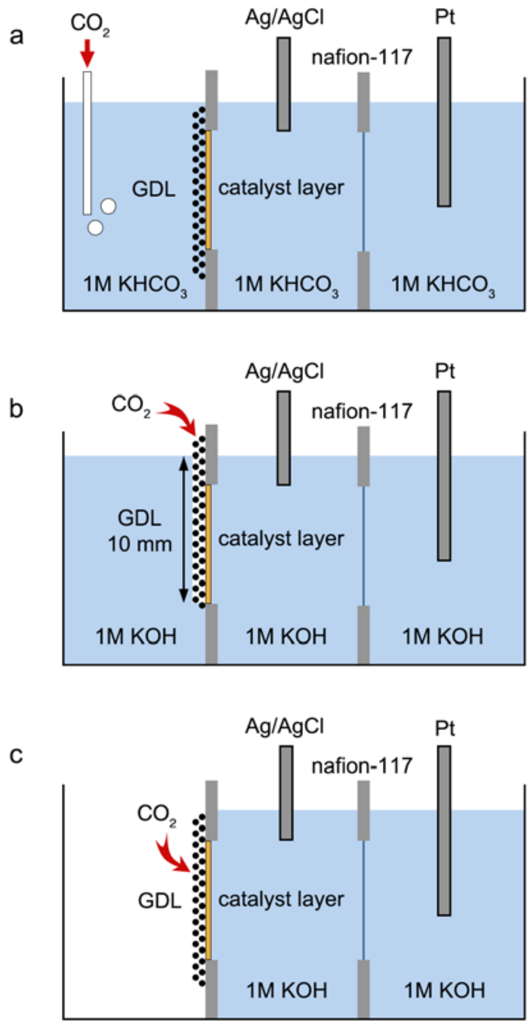

Supplementary Figure 14. Schematic illustration of the electrochemical tests setup. (a) DPC, (b) immersed TPC and (c) exposed TPC systems. For the immersed TPC system, the back side of GDL is filled with 1 M KOH. The immersion depth is controlled as 10 mm, leaving the top of hydrophobic GDL exposed to the upper gas phase. Since the hydrophobic GDL would keep gas pockets once immersed into liquid electrolyte, the gaseous CO_2 from the upper gas phase can slowly transfer to the catalyst layer through the internal pores of GDL. For the DPC system, the back side of GDL was treated with air plasma to convert the hydrophobic surface into a hydrophilic surface. Subsequently, the whole GDL is immersed into electrolyte (no direct connection between bulk gas phase and GDL). This avoided CO_2 pockets, meaning that all the CO_2 reactant reaching the catalyst layer should come from the diffusion of dissolved CO_2 in the saturated 1 M $KHCO_3$ electrolyte.

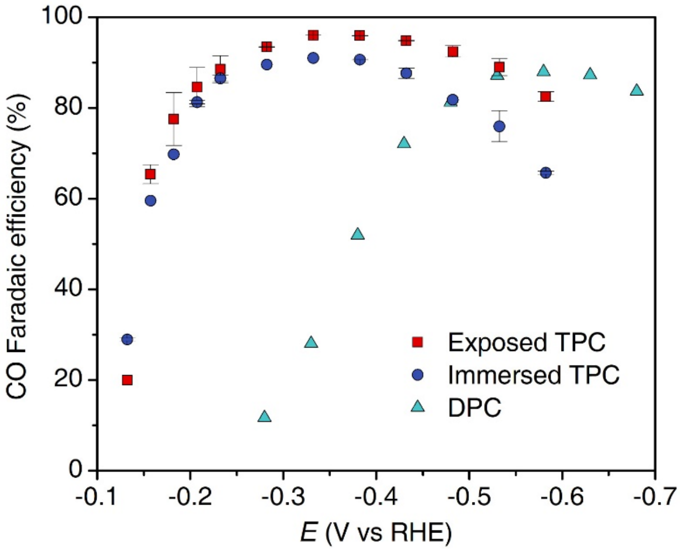

Supplementary Figure 15. CO Faradaic efficiency of Au/C-P-0.5 on exposed TPC, immersed TPC and DPC systems versus applied cathodic potentials. Error bars represent the standard deviation of three independent experiments. Source data are provided as a Source Data file.

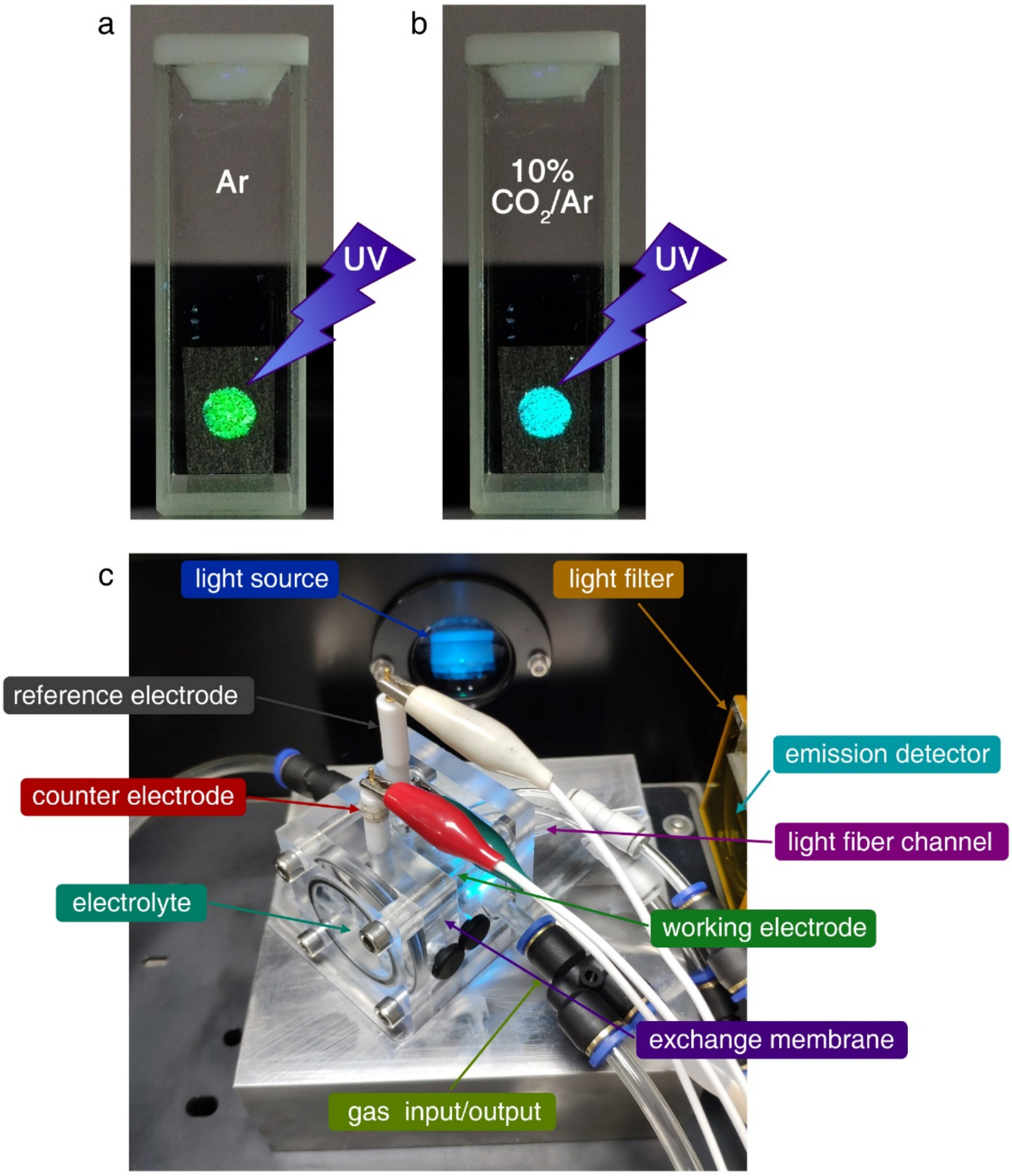

Supplementary Figure 16. Experimental setup for *in-situ* FES measurements. (a) Photograph of a working electrode loaded with the HPTS fluorescent probe under UV irradiation and exposed to Ar. (b) Photograph of a working electrode loaded with the HPTS fluorescent probe under UV irradiation and exposed to 10% CO_2/Ar. (c) Photograph of the custom-built cell used for in-situ electrochemical fluorescence spectroscopy experiments to determine local CO_2 concentrations. Source data are provided as a Source Data file.

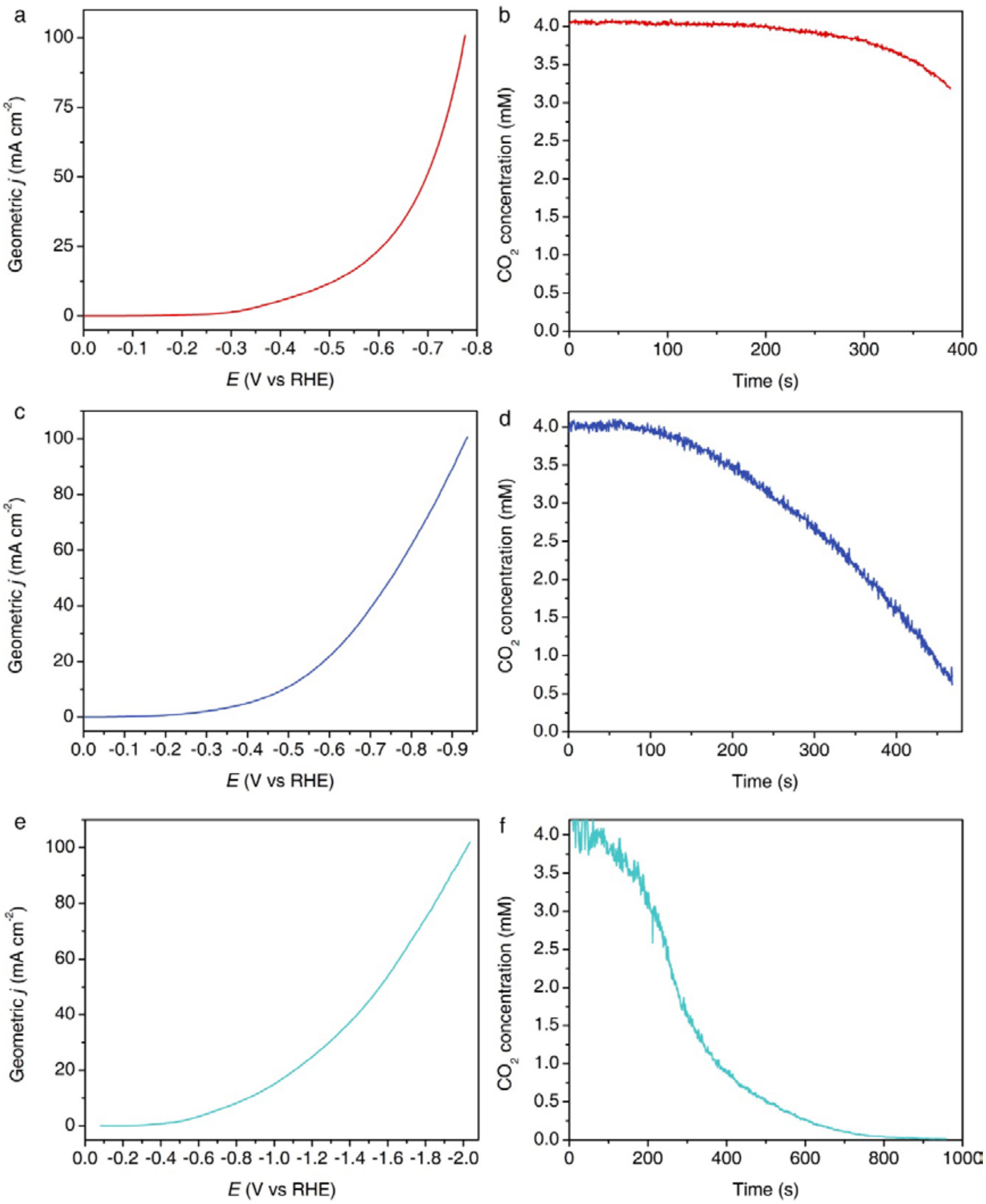

Supplementary Figure 17. *In-situ* FES measurements for the three CO_2RR systems. (a), (c) and (e) show LSV curves for the exposed three phase contact system, immersed three phase contact system and double phase contact system, respectively. (b), (d) and (f) are time-resolved local CO_2 concentration curves collected using the exposed TPC, immersed TPC and DPC systems, respectively. LSV scan rates were 2 mV s^{-1}, with 10% and 17.8% CO_2/Ar gas mixtures used for TPC system and DPC systems, respectively, to ensure a consistent initial CO_2 concentration (4.1 mM). Source data are provided as a Source Data file.

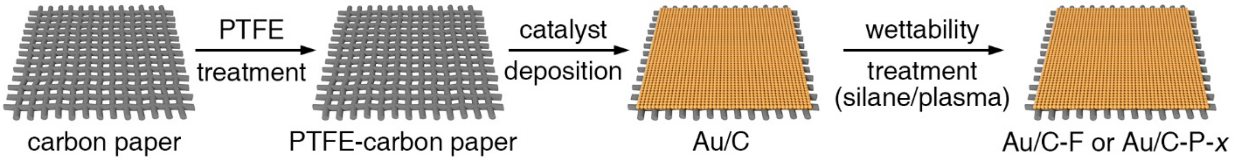

Supplementary Figure 18. Schematic illustration of the electrode preparation and surface modification processes.

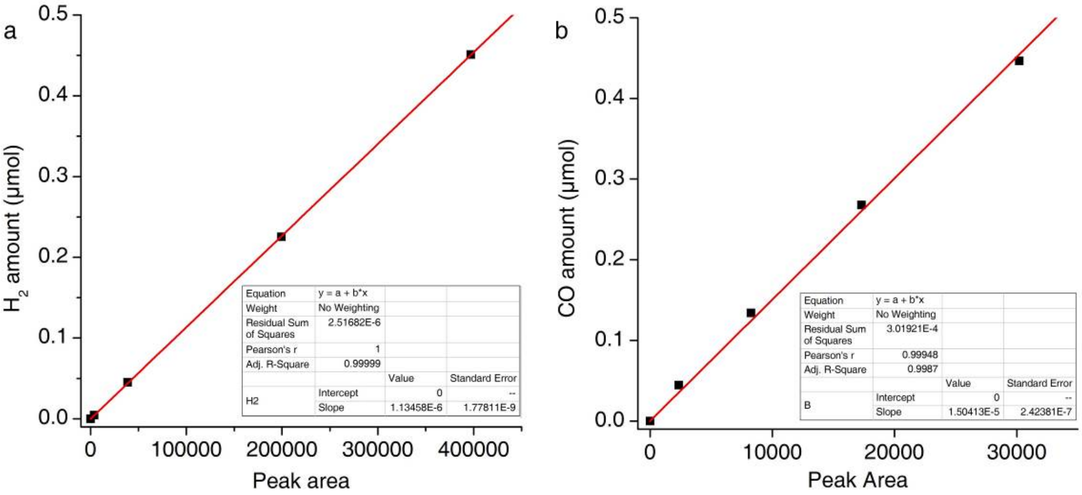

Supplementary Figure 19. Gas chromatography quantification standard curves. (a) H_2 and (b) CO. Source data are provided as a Source Data file.

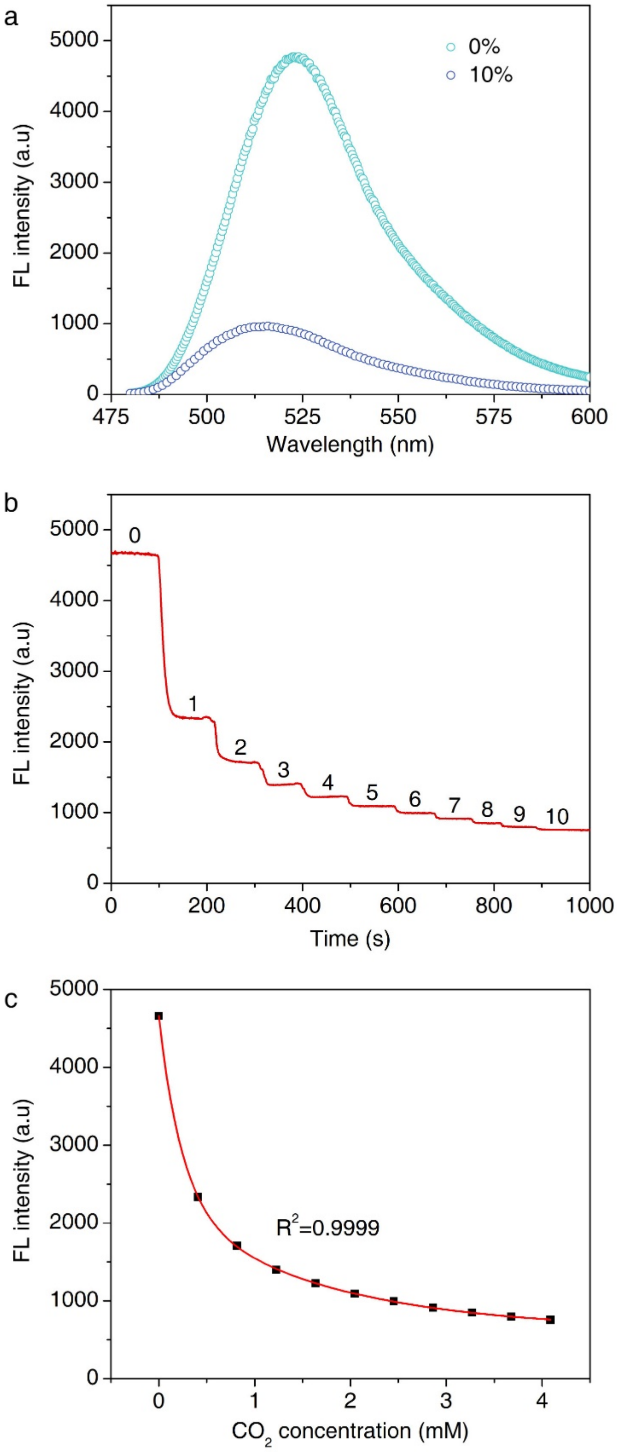

Supplementary Figure 20. Relationship between CO_2 concentration and FL intensity. (a) FL spectra collected from the TPC system purged with pure Ar (0% CO_2) and 10 CO_2/Ar gases, respectively. (b) Time scan FL spectrum obtained whilst incrementally increasing the CO_2 concentration from 0% to 10%. (c) CO_2 concentration-dependent FL intensity standard curve. The excitation wavelength was 480 nm in all experiments. Source data are provided as a Source Data file.

Supplementary Tables

Supplementary Table 1. Performance comparison of various Au-based electrochemical systems for CO_2RR.

Number	Catalyst	Electrolyte	Cathodic potential (V)	Current density (mA cm^{-2})	Cathodic efficiency (%)	Ref.
1a	Au/C-P-0.5	1 M KOH	-0.33	35.5	83.3	This work
1b	Au/C-P-0.5	1 M KOH	-0.38	58.1	79.8	This work
1c	Au/C-P-0.5	1 M KOH	-0.43	79.0	76.6	This work
1d	Au/C-P-0.5	1 M KOH	-0.48	108.4	70.9	This work
2a	MWNT/PyPBI/Au	2 M KOH	-0.29	46.0	86.6	1
2b	Au NPs	2 M KOH	-0.31	9.4	77.5	1
3a	Au needles	0.5 M $KHCO_3$	-0.35	22.2	80.1	2
3b	Au rods	0.5 M $KHCO_3$	-0.45	9.3	47.1	2
4	Au nanowires	0.5 M $KHCO_3$	-0.35	8.7	79.7	3
5	Oxide-derived Au	0.5 M $NaHCO_3$	-0.35	3.6	81.4	4
6	Au-IO film	0.1 M $KHCO_3$	-0.48	4.0	77.5	5
7	Au concave RD	0.5 M $KHCO_3$	-0.47	0.8	69.6	6
8	Bilayer Au/PE	0.5 M $KHCO_3$	-0.60	27.5	66.6	7
9	Au-Cb NP	0.1 M $KHCO_3$	-0.56	2.5	61.0	8
10	Au NP-GNR	0.5 M $KHCO_3$	-0.47	26.3	63.1	9
11a	AuFe-CSNP	0.5 M $KHCO_3$	-0.40	11.3	80.2	10
11b	Au NP	0.5 M $KHCO_3$	-0.60	5.7	59.3	10
12	Au_{25}	0.1 M $KHCO_3$	-0.70	6.3	65.9	11
13	Au(110)	0.1 M $KHCO_3$	-0.70	2.5	54.8	12
14	MWNT/PyPBI/Au	1 M KCl	-0.63	14.3	60.5	13
15	Au_{25} sphere	0.5 M $KHCO_3$	-0.67	21.7	48.7	14

Supplementary Table 2. Parameters used in the calculation of the Sechenov constant K_S.

Ion parameters	
Ion	h_{ion}
K^+	0.0922
HCO_3^-	0.0967
Dissolved CO_2 parameters	
$h_{G,0}$	-0.0172
h_T	-0.000338

Supplementary Methods

Interfacial mass transfer coefficient in three phase contact (TPC) systems. The CO_2 mass transfer coefficient at gas-liquid-solid three phase interfaces can be estimated using the well-known correlation for gas flow over a flat plate fluid-solid system[15], where the average mass transfer coefficient *k* is calculated from the Sherwood number ($\overline{Sh}$):

$$k = \overline{Sh}\frac{D}{L} = 0.664Re^{0.5}Sc^{0.333}\frac{D}{L} \tag{2}$$

where *Re* and *Sc* are the Reynolds number and Schmidt number (both dimensionless quantities), respectively, *D* is the diffusivity of CO_2 in the gas phase, and *L* is the length of electrode in the direction of flow. The *Re* and *Sc* numbers for CO_2 convection at a three phase interface are described as:

$$Re = \frac{V_{\mathrm{f}}}{v} \tag{3}$$

$$Sc = \frac{v}{D} \tag{4}$$

where V_{f} is the effective CO_2 flow velocity and *v* is the momentum velocity which relates to the apparent kinematic viscosity of the gas phase.

Basic principle of HPTS as a CO_2 fluorescent probe. The development of HPTS-based CO_2 fluorescent probe occurred in three stages: 1) the discovery of HPTS as a fluorescence indicator for pH measurement by O. S. Wolfbeis *et al.*[16]; 2) the reversible detection of gaseous CO_2 concentration by A. Mills and Q. Chang[17] and 3) the incorporation of HPTS with GDE for in-situ CO_2 concentration detection developed in our study.

In brief, the detection principle relies on two basic concepts. Firstly, HPTS is a pH-sensitive fluorophore. The pH of the medium is measured by means of the fluorescence intensity of HPTS as a result of the following process:

$$\mathrm{DH} \overset{K_{\mathrm{a}}}{\rightleftharpoons} \mathrm{D^-} + \mathrm{H^+} \tag{5}$$

where DH and D^- are the protonated and deprotonated forms of HPTS, respectively, and K_a is the acid-dissociation constant. Secondly, CO_2 is able to induce a change in the pH of an aqueous medium via the formation of carbonic acid and its subsequent deprotonation reactions, as described by the following equations:

$$H_2CO_3 \rightleftharpoons H^+ + HCO_3{}^- \quad (6)$$

$$HCO_3{}^- \rightleftharpoons H^+ + CO_3{}^{2-} \quad (7)$$

To this end, CO_2 dissolution processes cause fluorescence intensity variations of HPTS-containing aqueous media (i.e. fluorescence intensity ↔ pH ↔ CO_2). The challenge for the current study was how to achieve CO_2 detection in gas phase, where no water (or non-aqueous non-polar medium) was available. We adopted the method developed by A. Mills and Q. Chang (Analyst 1993, 118, 839-843), using a phase transfer agent (tetraoctylammonium cation, Q^+) to form a hydrated anion pair with deprotonated HPTS ($Q^+D^-{\cdot}xH_2O$). In this way, HPTS could act as a gaseous CO_2 detector, because associated with the HPTS in the ion-pair form is the necessary H_2O for CO_2 to interact with. It should be noted that the HPTS-based sensor is separated from basic electrolyte environment, since it is coated on the reverse side of GDL and cover by a hydrophobic layer. Thus, the pH of the electrolyte does not affect the reliability of the sensor.

Approximation of interfacial CO_2 concentration. Since the HPTS-based CO_2 detector was anchored at the reverse side of the GDL, the detected region is about 100 μm (the thickness of GDL) away from the three phase interfaces. This means that the detected local CO_2 concentration ($[CO_2]_d$) may not be exactly identical to $[CO_2]_i$. Here we have assumed that $[CO_2]_d \approx [CO_2]_i$. The rationality of this treatment can be explained from Fick's first law:

$$J = -D\frac{\partial C}{\partial X} \quad (8)$$

Where J is diffusion flux, D is the diffusion coefficient of CO_2 in gas phase (~0.1 $cm^2\ s^{-1}$), $\frac{\partial C}{\partial X}$ is the CO_2 concentration gradient between detected region and three phase interface. Since both the gas phase and liquid phase of the cell were sealed without external flow before FES measurements, J equals to the mass transfer flux N, thus can be estimated from the CO partial current density as discussed in the manuscript. In this way, the difference between $[CO_2]_d$ and $[CO_2]_i$ is calculated to be ~0.05 mM even at a CO partial current density of 100 mA cm^{-2}, which is negligible compared

to the substantially reduced interfacial CO_2 concentration (>1 mM) at this current density.

Calculation of CO_2 solubility in 1 M $KHCO_3$. Assuming CO_2 behaves as an ideal gas, the initial CO_2 concentration in a 10% CO_2/90% Ar gas mixture is about 4.1 mM. The CO_2 solubility in pure water $[CO_2]_{aq,0}$ can be found using Henry's Law:

$$[\mathrm{CO_2}]_{\mathrm{aq,0}} = K_0[\mathrm{CO_2}]_{\mathrm{g}} \tag{9}$$

Where $[CO_2]_g$ is the equilibrium CO_2 concentration in the gas phase. The Henry coefficient K_0 for CO_2 can be found as a function of temperature[18]:

$$\ln K_0 = 93.4517\left(\frac{100}{T}\right) - 60.2409 + 23.3585\, ln\left(\frac{T}{100}\right) \tag{10}$$

At room temperature (298 K), the CO_2 solubility in 10% CO_2/90% Ar saturated pure water is thus calculated to be 3.4 mM. In a 1 M $KHCO_3$ electrolyte, the CO_2 solubility will be lower than that in pure water due to the salting out effect as described by the Sechenov Equation[19]:

$$\log(\frac{[\mathrm{CO_2}]_{\mathrm{aq,0}}}{[\mathrm{CO_2}]_{\mathrm{aq}}}) = K_{\mathrm{S}}C_{\mathrm{S}} \tag{11}$$

Where K_S is the Sechenov constant and C_s is the molar concentration of the electrolyte solution. K_S is a function of the specific ions in the electrolyte (K^+ and HCO_3^-) as well as the dissolved gas, and can be calculated using Supplementary Equations 12 and 13 described by Weisenberger et al.

$$K_{\mathrm{S}} = \sum(h_{\mathrm{ion}} + h_{\mathrm{G}}) \tag{12}$$

$$h_{\mathrm{G}} = h_{\mathrm{G,0}} + h_{\mathrm{T}}(T - 298.15) \tag{13}$$

The specific values used in these equations to calculate K_S are tabulated in Supplementary Table 3. The CO_2 solubility in a 10% CO_2/90% Ar saturated 1 M $KHCO_3$ solution was calculated to be 2.3 mM. In order to achieve the same initial CO_2 concentration in the TPC and DPC systems for in-situ interfacial CO_2 concentration measurements, a 17.8% CO_2/82.2% Ar saturated 1 M $KHCO_3$ medium was used in the DPC system (thus achieving a CO_2 solubility of 4.1 mM).

Supplementary References

[1] Verma, S. et al. Insights into the low overpotential electroreduction of CO_2 to CO on a supported gold catalyst in an alkaline flow electrolyzer. *ACS Energy Lett.* **3**, 193-198 (2017).

[2] Liu, M. *et al.* Enhanced electrocatalytic CO_2 reduction via field-induced reagent concentration. *Nature* **537**, 382-386 (2016).

[3] Zhu, W. et al. Active and selective conversion of CO_2 to CO on ultrathin Au nanowires. *J. Am. Chem. Soc.* **136**, 16132-16135 (2014).

[4] Chen, Y., Li, C.W., Kanan, M.W. Aqueous CO_2 reduction at very low overpotential on oxide-derived Au nanoparticles. *J. Am. Chem. Soc.* **134**, 19969-19972 (2012).

[5] Hall, A.S., Yoon, Y., Wuttig, A., Surendranath, Y. Mesostructure-induced selectivity in CO_2 reduction catalysis. *J. Am. Chem. Soc.* **137**, 14834-14837 (2015).

[6] Lee, H.-E. et al. Concave rhombic dodecahedral Au nanocatalyst with multiple high-index facets for CO_2 reduction. *ACS Nano* **9**, 8384-8393 (2015).

[7] Li, J. et al. Efficient electrocatalytic CO_2 reduction on a three-phase interface. *Nat. Catal.* **1**, 592-600 (2018).

[8] Cao, Z. et al. A molecular surface functionalization approach to tuning nanoparticle electrocatalysts for carbon dioxide reduction. *J. Am. Chem. Soc.* **138**, 8120-8125 (2016).

[9] Rogers, C. et al. Synergistic enhancement of electrocatalytic CO_2 reduction with gold nanoparticles embedded in functional graphene nanoribbon composite electrodes. *J. Am. Chem. Soc.* **139**, 4052-4061 (2017).

[10] Sun, K. et al. Ultrahigh mass activity for carbon dioxide reduction enabled by gold-iron core-shell nanoparticles. *J. Am. Chem. Soc.* **139**, 15608-15611 (2017).

[11] Kauffman, D.R., Alfonso, D., Matranga, C., Qian, H., Jin, R. Experimental and computational investigation of Au_{25} clusters and CO_2: a unique interaction and enhanced electrocatalytic activity. *J. Am. Chem. Soc.* **134**, 10237-10243 (2012).

[12] Todoroki, N. et al. Surface atomic arrangement dependence of electrochemical CO_2 Reduction on gold: Online electrochemical mass spectrometric study on low-index Au(hkl) surfaces. *ACS Catal.* **9**, 1383-1388 (2019).

[13] Jhong, H.M. et al. Gold nanoparticles on polymer-wrapped carbon nanotubes: An efficient and

selective catalyst for the electroreduction of CO_2. *ChemPhysChem* **18**, 3274-3279 (2017).

[14] Zhao, S. et al. Influence of atomic-level morphology on catalysis: The case of sphere and rod-like gold nanoclusters for CO_2 electroreduction. *ACS Catal.* **8**, 4996-5001 (2018).

[15] Burdyny, T. et al. Nanomorphology-enhanced gas-evolution intensifies CO_2 reduction electrochemistry. *ACS Sustain. Chem. Eng.* **5**, 4031-4040 (2017).

[16] Wolfbeis, O.S., Fürlinger, E., Kroneis, H., Marsoner, H. Fluorimetric analysis. *Anal. Chem.* **314**, 119-124 (1983).

[17] Mills, A., Chang, Q. Fluorescence plastic thin-film sensor for carbon dioxide. *Analyst* **118**, 839-843 (1993).

[18] Carroll, J.J., Slupsky, J.D., Mather, A.E. The solubility of carbon dioxide in water at low pressure. *J. Phys. Chem. Ref. Data* **20**, 1201-1209 (1991).

[19] Gupta, N., Gattrell, M., MacDougall, B. Calculation for the cathode surface concentrations in the electrochemical reduction of CO_2 in $KHCO_3$ solutions. *J. Appl. Electrochem.* **36**, 161-172 (2005).
