# Supplementary figures and images for: Efficient wettability-controlled electroreduction of CO2 to CO at Au/C interfaces
Source: Nat Commun. 2020 Jun 15;11:3028. doi: 10.1038/s41467-020-16847-9 (PMC7295780; doi:10.1038/s41467-020-16847-9)

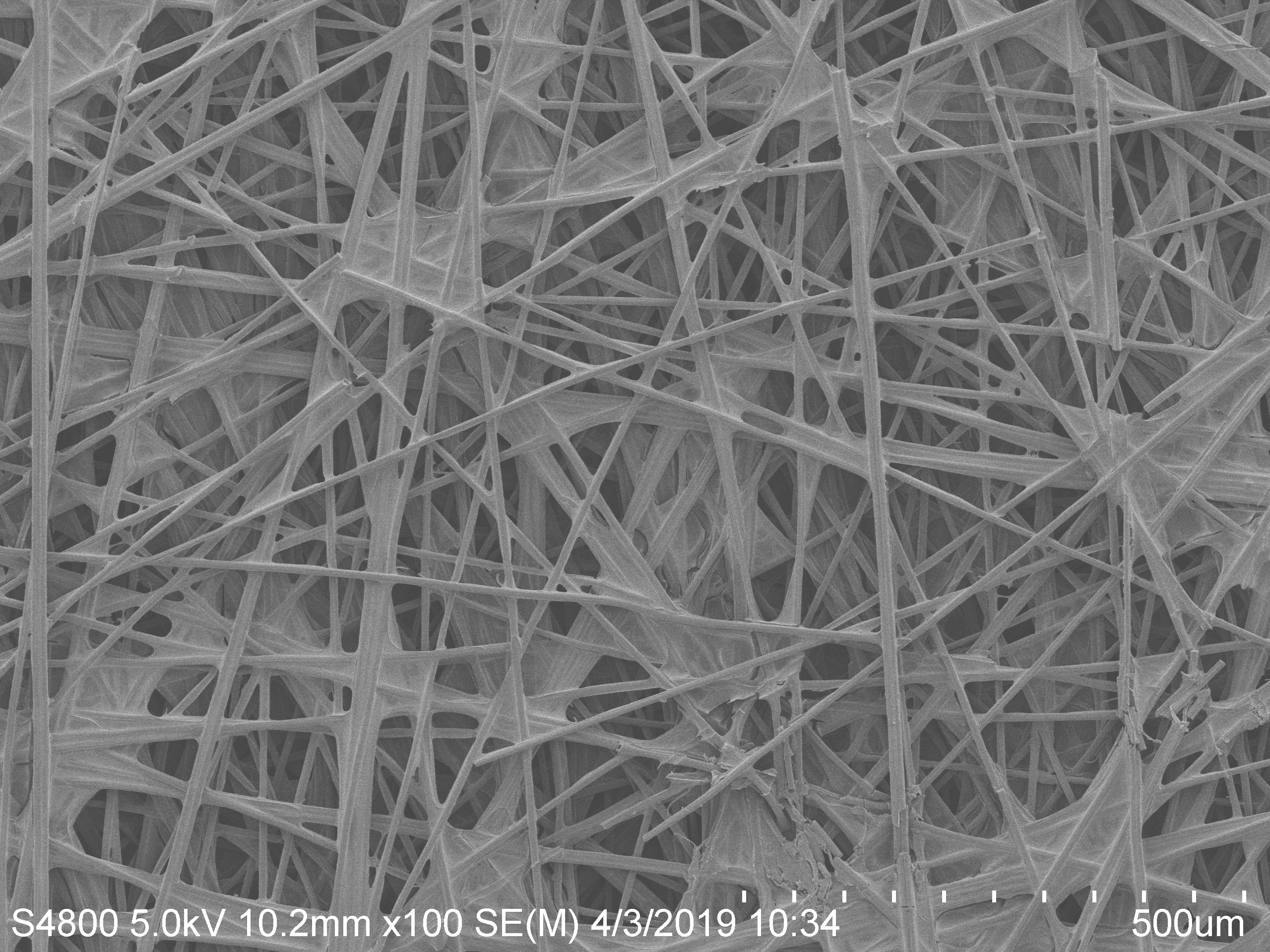

Supplement: Supplementary file 2 — Source Data [file 41467_2020_16847_MOESM2_ESM.zip › Source Data/Figure 1/Figure 1a.tif]

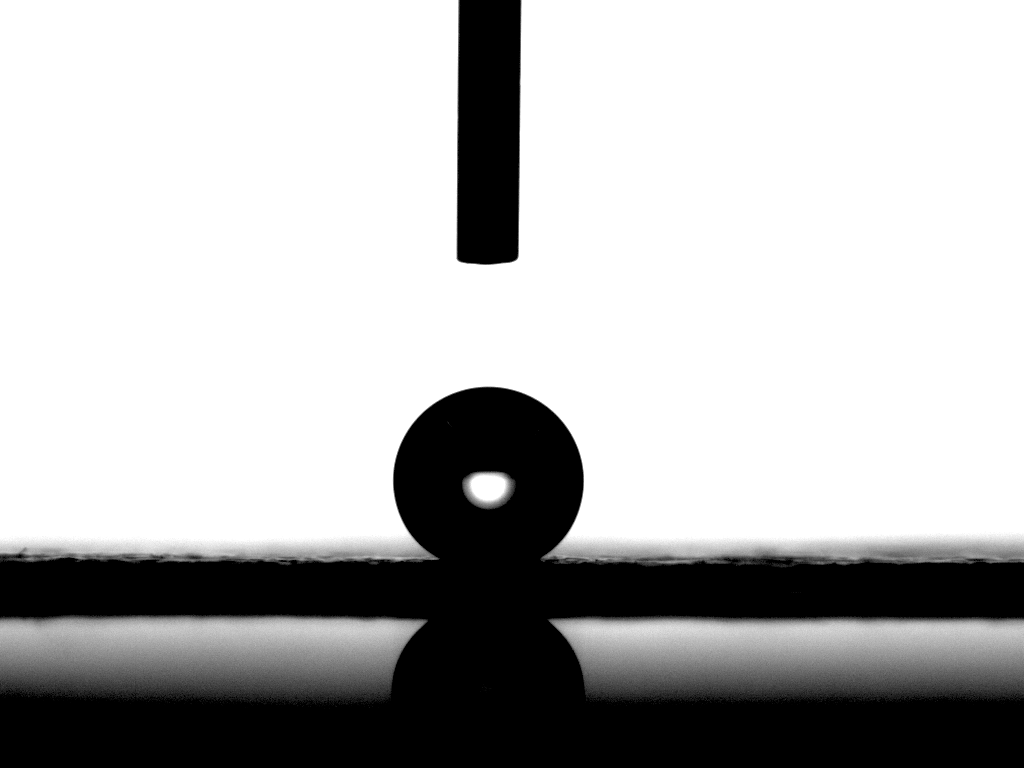

Supplement: Supplementary file 2 — Source Data [file 41467_2020_16847_MOESM2_ESM.zip › Source Data/Figure 1/Figure 1a_insert.bmp]

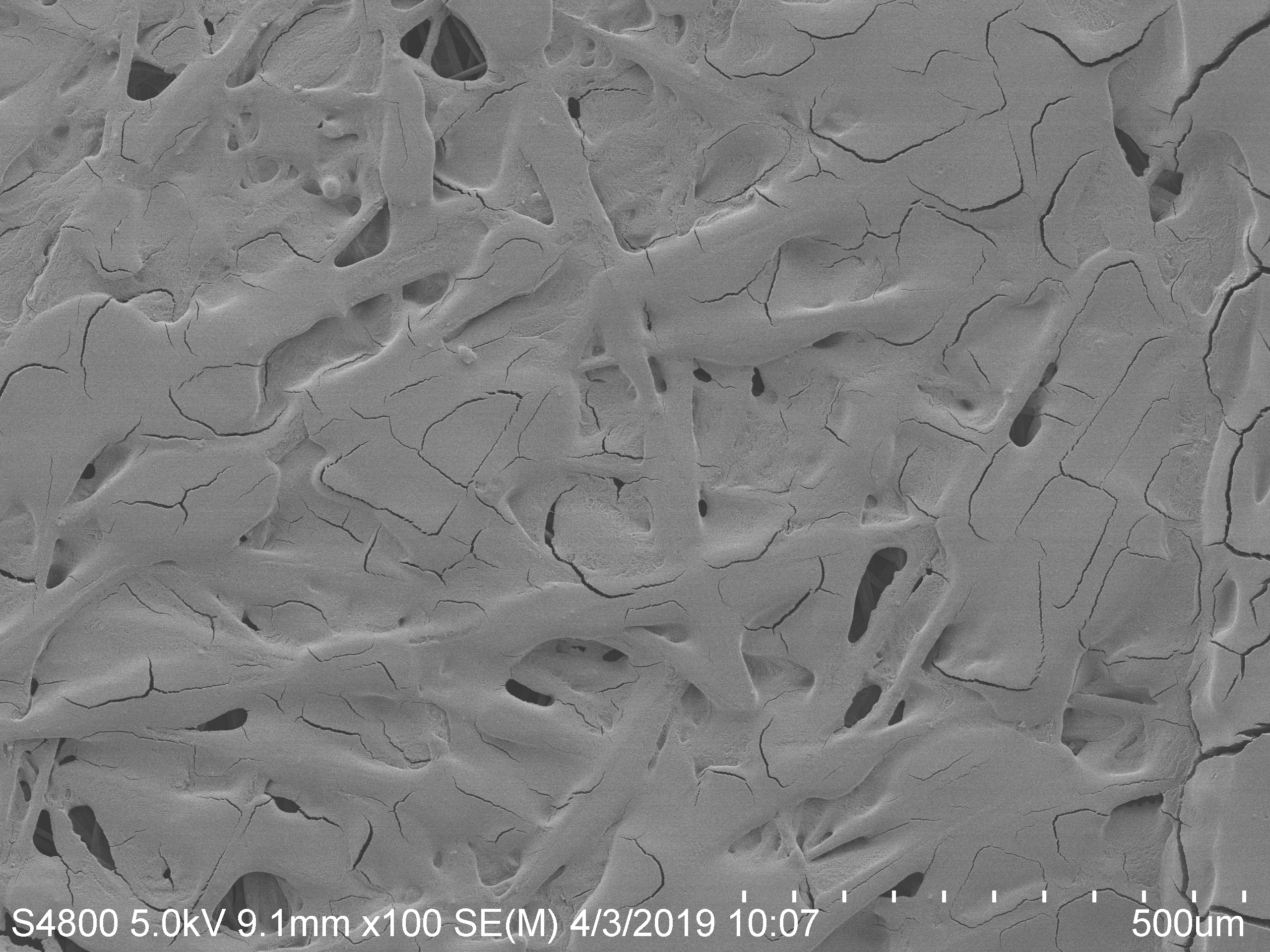

Supplement: Supplementary file 2 — Source Data [file 41467_2020_16847_MOESM2_ESM.zip › Source Data/Figure 1/Figure 1b.tif]

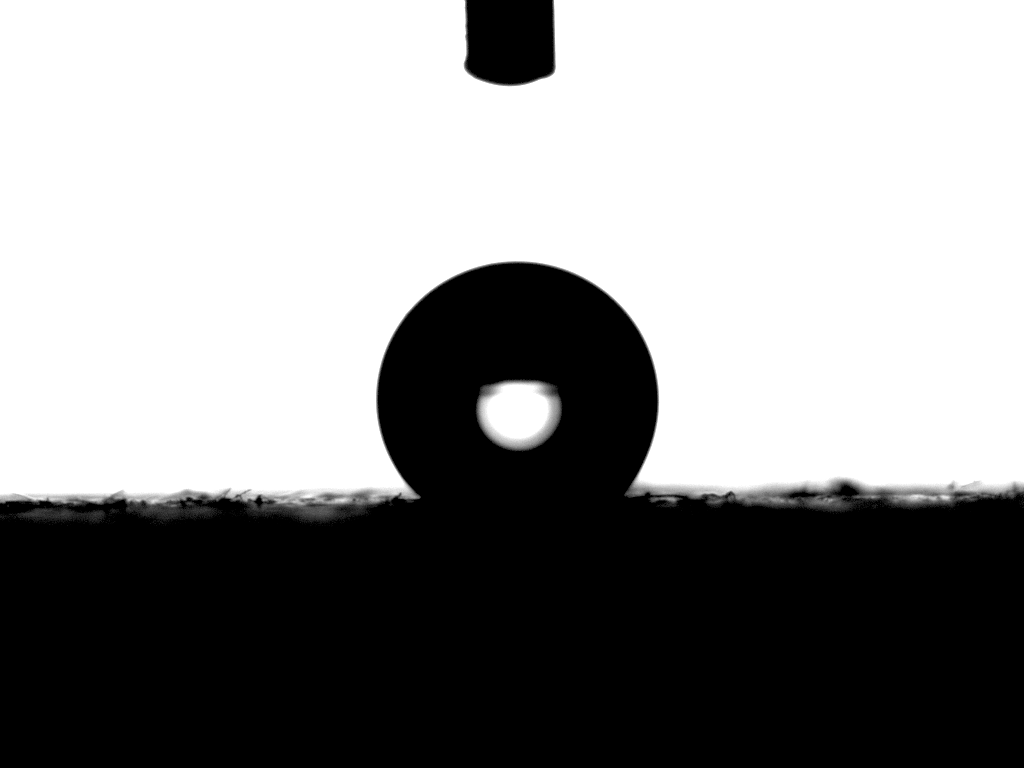

Supplement: Supplementary file 2 — Source Data [file 41467_2020_16847_MOESM2_ESM.zip › Source Data/Figure 1/Figure 1b_insert.bmp]

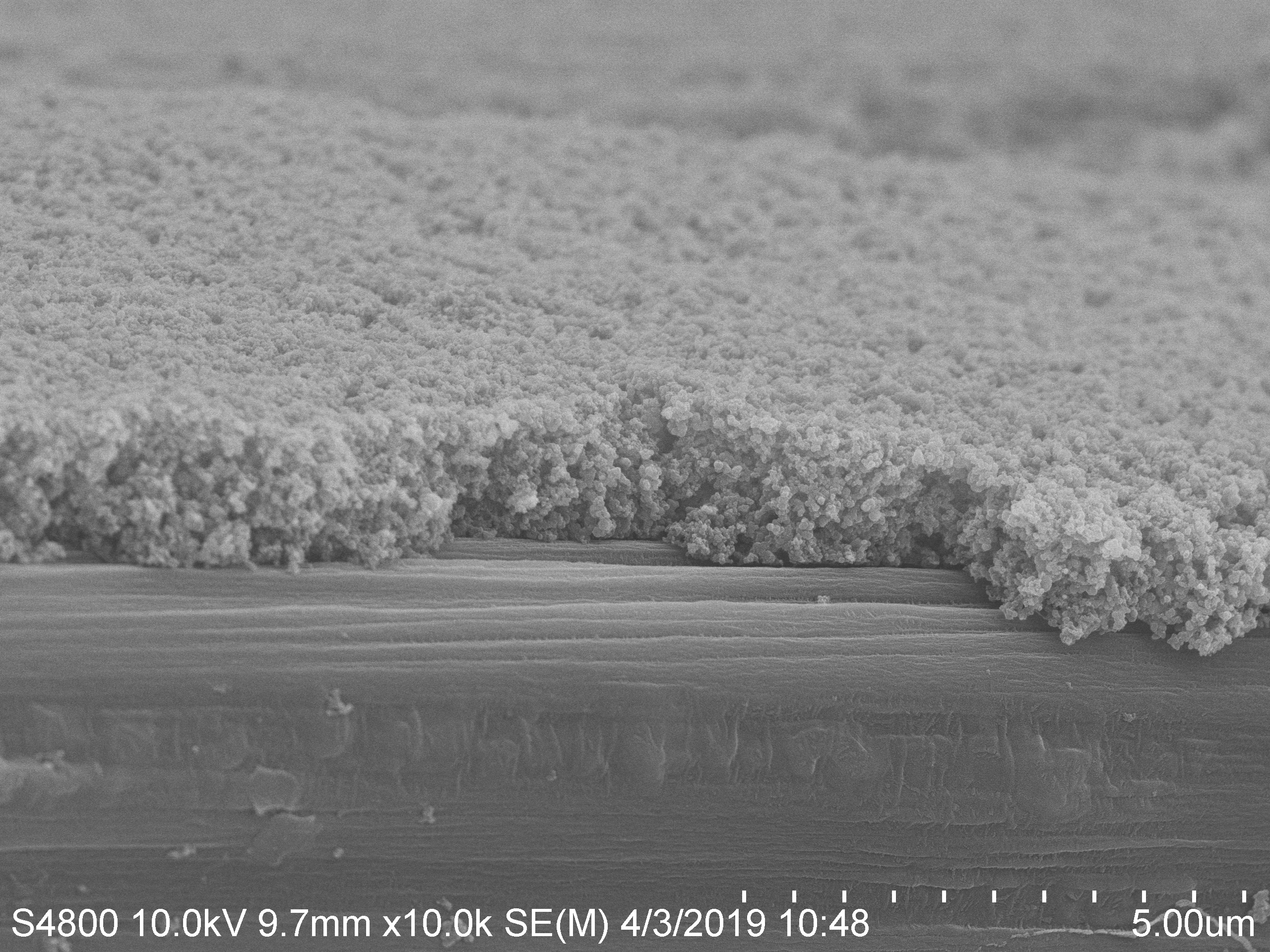

Supplement: Supplementary file 2 — Source Data [file 41467_2020_16847_MOESM2_ESM.zip › Source Data/Figure 1/Figure 1c.tif]

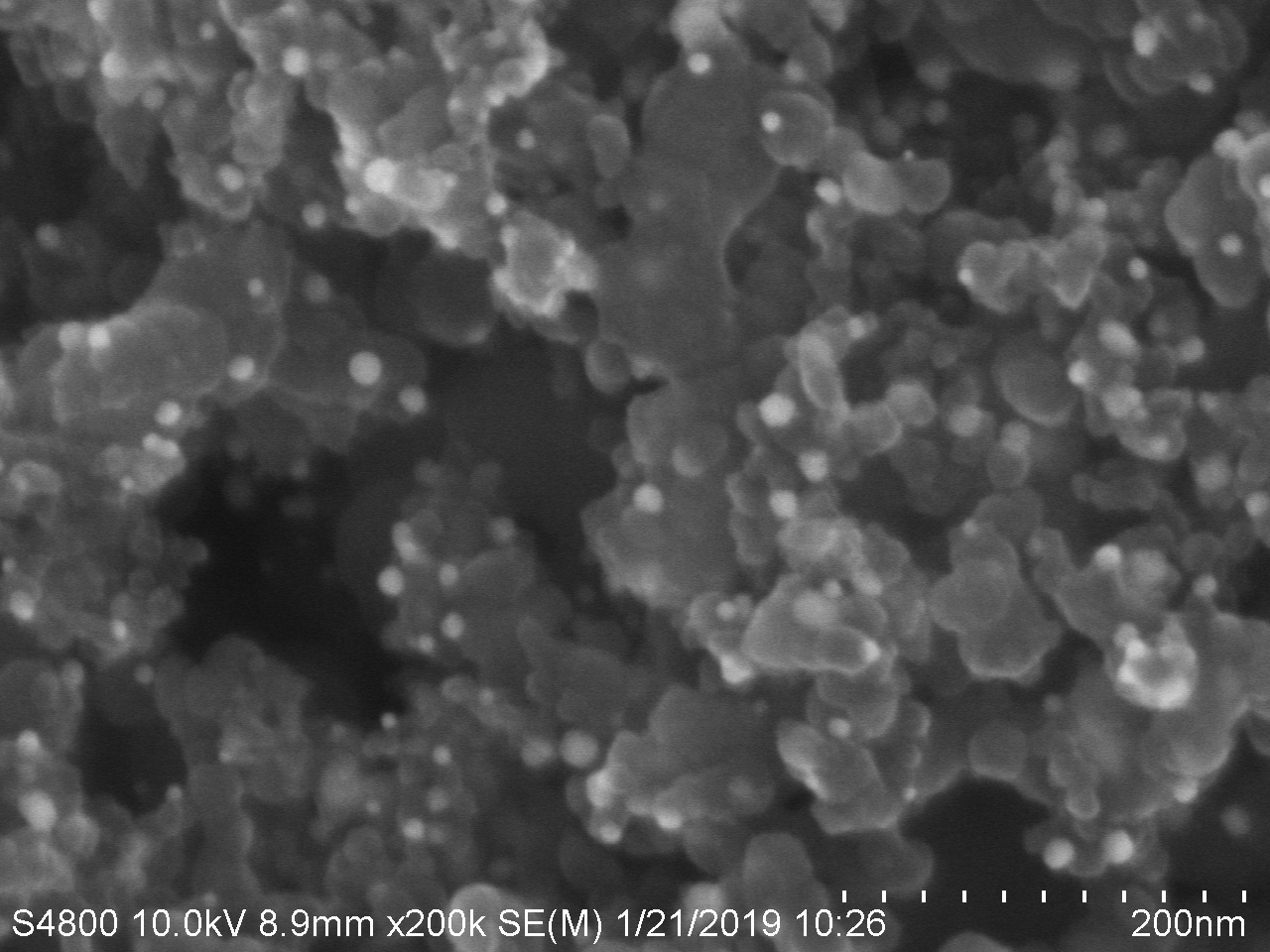

Supplement: Supplementary file 2 — Source Data [file 41467_2020_16847_MOESM2_ESM.zip › Source Data/Figure 1/Figure 1e.tif]

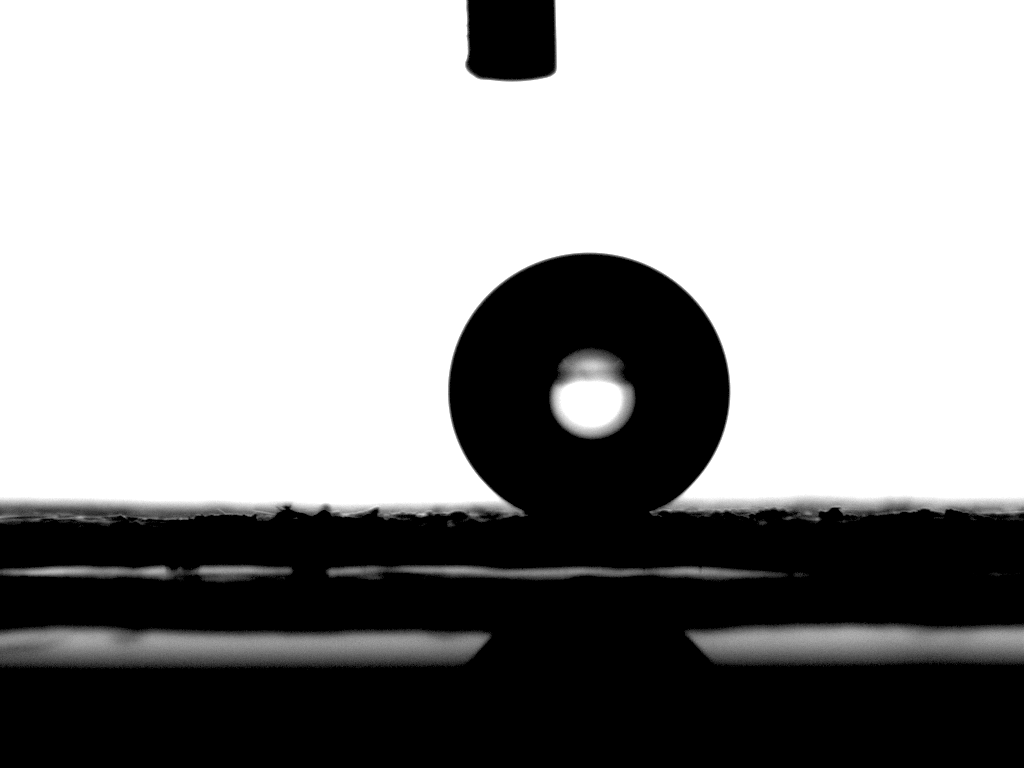

Supplement: Supplementary file 2 — Source Data [file 41467_2020_16847_MOESM2_ESM.zip › Source Data/Figure 2/Figure 1a_insert_1.bmp]

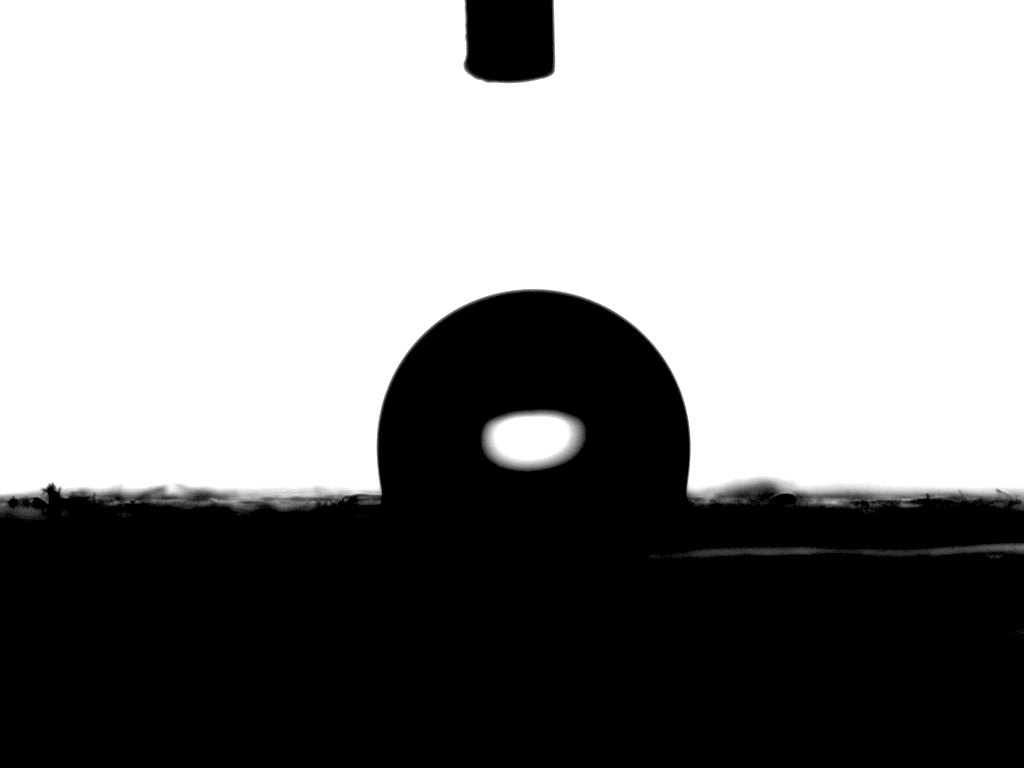

Supplement: Supplementary file 2 — Source Data [file 41467_2020_16847_MOESM2_ESM.zip › Source Data/Figure 2/Figure 1a_insert_3.bmp]

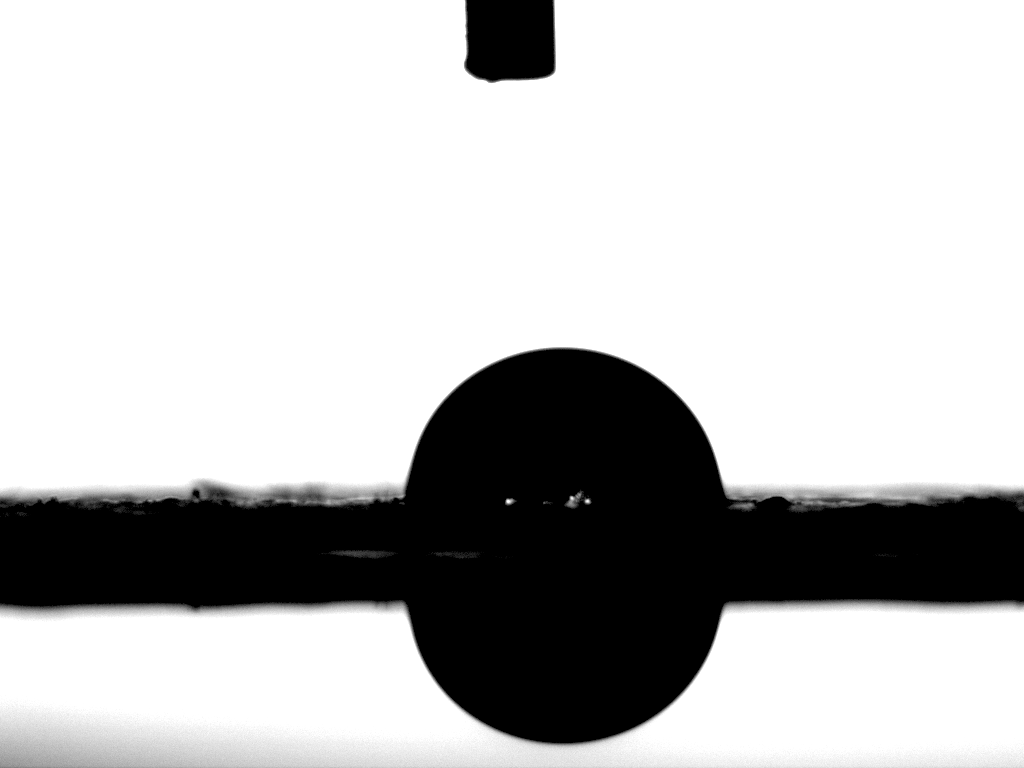

Supplement: Supplementary file 2 — Source Data [file 41467_2020_16847_MOESM2_ESM.zip › Source Data/Figure 2/Figure 1a_insert_4.bmp]

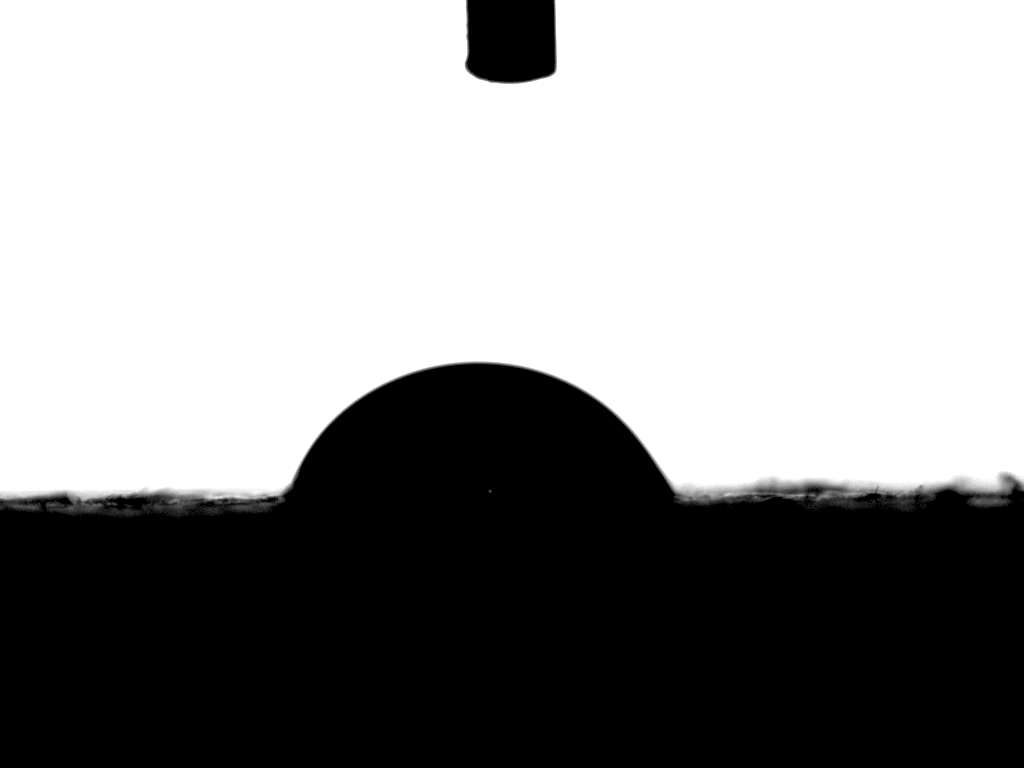

Supplement: Supplementary file 2 — Source Data [file 41467_2020_16847_MOESM2_ESM.zip › Source Data/Figure 2/Figure 1a_insert_5.bmp]

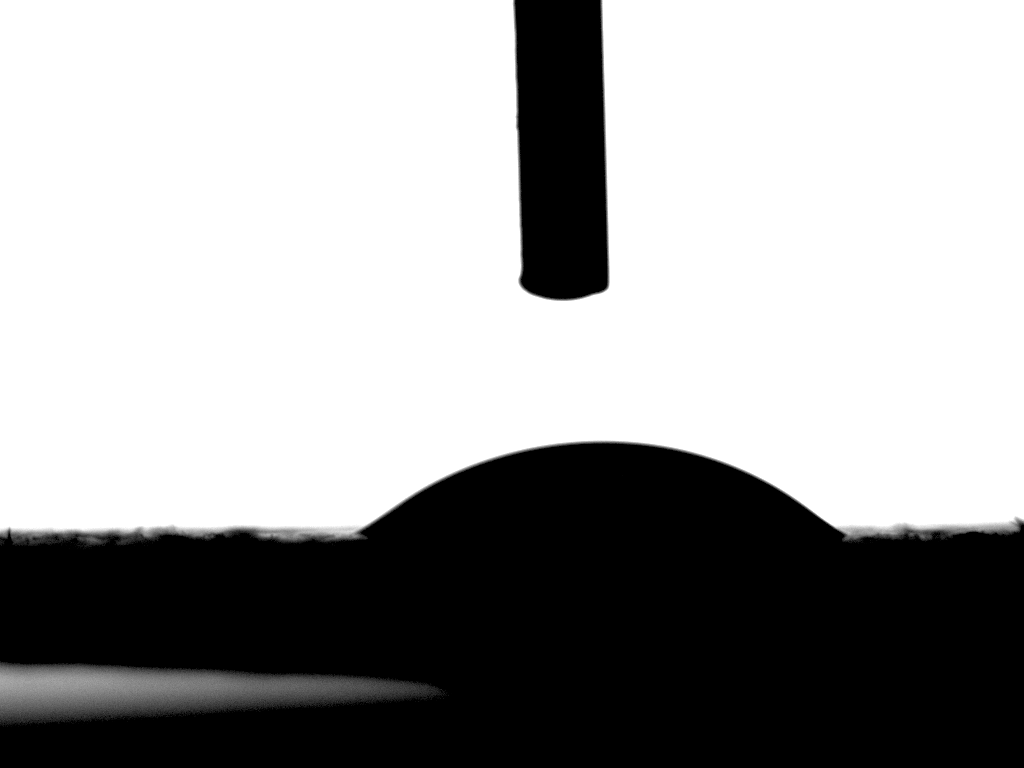

Supplement: Supplementary file 2 — Source Data [file 41467_2020_16847_MOESM2_ESM.zip › Source Data/Figure 2/Figure 1a_insert_6.bmp]

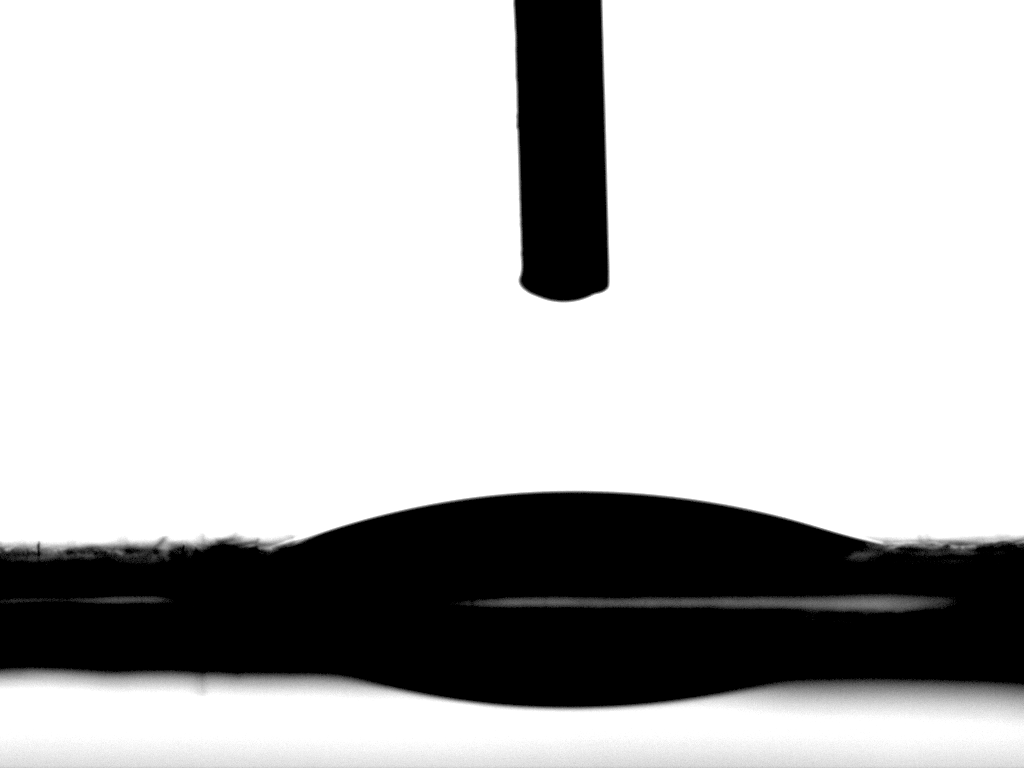

Supplement: Supplementary file 2 — Source Data [file 41467_2020_16847_MOESM2_ESM.zip › Source Data/Figure 2/Figure 1a_insert_7.bmp]

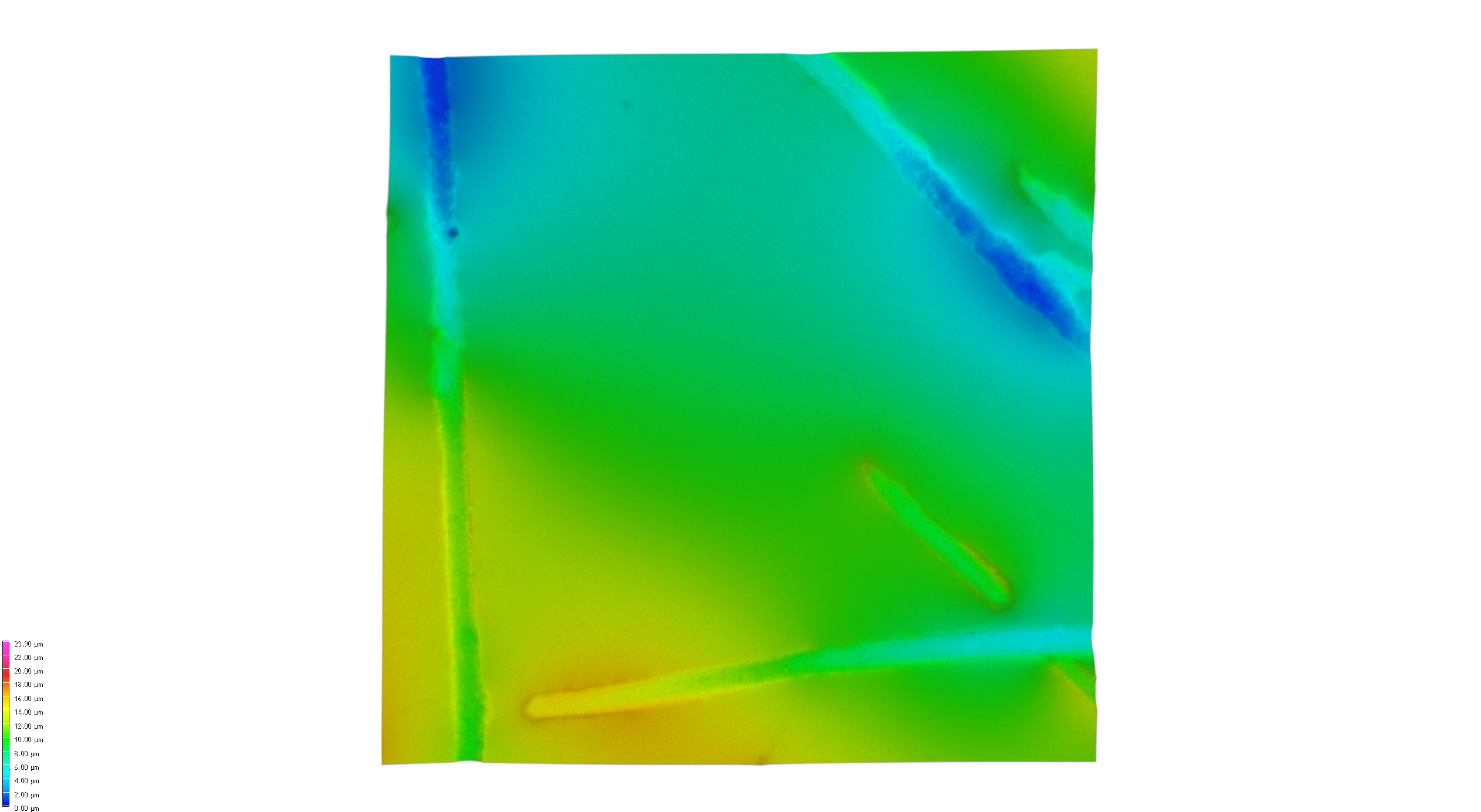

Supplement: Supplementary file 2 — Source Data [file 41467_2020_16847_MOESM2_ESM.zip › Source Data/Figure 3/Figure 3a.jpg]

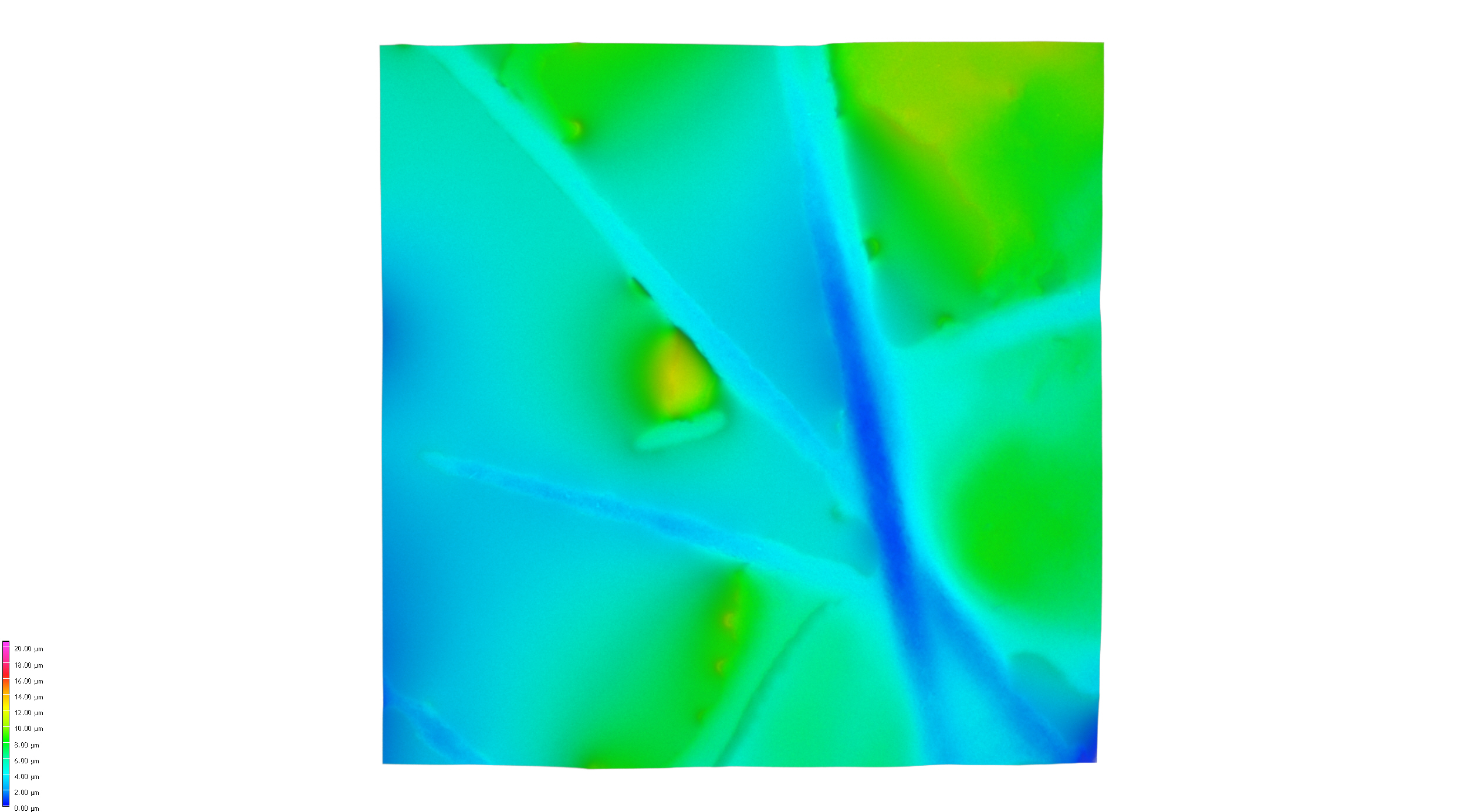

Supplement: Supplementary file 2 — Source Data [file 41467_2020_16847_MOESM2_ESM.zip › Source Data/Figure 3/Figure 3b.jpg]

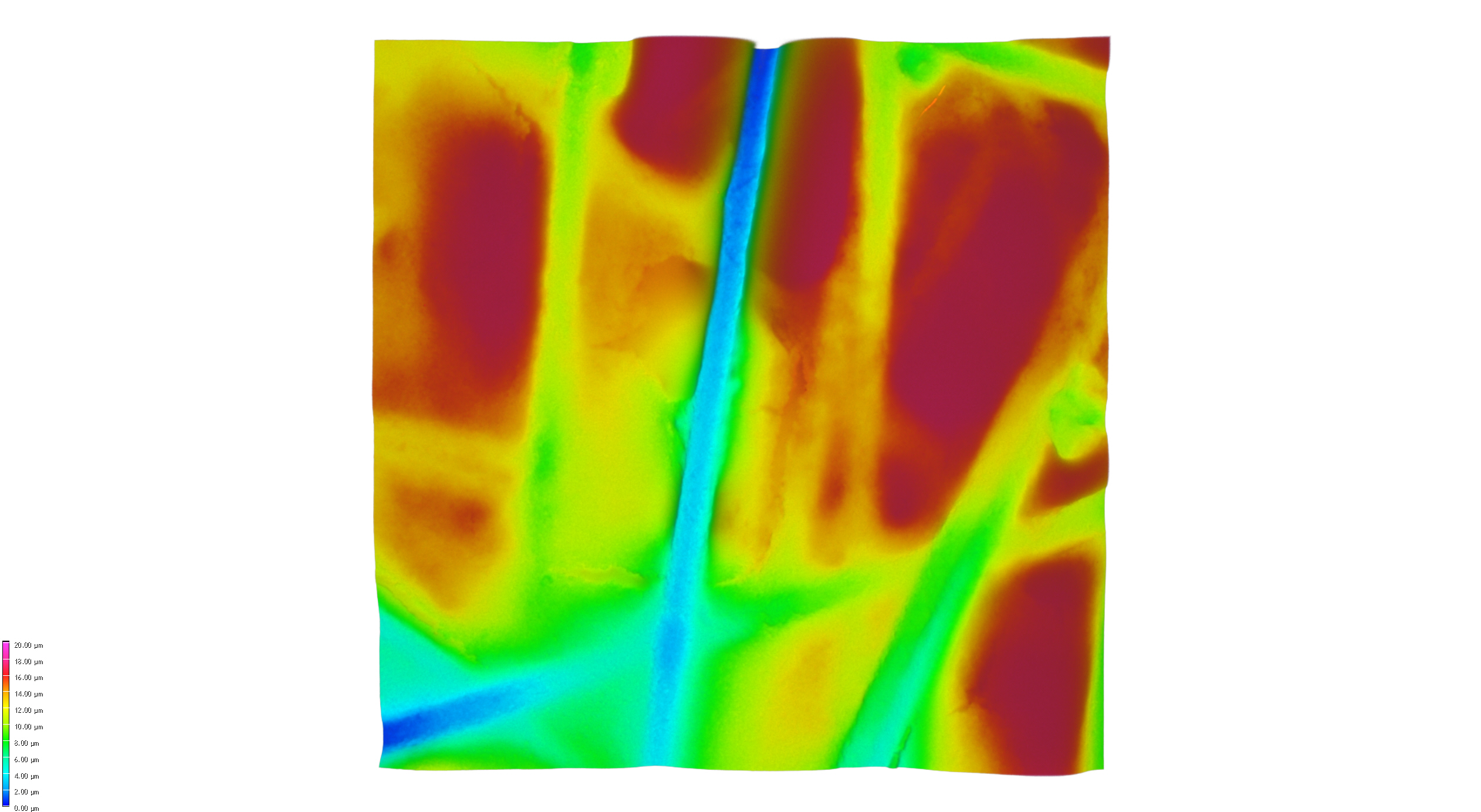

Supplement: Supplementary file 2 — Source Data [file 41467_2020_16847_MOESM2_ESM.zip › Source Data/Figure 3/Figure 3c.jpg]

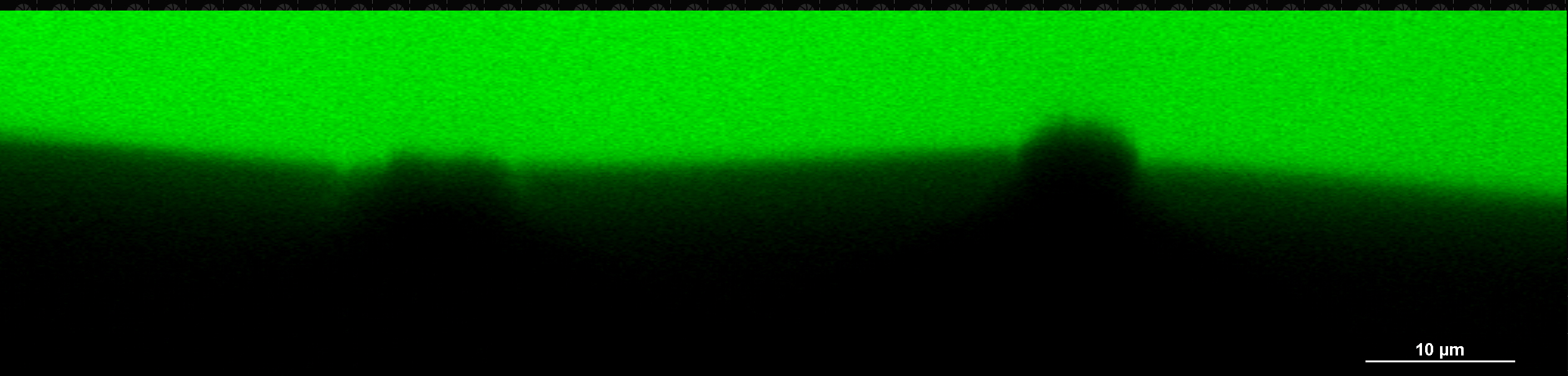

Supplement: Supplementary file 2 — Source Data [file 41467_2020_16847_MOESM2_ESM.zip › Source Data/Figure 3/Figure 3d_1_1.jpg]

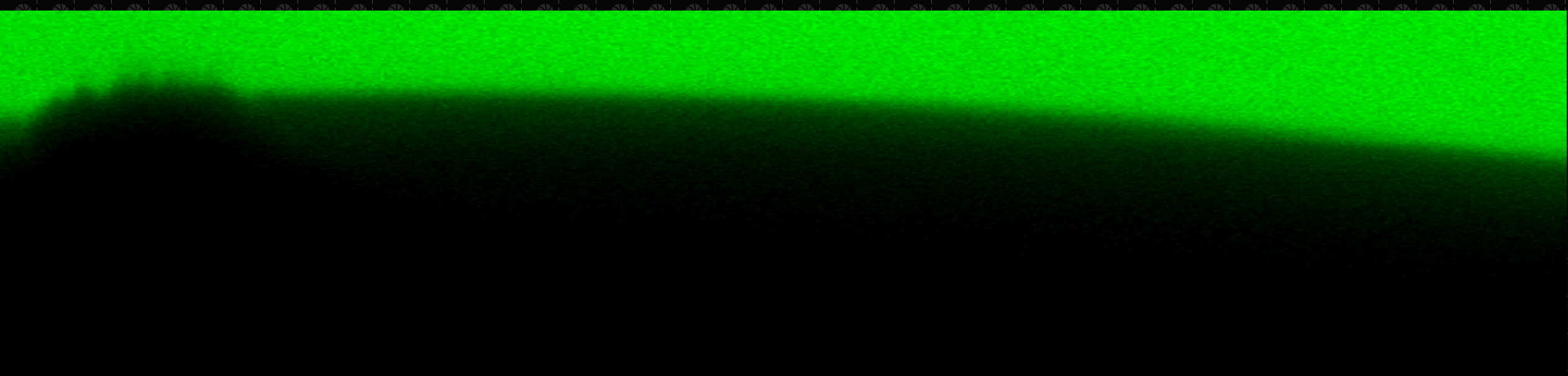

Supplement: Supplementary file 2 — Source Data [file 41467_2020_16847_MOESM2_ESM.zip › Source Data/Figure 3/Figure 3d_1_2.jpg]

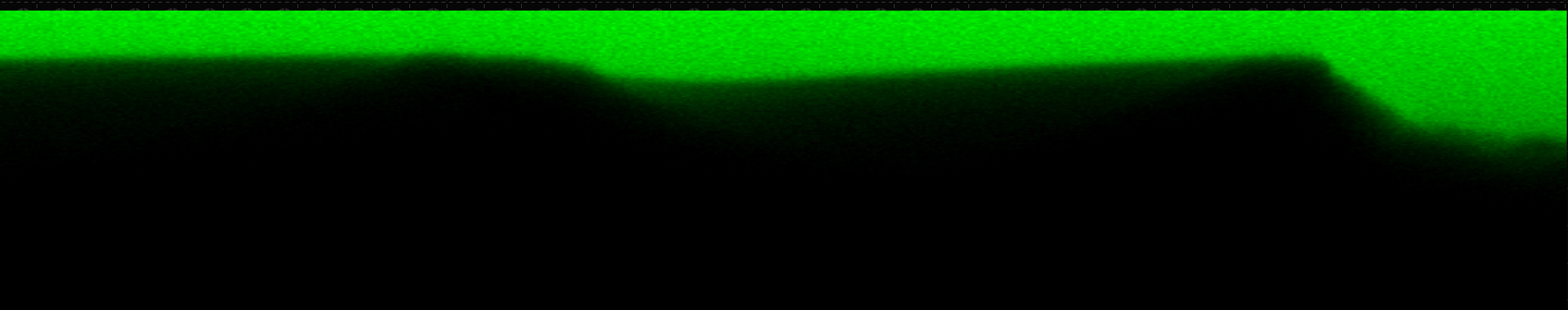

Supplement: Supplementary file 2 — Source Data [file 41467_2020_16847_MOESM2_ESM.zip › Source Data/Figure 3/Figure 3d_2_1.jpg]

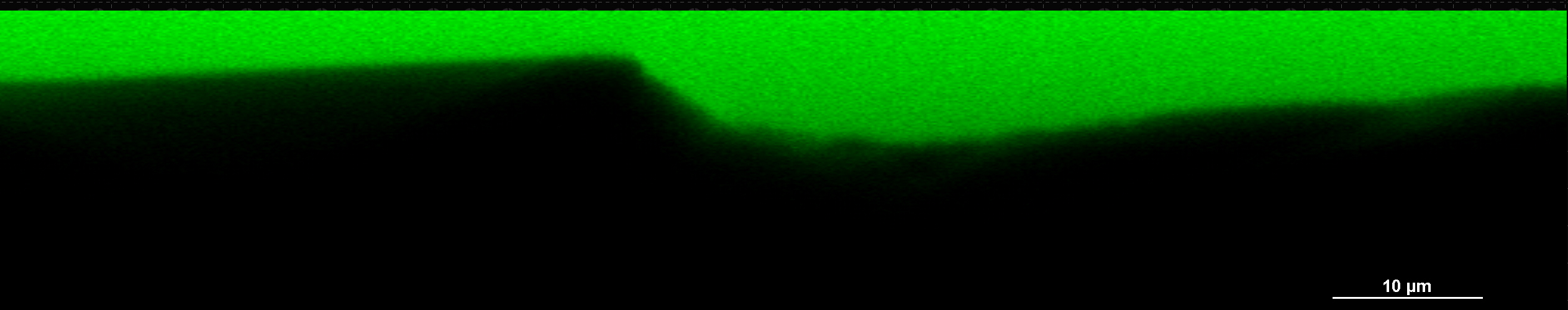

Supplement: Supplementary file 2 — Source Data [file 41467_2020_16847_MOESM2_ESM.zip › Source Data/Figure 3/Figure 3d_2_2.jpg]

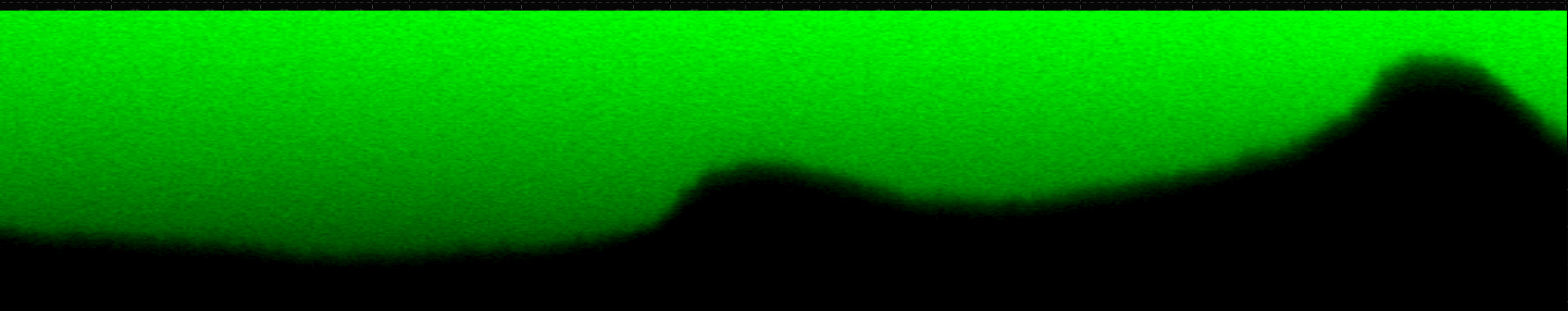

Supplement: Supplementary file 2 — Source Data [file 41467_2020_16847_MOESM2_ESM.zip › Source Data/Figure 3/Figure 3d_3_1.jpg]

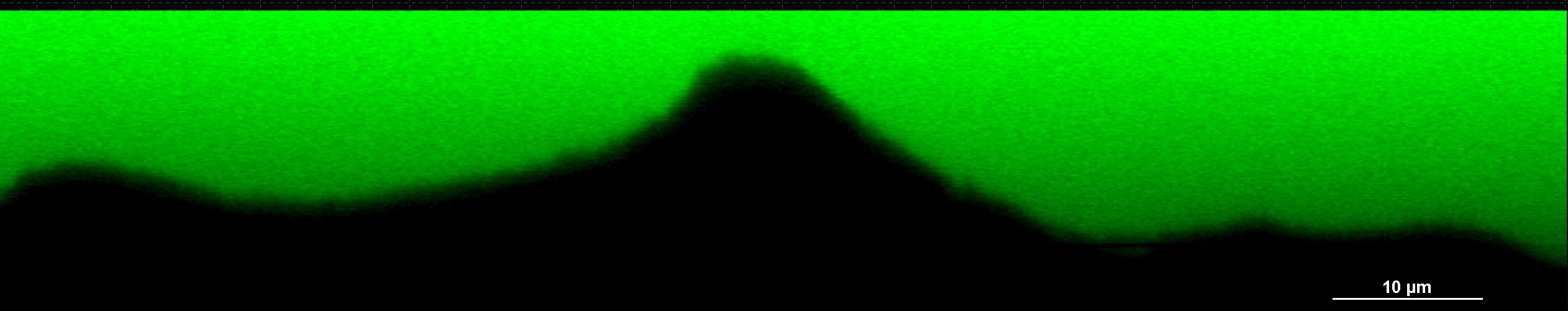

Supplement: Supplementary file 2 — Source Data [file 41467_2020_16847_MOESM2_ESM.zip › Source Data/Figure 3/Figure 3d_3_2.jpg]

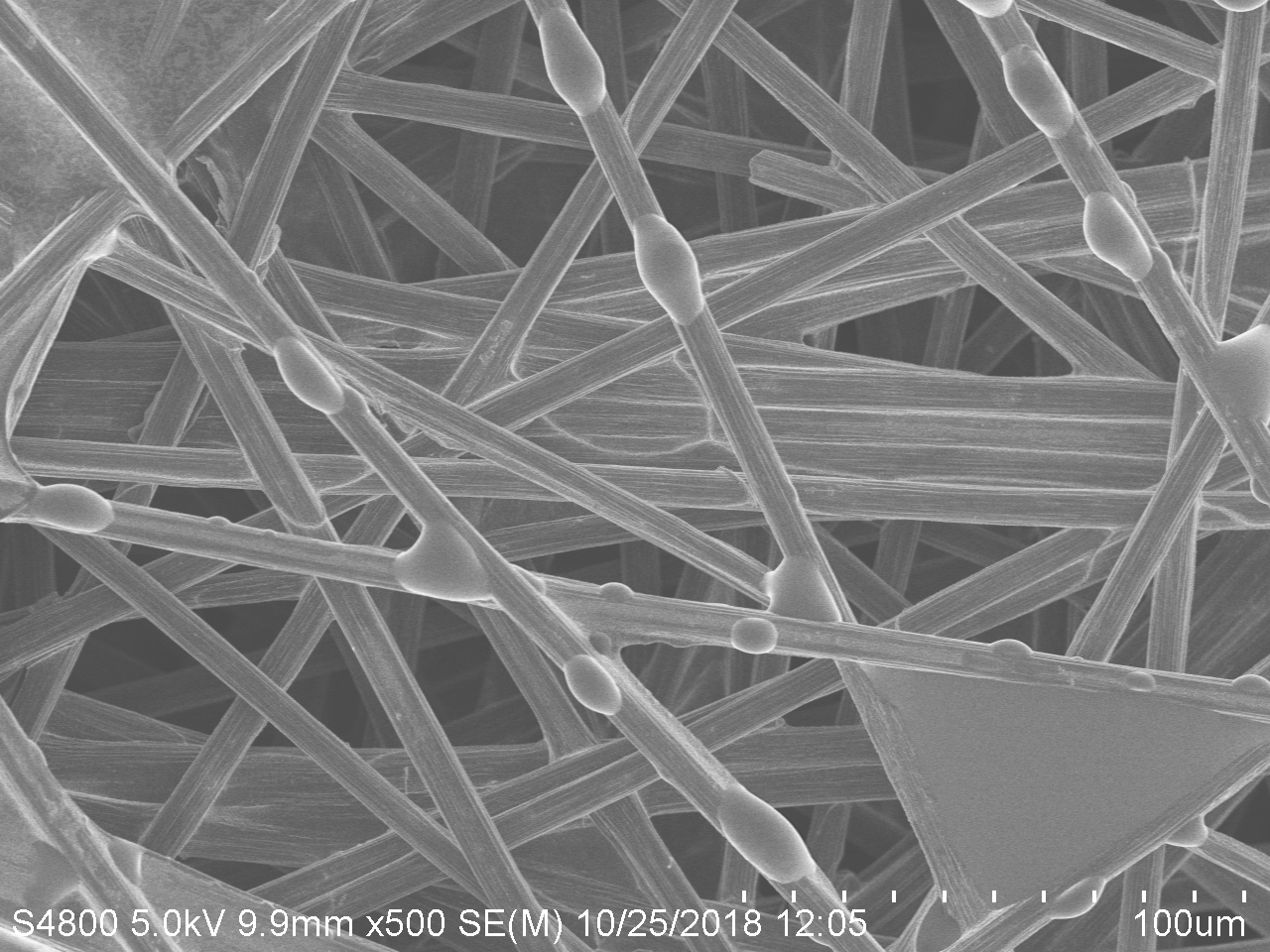

Supplement: Supplementary file 2 — Source Data [file 41467_2020_16847_MOESM2_ESM.zip › Source Data/Figure 4/Figure 4e.tif]

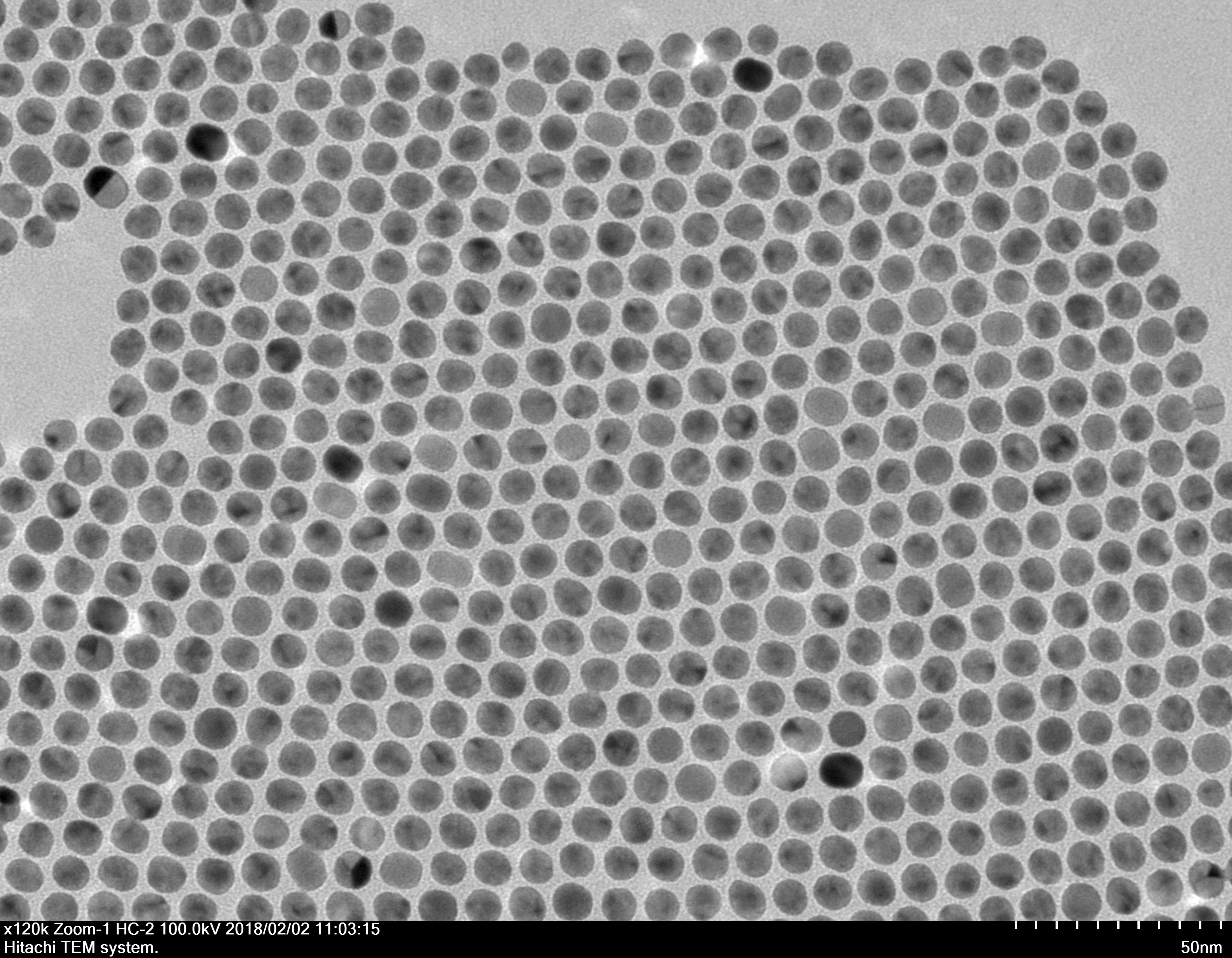

Supplement: Supplementary file 2 — Source Data [file 41467_2020_16847_MOESM2_ESM.zip › Source Data/SI/S1/S1a.tif]

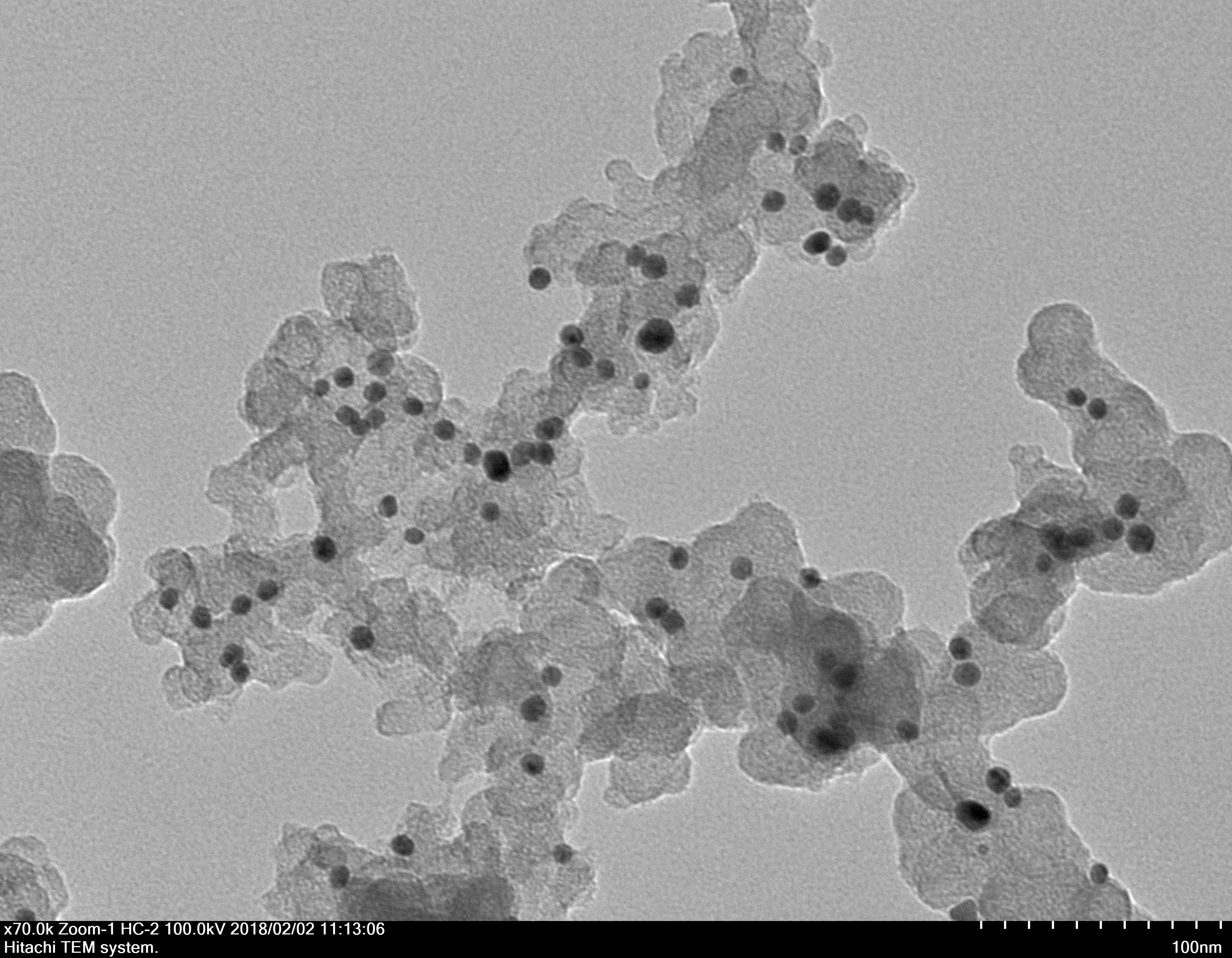

Supplement: Supplementary file 2 — Source Data [file 41467_2020_16847_MOESM2_ESM.zip › Source Data/SI/S1/S1b.tif]

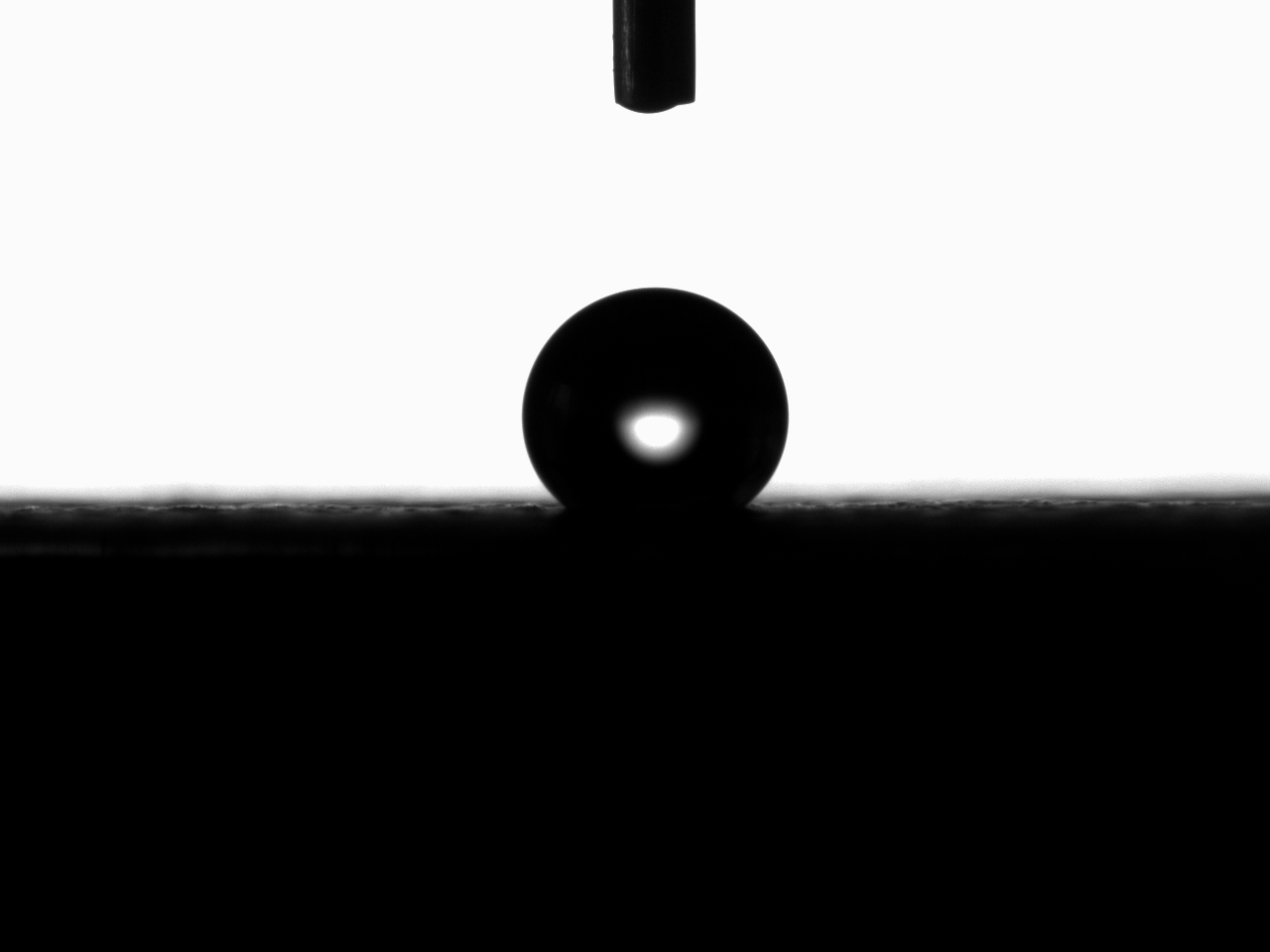

Supplement: Supplementary file 2 — Source Data [file 41467_2020_16847_MOESM2_ESM.zip › Source Data/SI/S11/S11a.bmp]

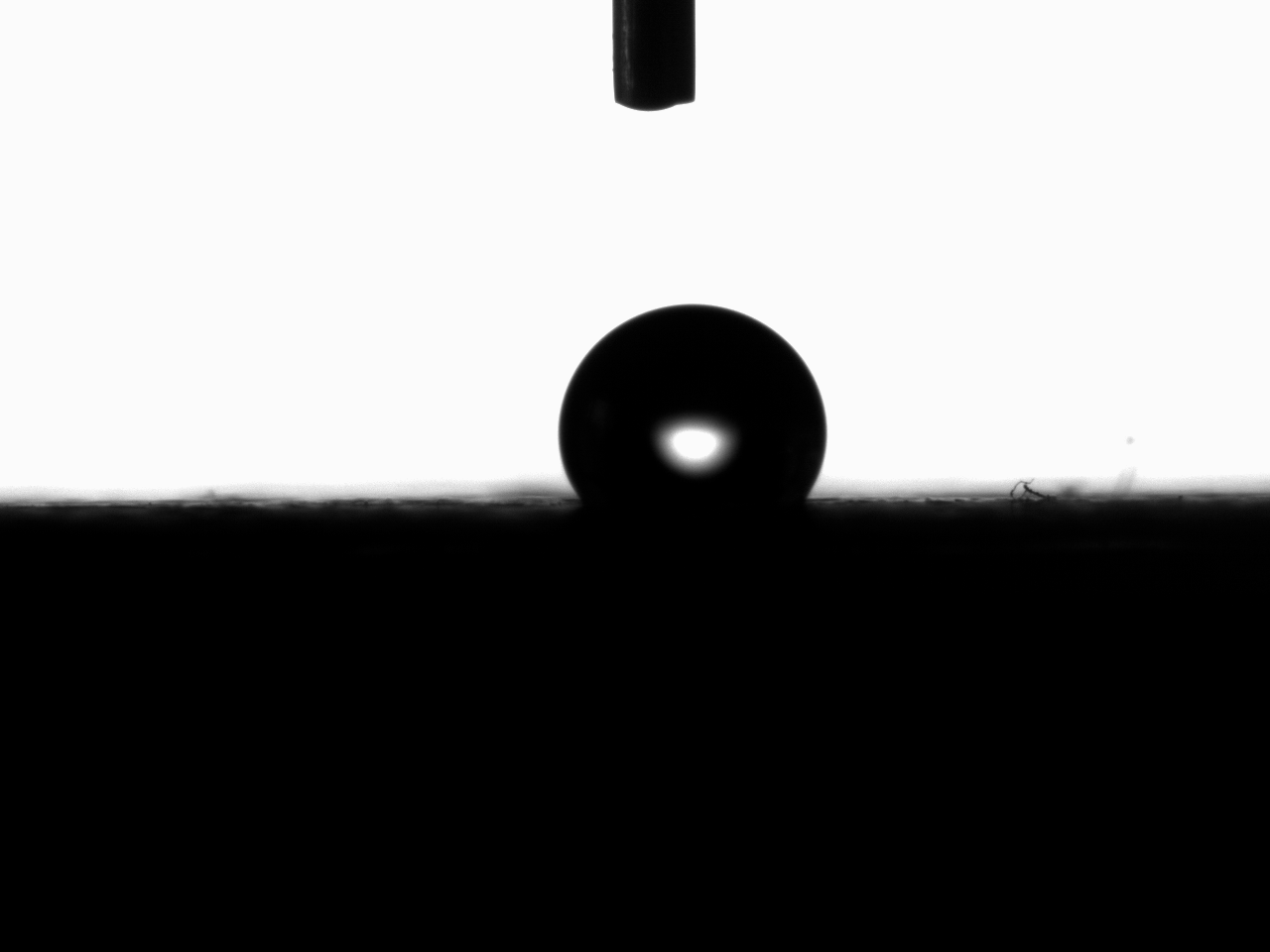

Supplement: Supplementary file 2 — Source Data [file 41467_2020_16847_MOESM2_ESM.zip › Source Data/SI/S11/S11b.bmp]

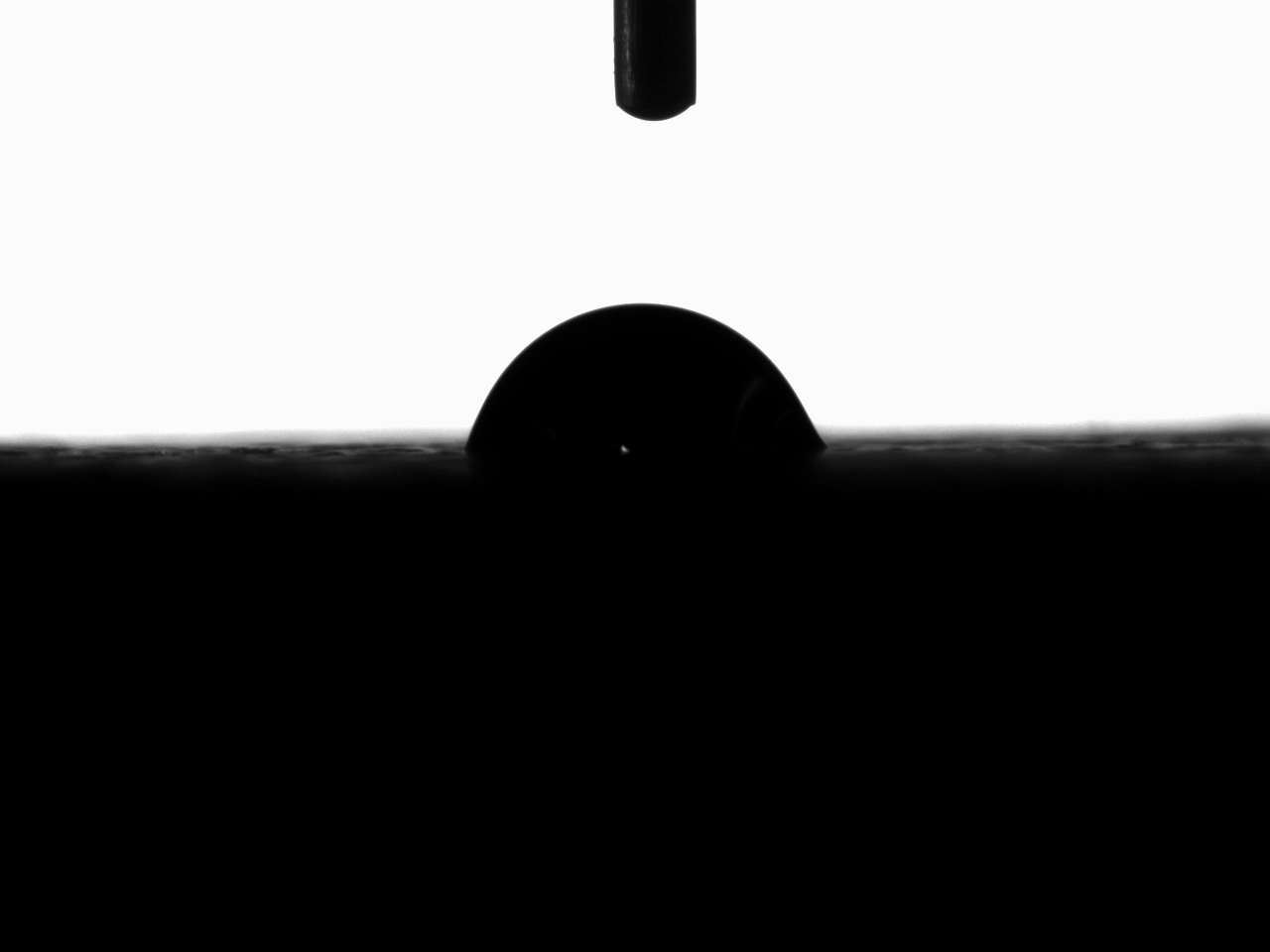

Supplement: Supplementary file 2 — Source Data [file 41467_2020_16847_MOESM2_ESM.zip › Source Data/SI/S11/S11c.bmp]

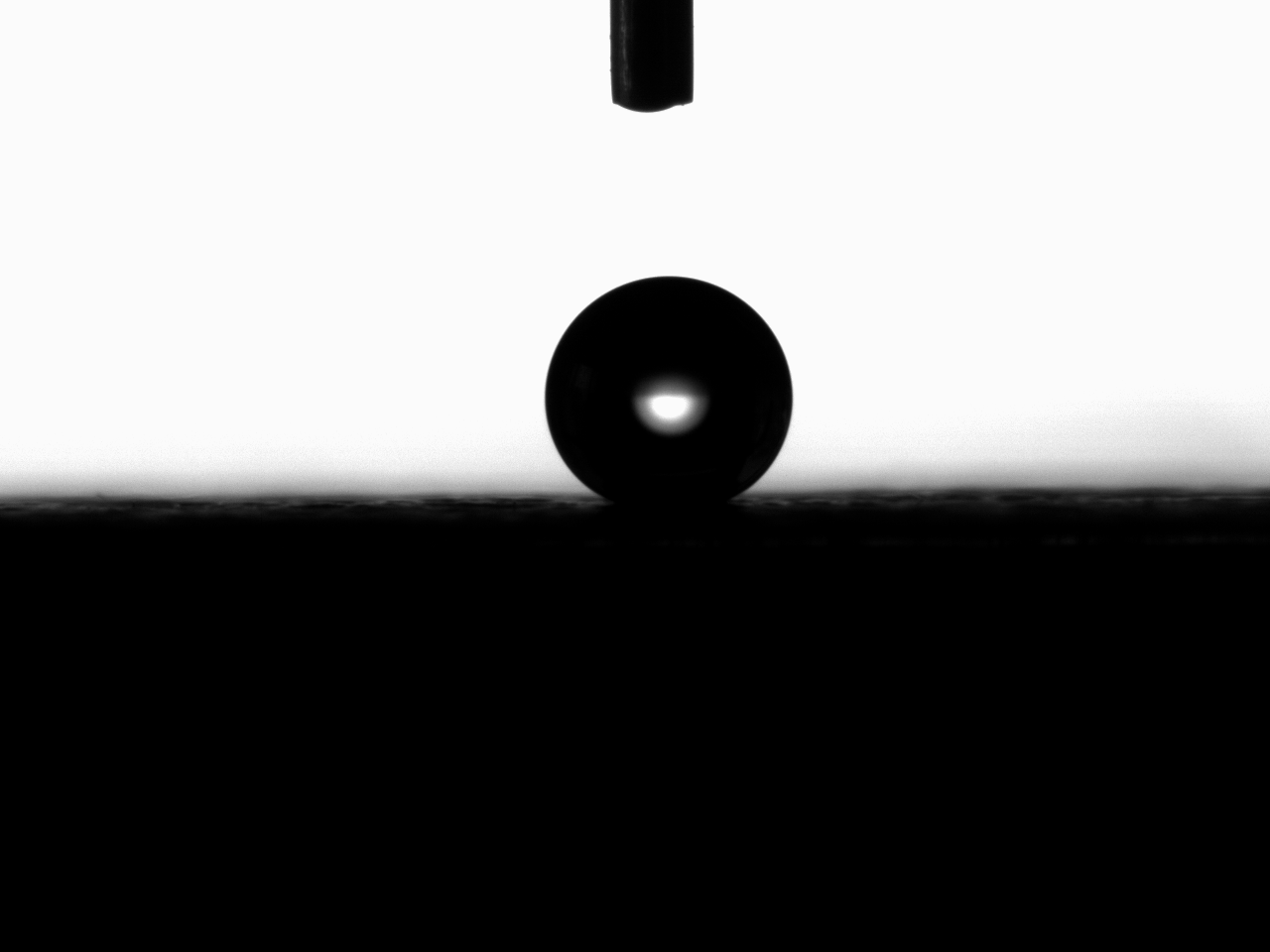

Supplement: Supplementary file 2 — Source Data [file 41467_2020_16847_MOESM2_ESM.zip › Source Data/SI/S11/S11d.bmp]

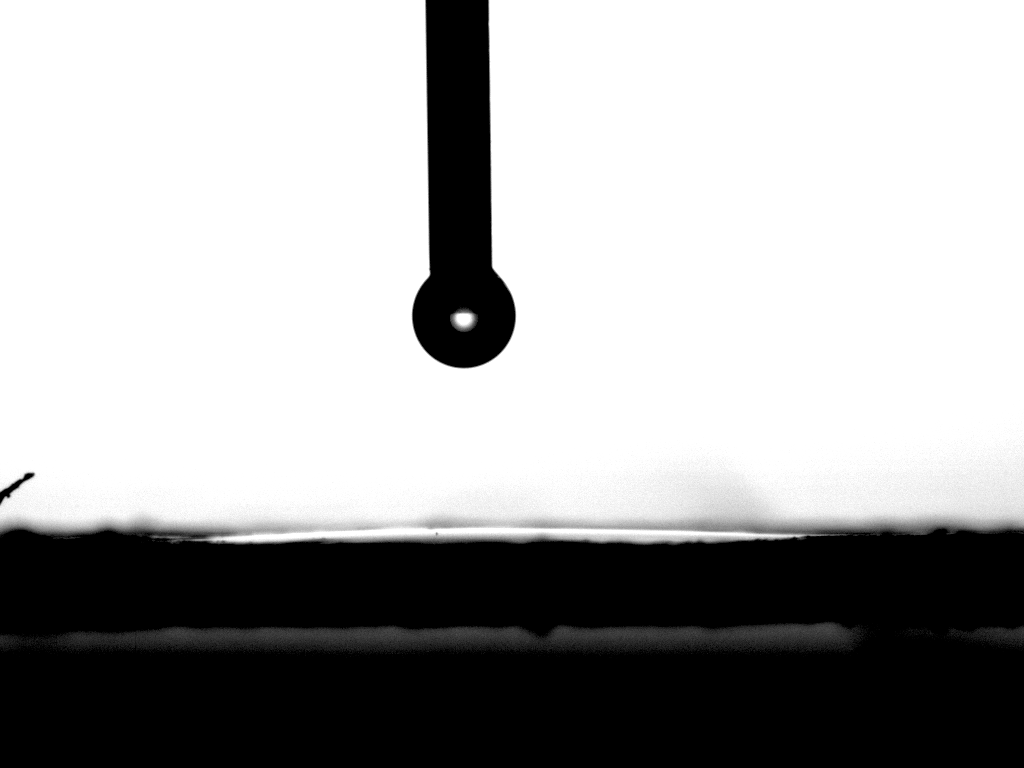

Supplement: Supplementary file 2 — Source Data [file 41467_2020_16847_MOESM2_ESM.zip › Source Data/SI/S11/S11e.bmp]

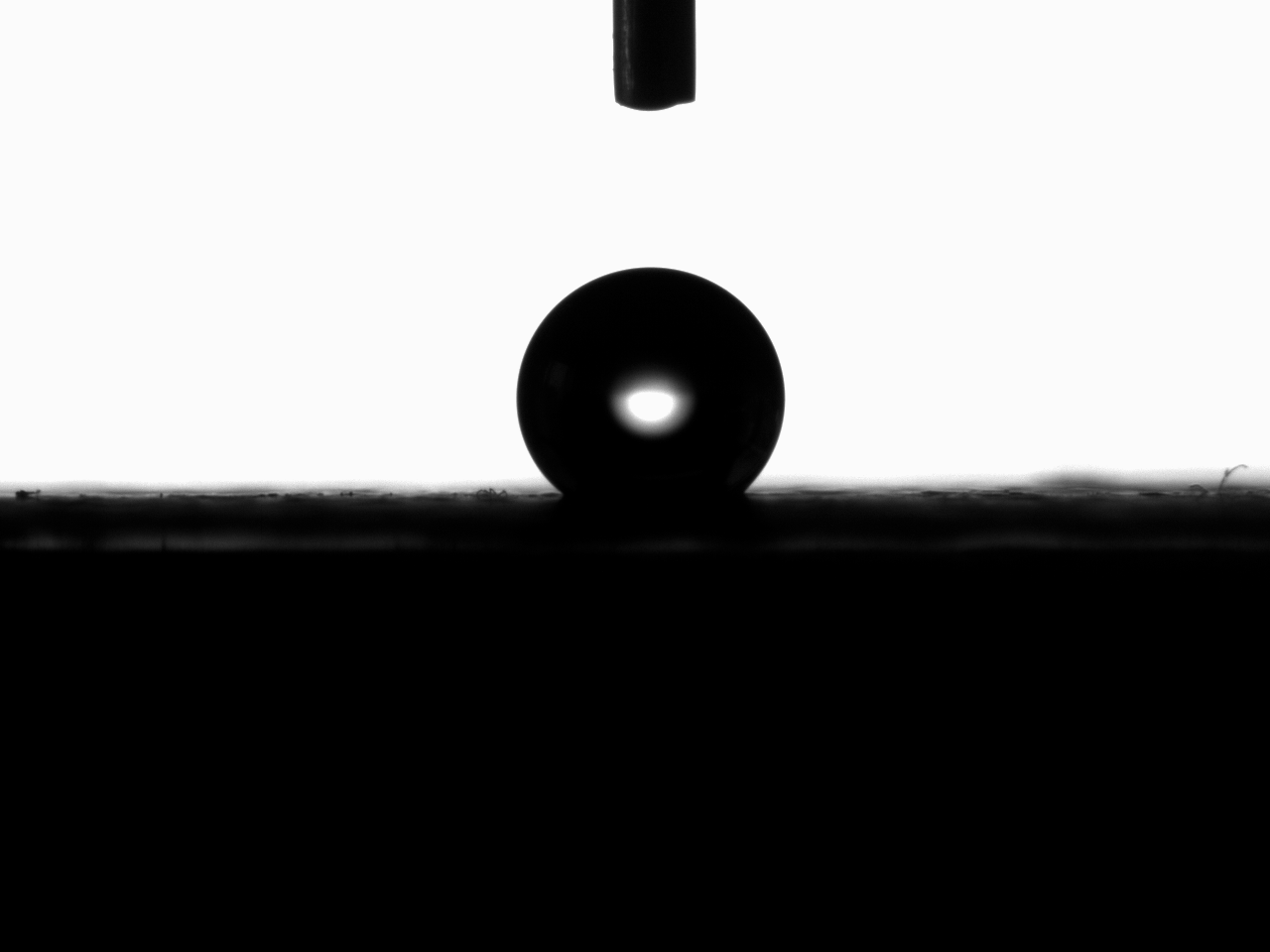

Supplement: Supplementary file 2 — Source Data [file 41467_2020_16847_MOESM2_ESM.zip › Source Data/SI/S11/S11f.bmp]

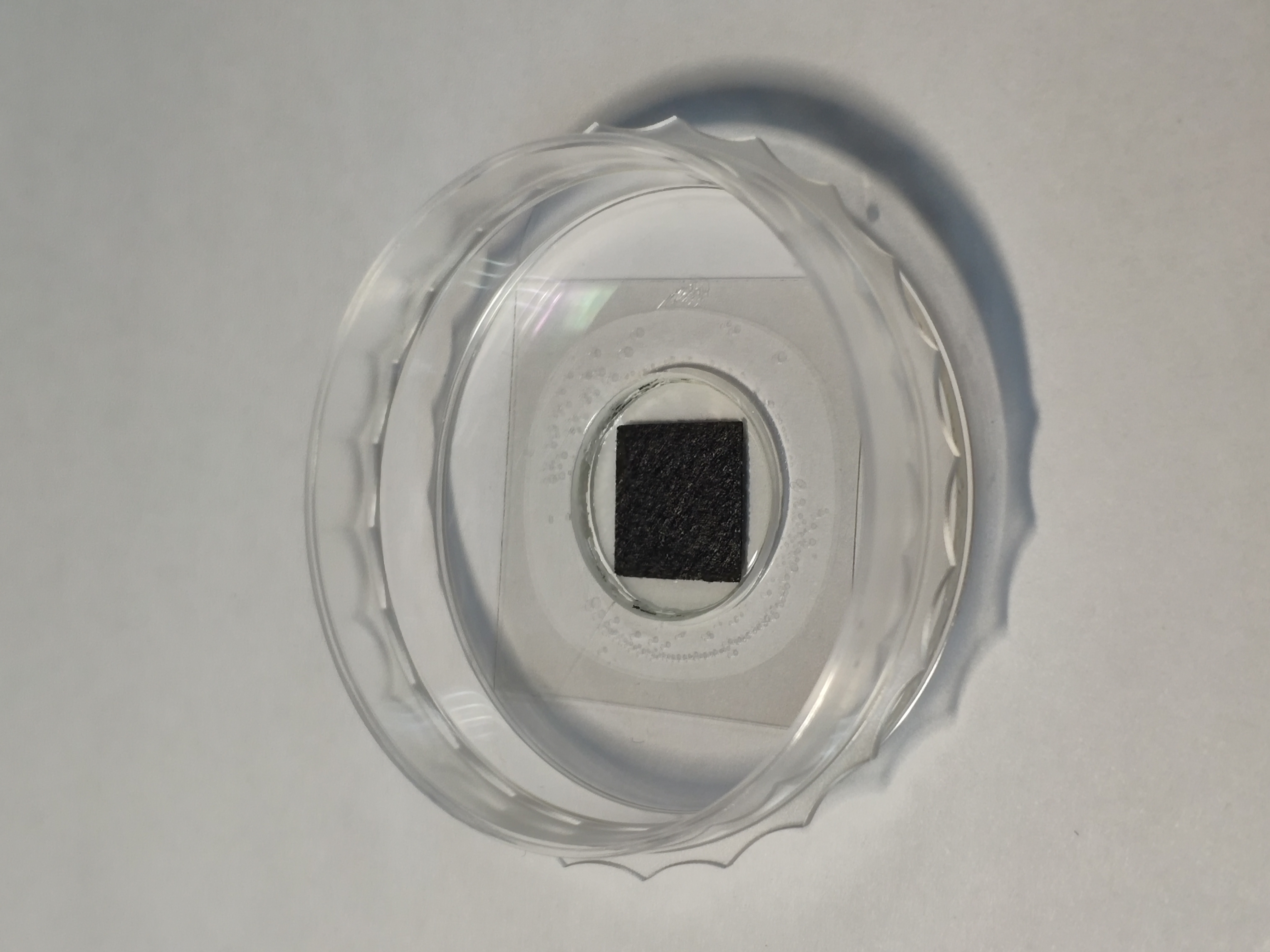

Supplement: Supplementary file 2 — Source Data [file 41467_2020_16847_MOESM2_ESM.zip › Source Data/SI/S12/S12b.jpg]

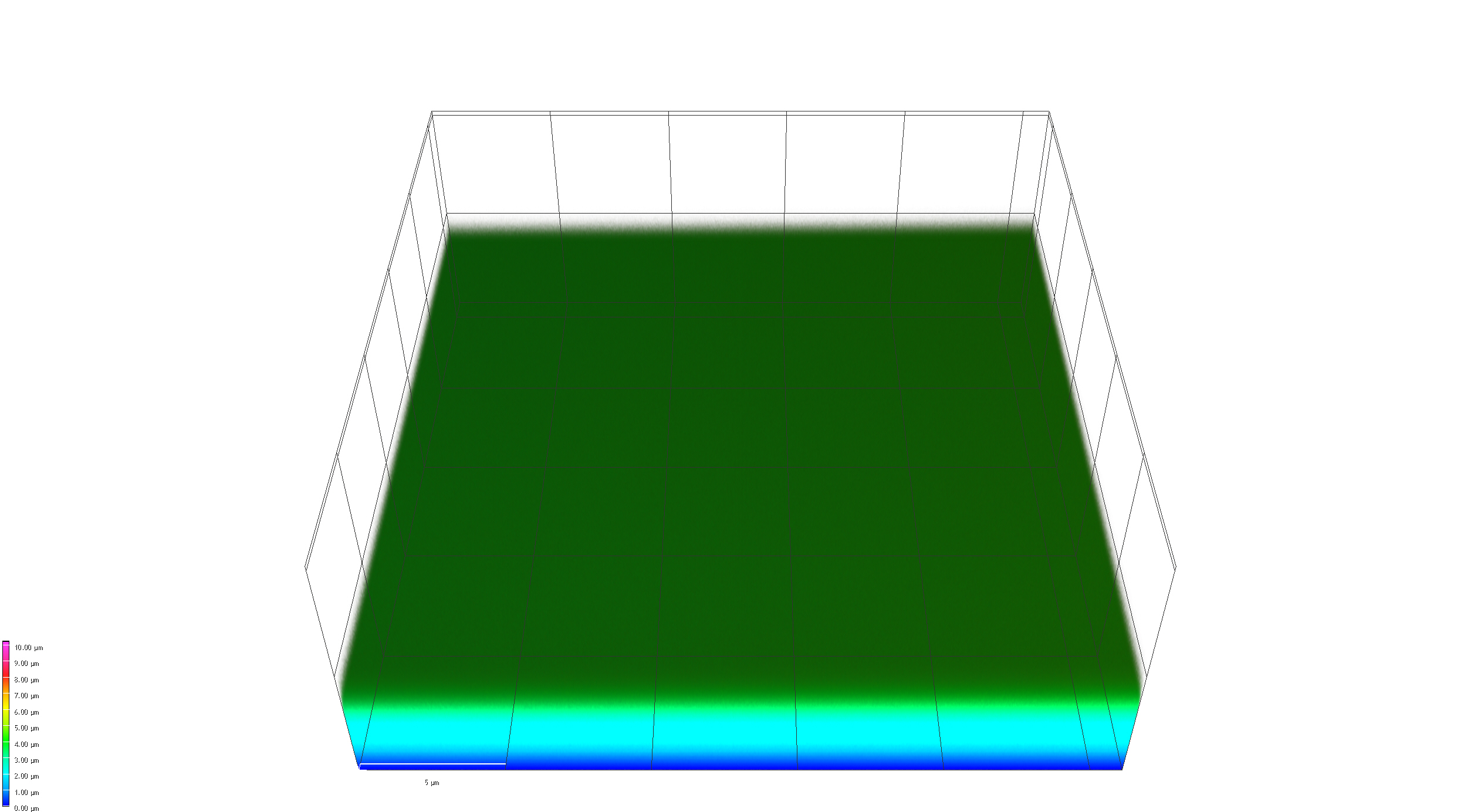

Supplement: Supplementary file 2 — Source Data [file 41467_2020_16847_MOESM2_ESM.zip › Source Data/SI/S13/S13a.jpg]

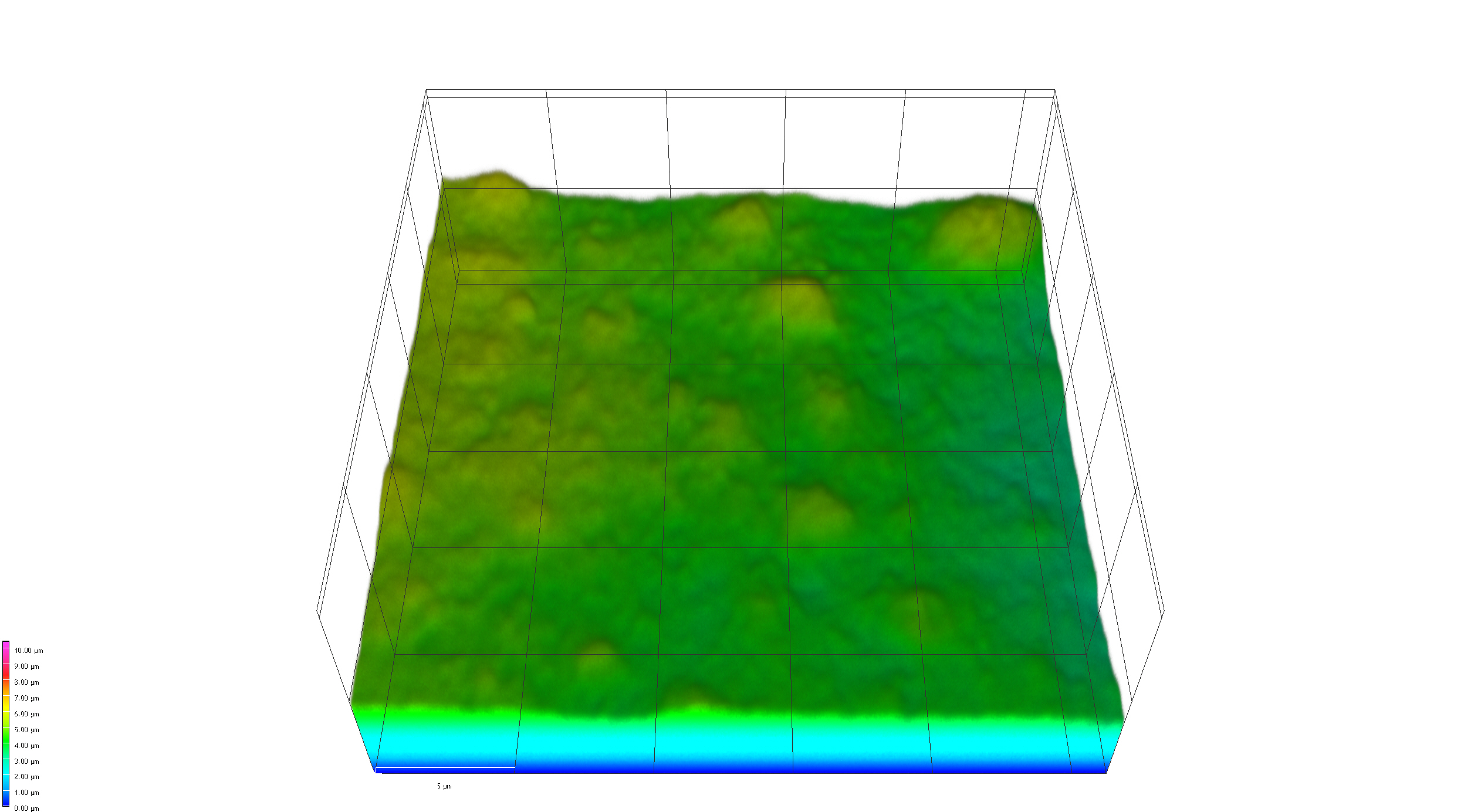

Supplement: Supplementary file 2 — Source Data [file 41467_2020_16847_MOESM2_ESM.zip › Source Data/SI/S13/S13b.jpg]

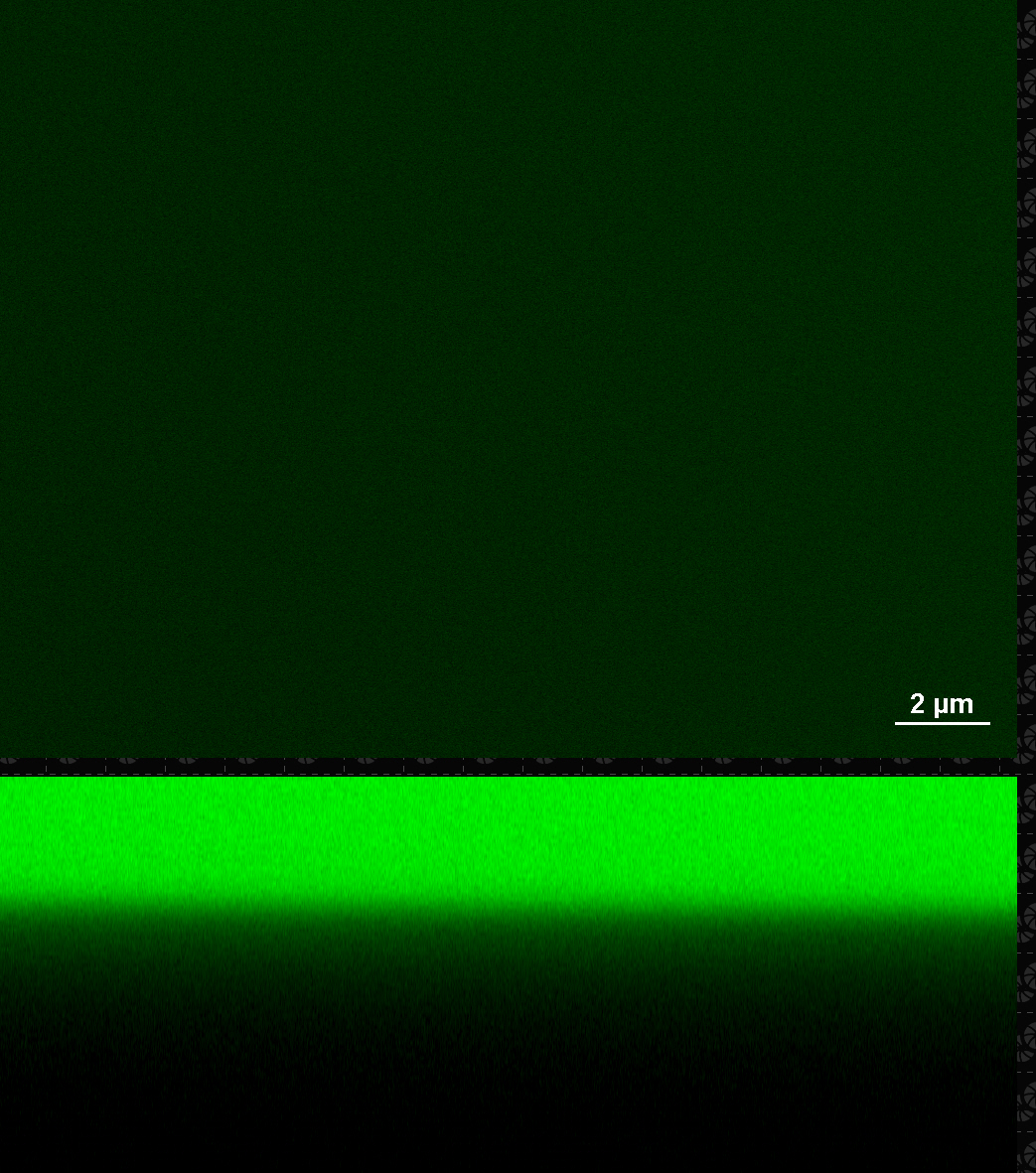

Supplement: Supplementary file 2 — Source Data [file 41467_2020_16847_MOESM2_ESM.zip › Source Data/SI/S13/S13c.jpg]

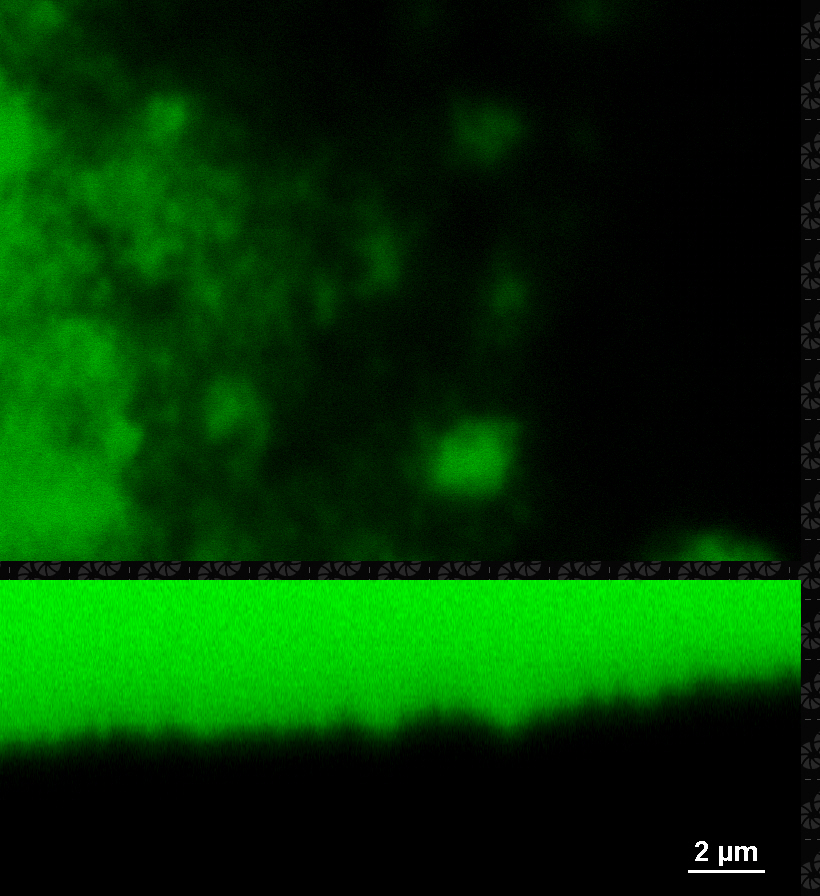

Supplement: Supplementary file 2 — Source Data [file 41467_2020_16847_MOESM2_ESM.zip › Source Data/SI/S13/S13d.jpg]

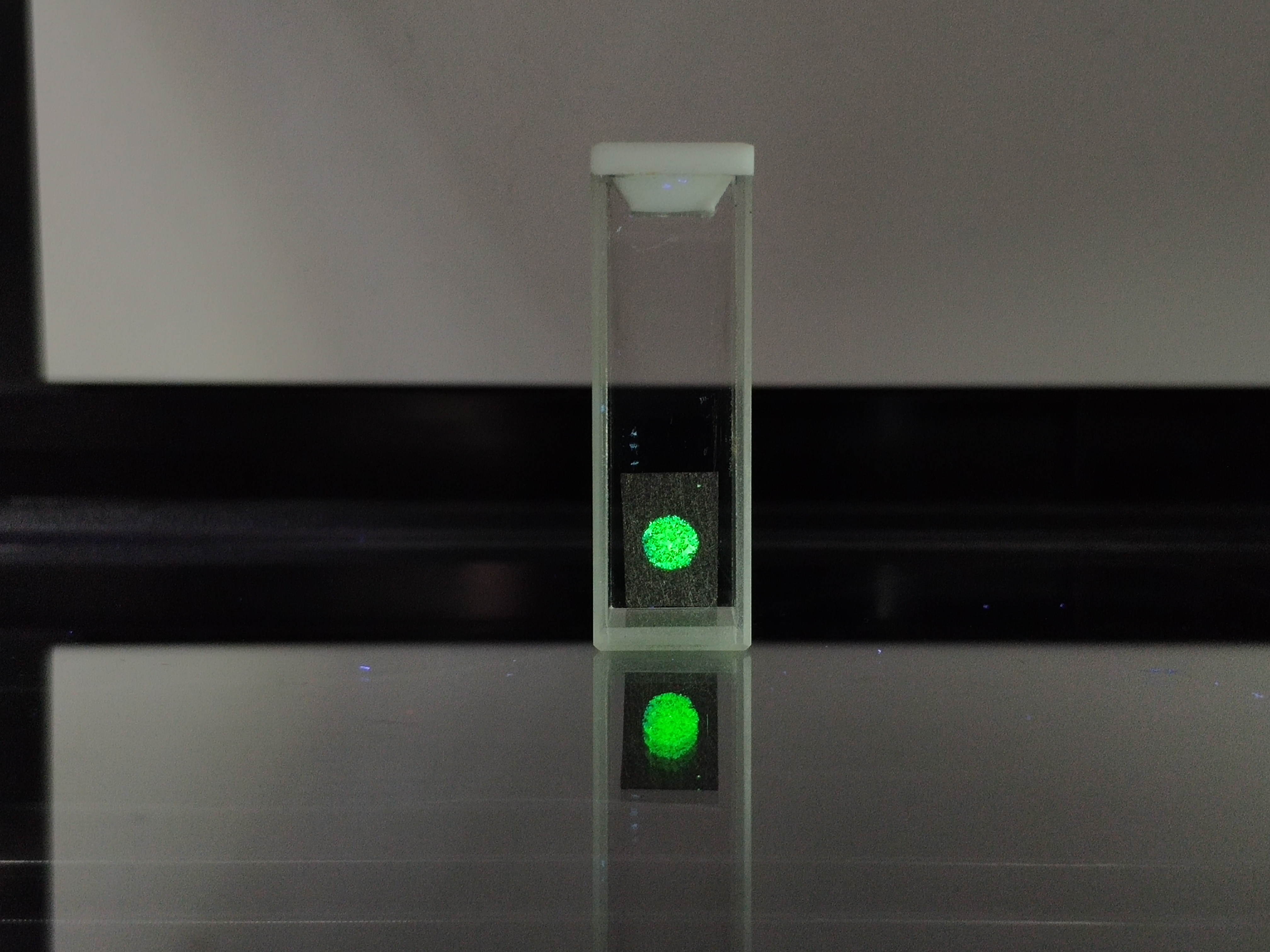

Supplement: Supplementary file 2 — Source Data [file 41467_2020_16847_MOESM2_ESM.zip › Source Data/SI/S16/S16a.jpg]

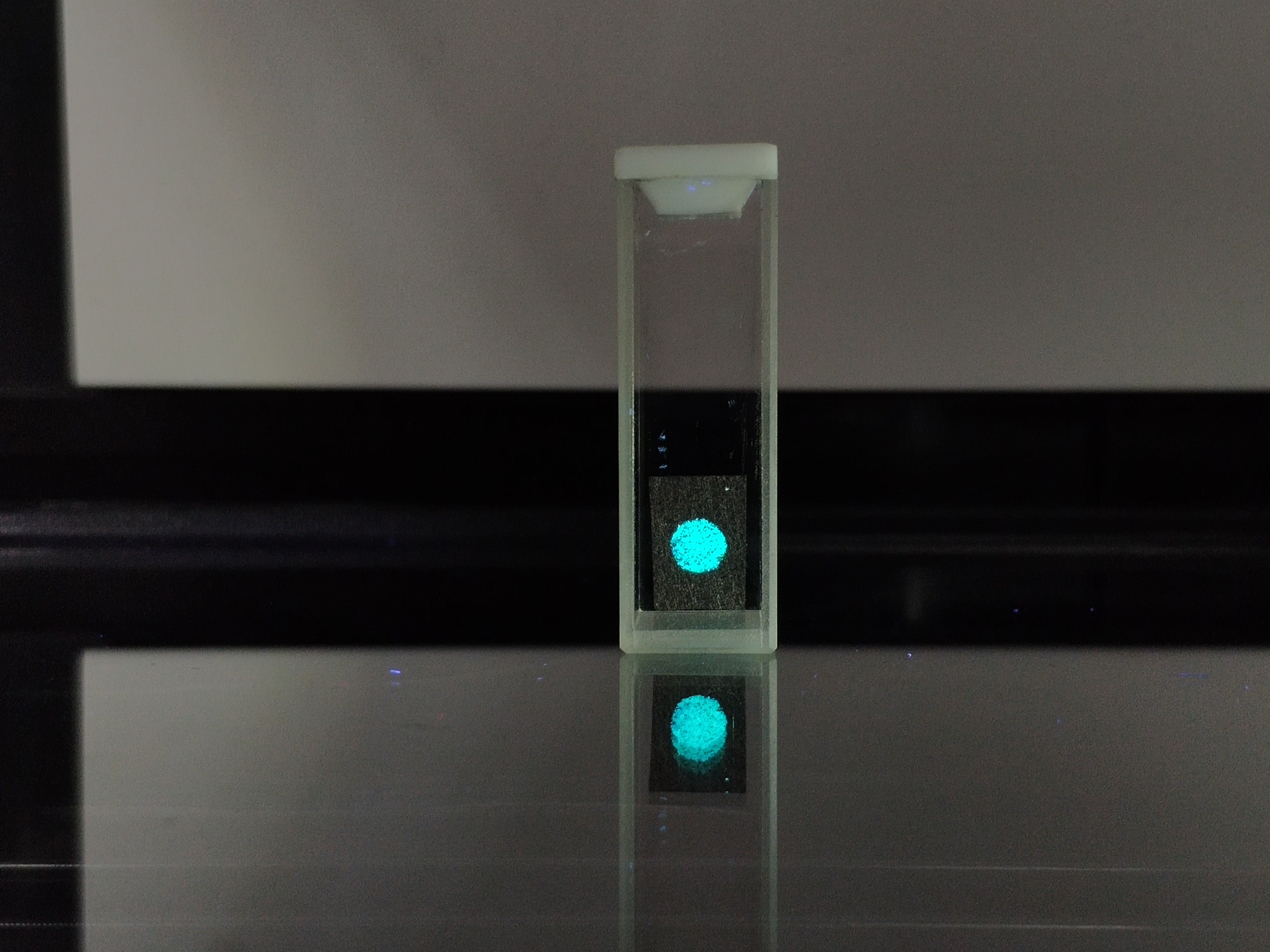

Supplement: Supplementary file 2 — Source Data [file 41467_2020_16847_MOESM2_ESM.zip › Source Data/SI/S16/S16b.jpg]

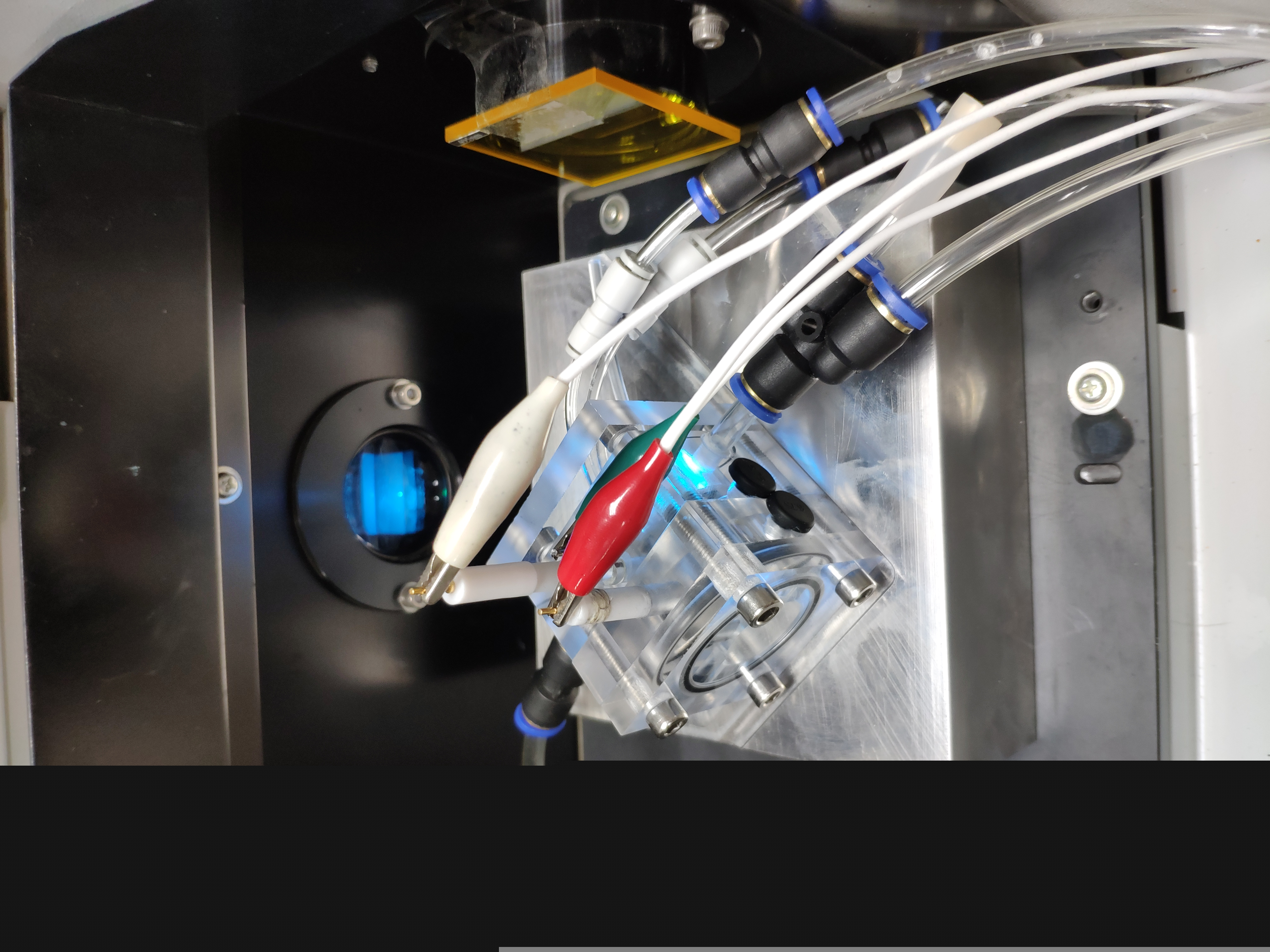

Supplement: Supplementary file 2 — Source Data [file 41467_2020_16847_MOESM2_ESM.zip › Source Data/SI/S16/S16c.jpg]

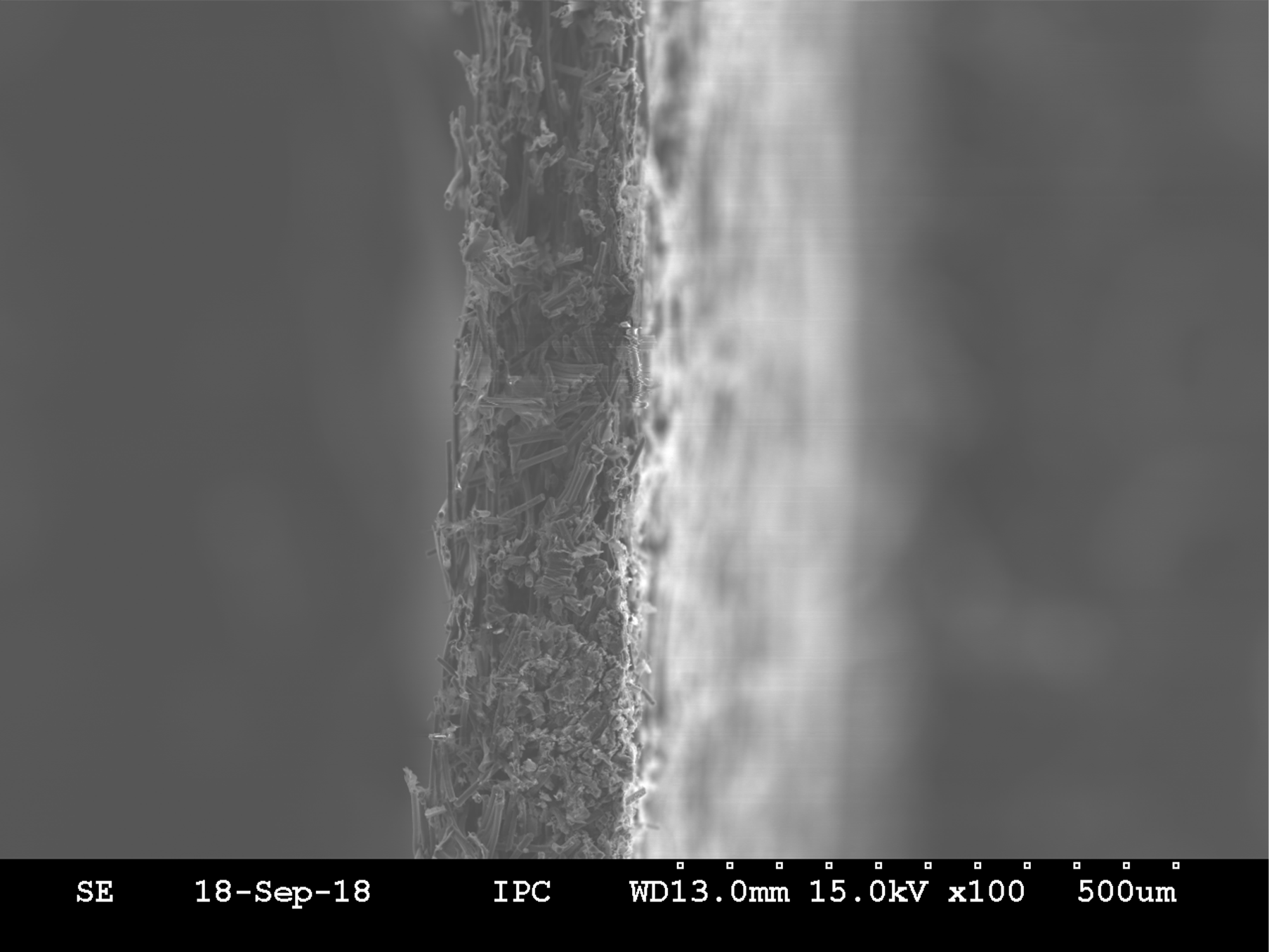

Supplement: Supplementary file 2 — Source Data [file 41467_2020_16847_MOESM2_ESM.zip › Source Data/SI/S2.tif]
